# Acupuncture therapies for post-stroke depression: the evidence mapping of clinical studies

**DOI:** 10.3389/fpsyt.2025.1523050

**Published:** 2025-03-04

**Authors:** Zhuo Zhou, Chao Ke, Wenying Shi, Yang Cao, Zhengrong Xie, Xi Zhao, Zeli Hu, Yilin Zhou, Wei Zhang

**Affiliations:** The First Hospital of Hunan University of Chinese Medicine, Changsha, China

**Keywords:** post-stroke depression, acupuncture therapies, moxibustion, evidence-based analysis, evidence mapping

## Abstract

**Background:**

Acupuncture-related therapies have been widely used in previous studies, of which the ones for post-stroke depression (PSD) is on the rise. This study aims to map the current clinical research landscape and identifies gaps to provide direction and information for future research.

**Methods:**

Eight databases were searched on acupuncture-related therapies for PSD from inception until April 2024. The publication profile, study objects, intervention categories, outcome indexes were graphically displayed. The Cochrane Collaboration’s bias risk assessment tool was used to independently assess randomized controlled trials (RCTs) quality, and the methodological quality of the systematic reviews were assessed using the AMSTAR 2 checklist.

**Results:**

A total of 666 clinical studies and 34 systematic reviews/Meta-analyses (SRs/MAs) were included in the evidence map, and the earliest report was found in 1996. The studies were mostly from China, and 89% of the evidence of the studies were of the RCTs. Body acupuncture and electroacupuncture were the most commonly used interventions. Most of the intervention durations were 2-4 weeks, and few patients were followed up. The main outcome was measured by effective rate and the Hamilton Rating Scale for Depression (HAMD). Evidences from clinical studies and SRs/MAs suggest that acupuncture has significant advantages in improving PSD, but the overall quality of studies could be improved.

**Conclusions:**

Acupuncture-related therapies have great prospect in relieving the clinical symptoms of PSD, although there are some design and methodological defects in the current studies. In the future, the quality of research needs to be improved for the robustness of clinical evidence.

## 1 Introduction

Stroke is a cardiovascular disease with high morbidity, mortality and disability, which usually leads to a series of severe sequelae and affects the quality of life of patients (1). Post-stroke depression (PSD) is the most common neuropsychiatric disorder in post-stroke patients characterized by persistent decreased mood and interest (2). The global incidence rate ranges from 27.5% to 62.5% and shows an increasing trend (3), which brings great mental burden and economic pressure to patients and their families. Nevertheless, PSD is still considered to be the most overlooked symptom after stroke, and underdiagnosis and undertreatment are common (4). A large amount of evidence shows that PSD seriously affects the rehabilitation of motor and cognitive function, leading to further decline in the quality of life. What’s worse, to a certain extent, it can also increase the risk of recurrence of neurovascular events, which is closely related to high mortality (5, 6). On the contrary, good mood can greatly promote the recovery of patients (7).

However, the current treatment methods for PSD have not achieved a completely satisfactory ideal effect. Its treatment is mainly divided into drug and non-drug therapies. Selective serotonin reuptake inhibitors (SSRIs) or norepinephrine reuptake inhibitors (SNRIs) are the first-line pharmacologic therapies. However, comorbidities may lead to more adverse effects or contraindications when using antidepressants in elderly PSD patients, due to the presence of stroke pathology (8). At the same time, all commonly used antidepressants are associated with a significantly increased risk of adverse outcomes including seizures, falls, suicide, bleeding complications, and even death (9–12).Non-drug therapies such as virtual reality (VR), cognitive behavioral therapy (CBT), repetitive transcranial magnetic stimulation (rTMS) and unconventional therapy can also improve PSD (13). However, a RCT study shows that CBT was ineffective in the treatment of PSD (14). traditional Chinese medicine (TCM) treatment can improve the symptoms of neurological deficit and mental state through multiple pathways and multiple targets, and has few adverse reactions. Its development prospects and advantages are good (15).

As an economic and safe traditional Chinese medicine intervention, acupuncture related therapy has been widely used in clinical treatment of nervous system and mental diseases because of its exact effect. A network meta-analysis suggests that acupuncture may be the most effective nonpharmacologic treatment for improving PSD (12). Previous studies have found that acupuncture can improve depressive symptoms by regulating neurotransmitter signal transmission, inhibiting inflammatory response and regulating HPA axis hyperfunction (16). Moreover, clinical study has shown that EA is an effective alternative to escitalopram in the treatment of mild to moderate PSD (17).Unfortunately, there is a lack of a comprehensive study that integrates all the clinical evidence to provide support for better treatment of PSD in the future.

Evidence map is a new evidence integration tool. Through comprehensive search and scientific analysis, it systematically summarizes, combs and analyzes the current research in related fields. The research progress and existing problems in this field are presented in a multi-directional, multi-dimensional and multi-perspective way (18). For instance, Qing Su et al. reported on the clinical research regarding the intervention of traditional Chinese medicine (TCM) therapy in post - stroke sleep disorders (PSSDs). It was discovered that Chinese medicine (CHM) and acupuncture had been extensively investigated; however, the quality of these studies required enhancement (19). Additionally, a systematic review of TCM interventions for cognitive and motor rehabilitation in stroke patients revealed that acupuncture was advantageous in the treatment of shoulder - hand syndrome (20). For all we know, no evidence of acupuncture therapies for PSD has been presented in previous studies. Our study fills this evidence gap by establishing an evidence map for acupuncture related interventions in PSD based on currently published clinically relevant studies. It not only provides a complete picture of the research in this field for evidence users, but also provides future research directions for decision makers, clinicians, guideline makers, researchers and patients.

## 2 Materials and methods

### 2.1 Search strategy

We systematically searched eight Chinese and English databases including PubMed, Embase, Cochrane Library, Web of Science (WOS), China National Knowledge Infrastructure(CNKI), Chinese Biological Medicine (CBM), VIP Database for Chinese Technical Periodicals (VIP), and Wanfang database, for clinical primary studies and systematic reviews/Meta-analyses (SRs/MAs) of acupuncture related therapies for PSD published from the establishment of the database to April 2024.The literature search was performed with key search terms and their potential combinations of “stroke”, “depression”, and “acupuncture” as the main topic words and free words. All search strategies are described in **Supplementary Appendix 1**.

### 2.2 Inclusion and exclusion criteria

#### 2.2.1 Type of studies

Clinical studies and SRs/MAs of acupuncture related therapies for PSD were included in our analysis. If the same research team produced multiple systematic reviews on the same clinical problem, the latest version was included. We exclude the following situations: (1) animal experiments; (2) non-clinical studies: conference abstracts, commentaries, opinions, expert experiences, research progress, overview, bibliometrics, etc.; (3) Reevaluation of systematic reviews.

#### 2.2.2 Study participants

(1) Stroke was diagnosed by imaging data or the guidelines, regardless of stroke type; (2) it had been diagnosed with post-stroke depression (with any degree of depression); or (3) it was used to assess the preventive effect of treatment on depression in stroke patients; (4) participants with other comorbidities or symptoms were excluded.

#### 2.2.3 Interventions

Participants who received all acupuncture-related therapies included in the search strategy, including manual acupuncture, electroacupuncture, moxibustion, acupoint application, auricular point application, acupoint injection, and transcutaneous electrical acupoint stimulation (TEAS). All interventions mentioned above were used alone or in combination with other acupuncture or non-acupuncture therapies. Studies that did not act on meridian acupoints were excluded.

Since PSD patients usually need to receive conventional treatment based on western medicine for stroke, our study only focused on the treatment of post-stroke depression. The conventional treatment was regarded as a blank control and was not included in the intervention statistics. The control group included blank, different acupuncture and moxibustion therapies, sham acupuncture, drug and non-drug treatment. If both the observation group and the control group used a positive treatment other than acupuncture, it had to be consistent between the two groups. Studies in which the intervention group used the same acupuncture intervention as the control group and in which acupuncture was not the only variable were excluded.

### 2.3 Literature selection and data extraction

#### 2.3.1 Studies selection process

In this study, two reviewers (ZZ and ZYL) separately conducted a preliminary literature search of the above databases. Subsequently, the literature was imported into Endnote X9 software to remove duplicate literature and preliminarily screened according to the title and abstract of the article in the software. Then, after reading the full text, all literatures that met the inclusion and exclusion criteria were clearly identified, and relevant information was extracted. Data extraction and management were conducted by two reviewers (KC and SWY) through Microsoft Excel 2021, and the main content included various aspects such as basic information on the studies (title, publication, author information, etc.), characteristics of the trials (study design, sample size, etc.), participant details (age, gender, diagnosis, duration, etc.), method of intervention/control (intervention means, frequency, duration of a session, etc.), outcome measurements. Disagreements between studies were resolved by discussion or consultation with a third reviewer (ZW) until consensus was reached.

#### 2.3.2 Data analysis and generation of evidence maps

The information of all included studies was extracted and analyzed. The results were presented in the form of text description and graphs supplemented by supplementary forms, including tables, line charts, bar charts, pie charts and bubble charts, etc., to help a more thorough understanding of the distribution and status of key evidence, and to fully understand and make good use of this evidence chart. All statistical analyses were performed using Microsoft Excel 2021, and the software used for graphics production included Microsoft Excel 2021, IBM SPSS Modeler 18.0, and adobe illustrator2022. In addition, Sankey plot is the use of biomedical data analysis and visualization of integrated network service ChiPlot (https://www.chiplot.online/).

### 2.4 Methodological quality assessment

Two reviewers (CY and XZR) independently assessed the methodological quality of the final included RCTS using the Risk of Bias Tool (ROB) in Cochrane Handbook (5.1.0). This tool mainly includes the generation of random sequences; Allocation concealment, blinding of participants and personnel, blinding of outcome assessments, completeness of outcome data, selective reporting of study results, and other potential biases were evaluated. The evaluation was graded into one of the three categories: low risk, high risk or unclear risk of bias.

The methodological quality of the included SRs/MAs was still assessed by two reviewers (ZX and HZL) using the AMSTAR (Assessment of Multiple Systematic Reviews) tool. The instrument consists of items in 16 methodological areas, classified as “Yes” “No” or “Partly yes”. Item 2, 4, 7, 9, 11, 13 and 15 were considered as key items. Overall confidence in SRs/MAs results was categorized into four levels: high, moderate, low, or very low (21).

## 3 Results

### 3.1 Selection of sources of evidence

**Figure 1:**
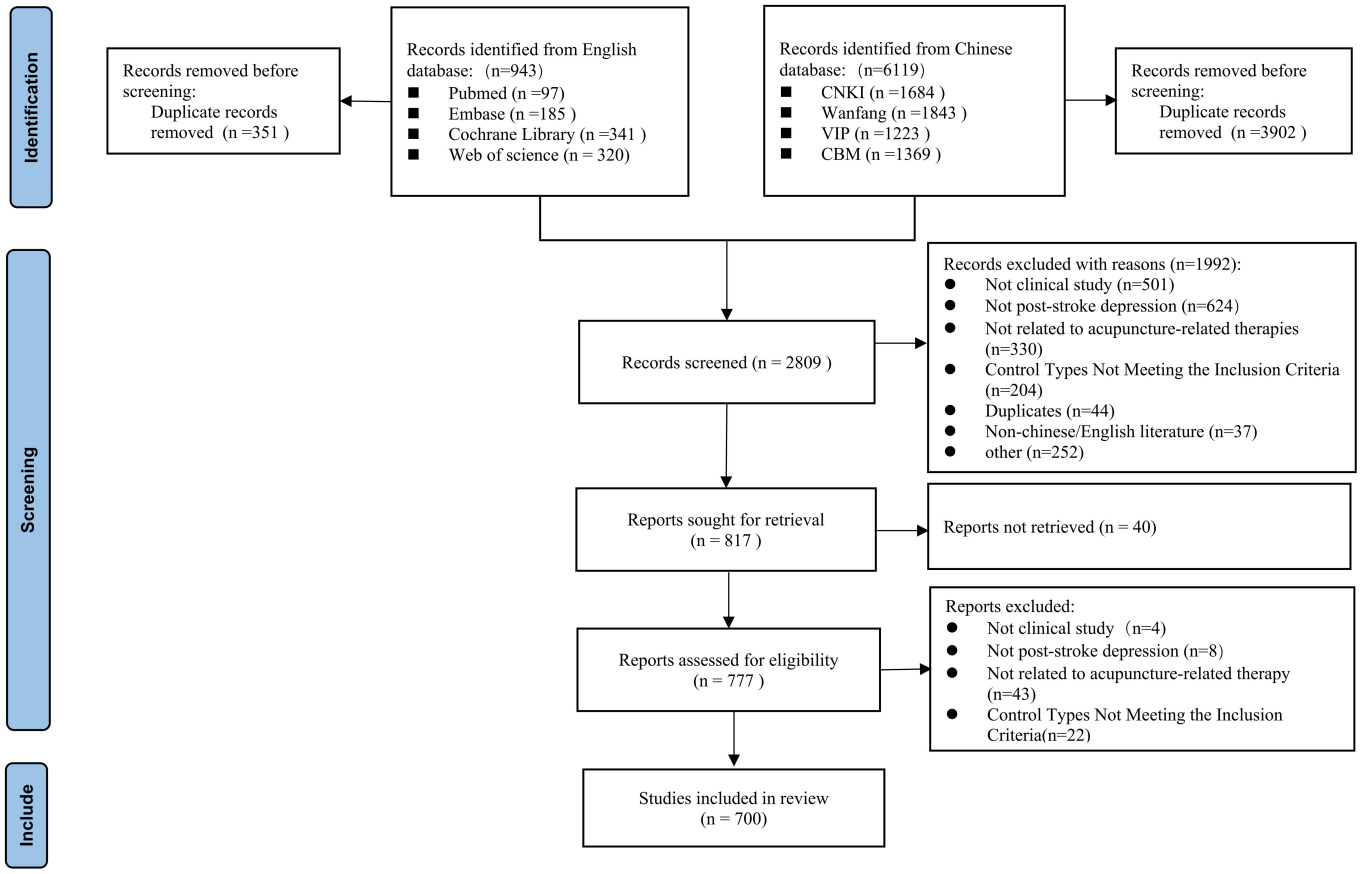
Flow diagram representing the process of literature screening (20).

A total of 7062 studies on acupuncture related therapies for PSD were identified, of which 700 eligible studies were finally included in the evidence map. A total of 666 clinical research articles (651 in Chinese, 15 in English) and 34 SRs/MAs articles (25 in Chinese, 9 in English) were included. The identification and selection process of the study is illustrated in **Figure 1** (22). A complete list of the final included studies is provided in **Supplementary Appendix 2**.

### 3.2 Basic characteristics of evidence sources

**Figure 2:**
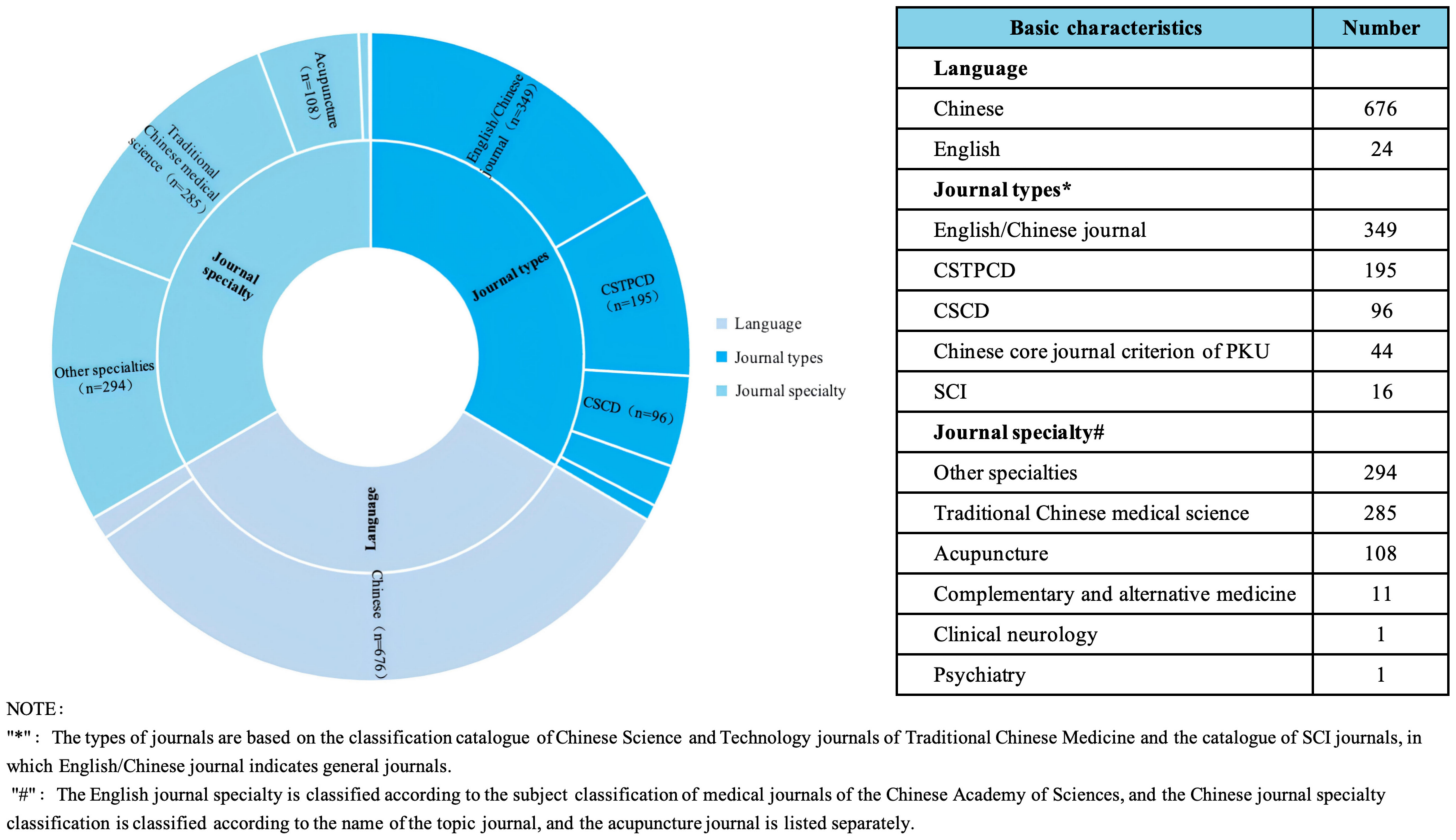
Basic characteristics of the source of evidence.

Of the 700 articles included in this study, 676 were in Chinese. Of the few studies from English journals, only 16 were Science Citation Index journal (SCI). The included studies were widely published in traditional Chinese medicine (TCM) journals (285,40.7%) and other type journals (294,42.0%). The basic characteristics of the sources of evidence are detailed in **Figure 2**.

**Figure 3:**
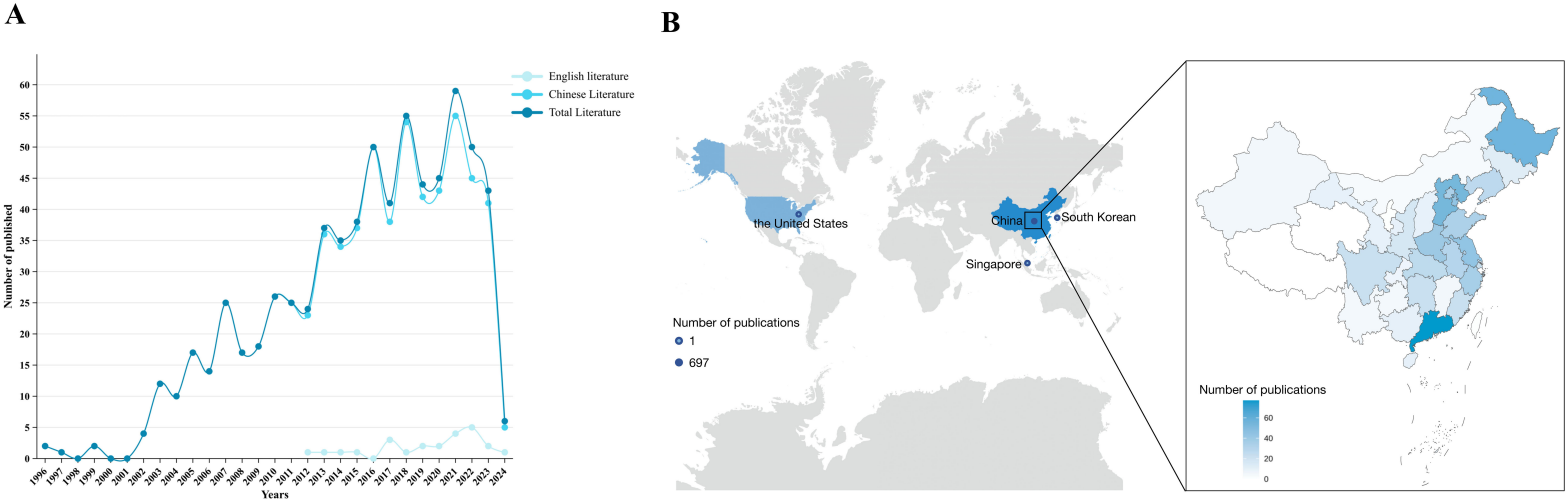
Annual trends and geographical distribution map of publication. **(A)** Year of publication of acupuncture-related therapies in PSD. **(B)** Regional distribution of articles on acupuncture and moxibustion related therapy intervention for PSD.

**Figure 3A** shows the literature publication since the establishment of the database, and the overall number of published papers shows an upward trend. The first published study on acupuncture and moxibustion intervention for post-stroke depression can be traced back to 1996, and the first English study was published in 2012. The number of publications peaked in 2021, with a total of 59 studies. A total of 6 studies were published between January and April 2024. According to the figure, the number of articles published in Chinese is significantly higher than that in English. In terms of the source of countries, only two were from international cooperation. Korea, Singapore and the United States each published one study, while China accounted for 99.6% of all studies, totaling 697 studies. In terms of the source of provinces in China, Guangdong and Heilongjiang were the provinces with the most studies on acupuncture intervention for post-stroke depression. In terms of regional cooperation, 202 studies were multi-institution cooperation, but only 27 studies were cross-regional cooperation (**Figure 3B**, **Supplementary Appendix 3**).

### 3.3 Evidence of clinical primary research

**Figure 4:**
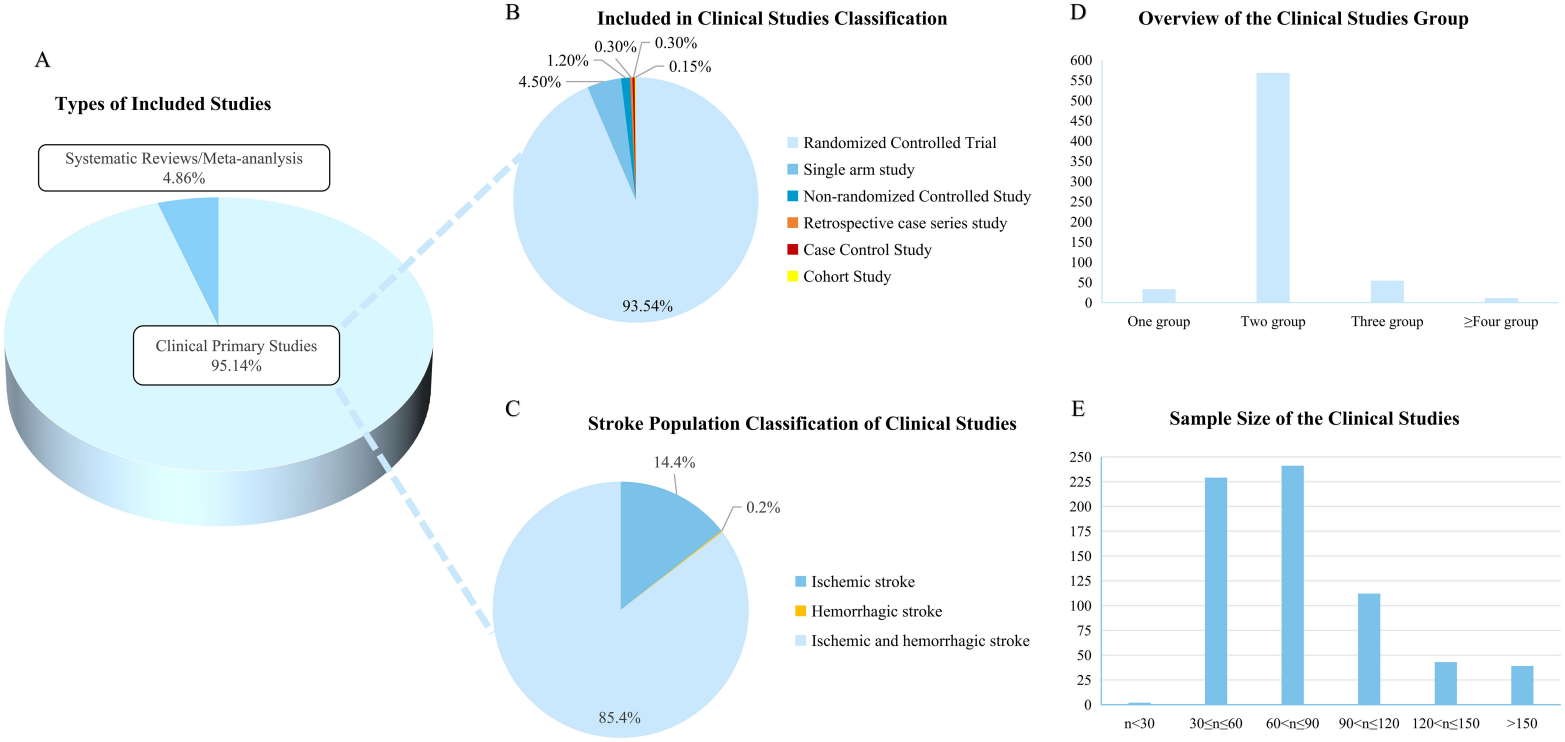
Basic characteristics of the study population. **(A)** Types of included studies; **(B)** Included in primary studies classification. Different colors indicate different study types; **(C)** Stroke population classification of clinical studies; **(D)** Overview of the clinical studies group; **(E)** Sample size of the clinical studies.

A total of 34 SRs/MAs were included in this study, and the rest were primary clinical studies (666,95.14%), including 623 randomized controlled studies (RCTs), 30 single-arm studies, and 8 non-randomized controlled studies. In addition, there were 2 retrospective case series studies, 2 case-control studies and 1 cohort study (**Figures 4A, B**).

#### 3.3.1 Evidence characteristics of the study participants

A total of 666 original clinical studies on acupuncture for post-stroke depression involving 65,671 people were included. Most of the studies were conducted in two subgroups (**Figure 4D**). The largest sample size study was a retrospective study with a sample size of 8487 people (23). In randomized controlled studies, the largest sample size was 500 participants and the smallest was only 21 subjects (24, 25). Studies with the number of participants between 30 and 90 were the most abundant, accounting for 70.57% (**Figure 4E**).

Stroke was divided into ischemic stroke and hemorrhagic stroke. A total of 569 studies on both ischemic and hemorrhagic stroke were included, accounting for 85.4%. Ischemic stroke population accounting for about 14.4%, and only one study on hemorrhagic stroke population (**Figure 4C**). Among the stroke population, 97 studies explicitly included patients with first stroke, and 249 studies were diagnosed with stroke by imaging data.

Among all the clinical literature inclusion criteria, 260 studies did not mention the western medical diagnostic criteria for stroke. The most widely used diagnostic criteria were “*Various types of cerebrovascular disease diagnosis*” (270,40.5%) and “ *Chinese Guidelines for the Diagnosis and Treatment of Acute Ischemic Stroke*” (31,4.7%). “*The International Classification of Diseases(ICD) -10* “ published by World Health Organization (WHO) is commonly used in the international guidelines for stroke. The most widely used international diagnostic criteria for stroke was the International Classification of Diseases (10th edition) published by WHO. For the diagnosis of depression, the most used guidelines were “*the Chinese Classification and Diagnostic Criteria of Mental Disorders*” (348, 50.3%). 34 studies used “*Diagnostic and Statistical Manual of Psychiatry*” published by American Psychiatric Association (APA), and 18 studies were evaluated using the classification of mental and behavioral disorders of ICD-10 diagnostic criteria. The Hamilton Rating Scale for Depression (HAMD) was the most commonly used scale to evaluate the degree of depression in the included population, but 209 studies did not mention the version of it, resulting in inability to judge the degree of depression. In addition, the Self-Rating Depression Scale (SDS) is also used to diagnose depression. Because the study was mainly conducted in China, the Chinese guidelines were used in most of the diagnostic criteria.

A total of 514 studies did not mention any TCM diagnostic criteria. *The Criteria for Diagnosis and Efficacy Evaluation of Stroke* and *the Criteria for Diagnosis and Efficacy of TCM Diseases and Syndromes* were the most frequently used TCM diagnostic guidelines to guide the TCM diagnosis and dialectical classification of stroke. Among all the original clinical studies, only 73 studies made a dialectic classification of the included population, and the most common type was liver stagnation and qi stagnation, followed by heart and spleen deficiency and liver stagnation and spleen deficiency.

#### 3.3.2 Treatment regimens of clinical studies

**Figure 5:**
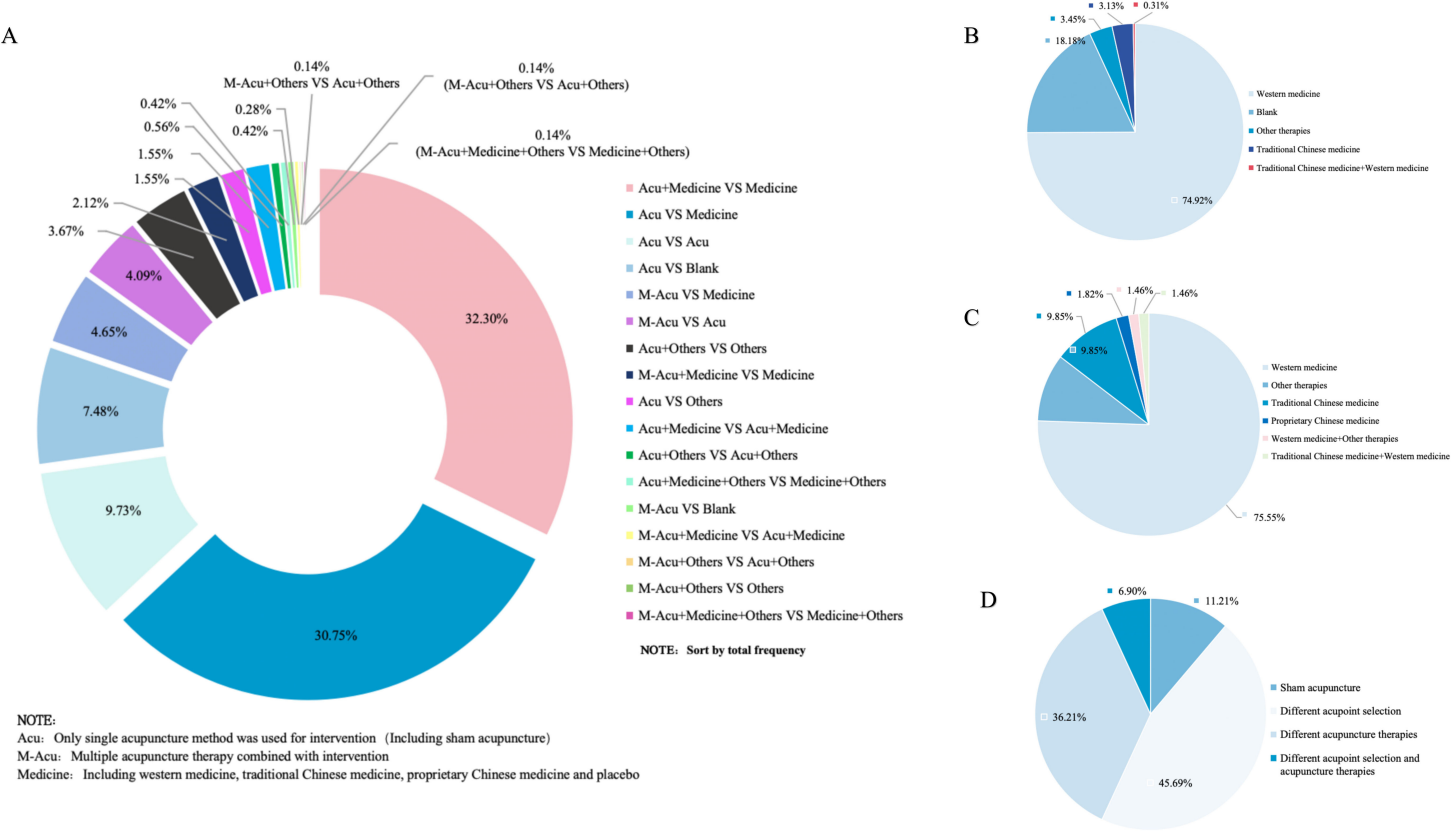
Overview of randomized controlled study design. **(A)** Design of intervention and control groups. **(B)** Study design to evaluate the efficacy of acupuncture versus other therapies; **(C)** Study design to evaluate the synergistic effects of acupuncture with other therapies; **(D)** Study designs to evaluate the effects of different acupuncture therapies.

A total of 623 RCTs included in the study were analyzed. If the study involved multiple groups, the eligible studies were divided for statistics. So we finally analyzed 709 and **Figure 5A** presents the RCTs profile. From the perspective of interventions and control design, single acupuncture therapy plus western medicine was most commonly used compared with western medicine alone (229,32.30%). In addition, single acupuncture therapy versus western medicine, and comparison of different acupuncture therapies were also commonly used in studies of acupuncture intervention for PSD.

The 709 control groups were grouped according to study purpose into (1) evaluation of the efficacy of acupuncture VS other therapies (**Figure 5B**); (2) assessment of synergy between acupuncture and other therapies (**Figure 5C**); and (3) evaluation of the effects of different acupuncture therapies (**Figure 5D**). In the study of acupuncture therapies VS other therapies, the blank control group was most widely used, accounting for 74.92% (the blank control group did not receive any other intervention on the premise of conventional treatment). When studying the synergy of acupuncture related therapies with other therapies, more attention is paid to the therapeutic effect of acupuncture therapies combined with antidepressants on PSD. Finally, the different acupoint selection methods and the different choice of acupuncture therapies were used the most in the evaluation of the efficacy of different acupuncture therapies.

**Figure 6:**
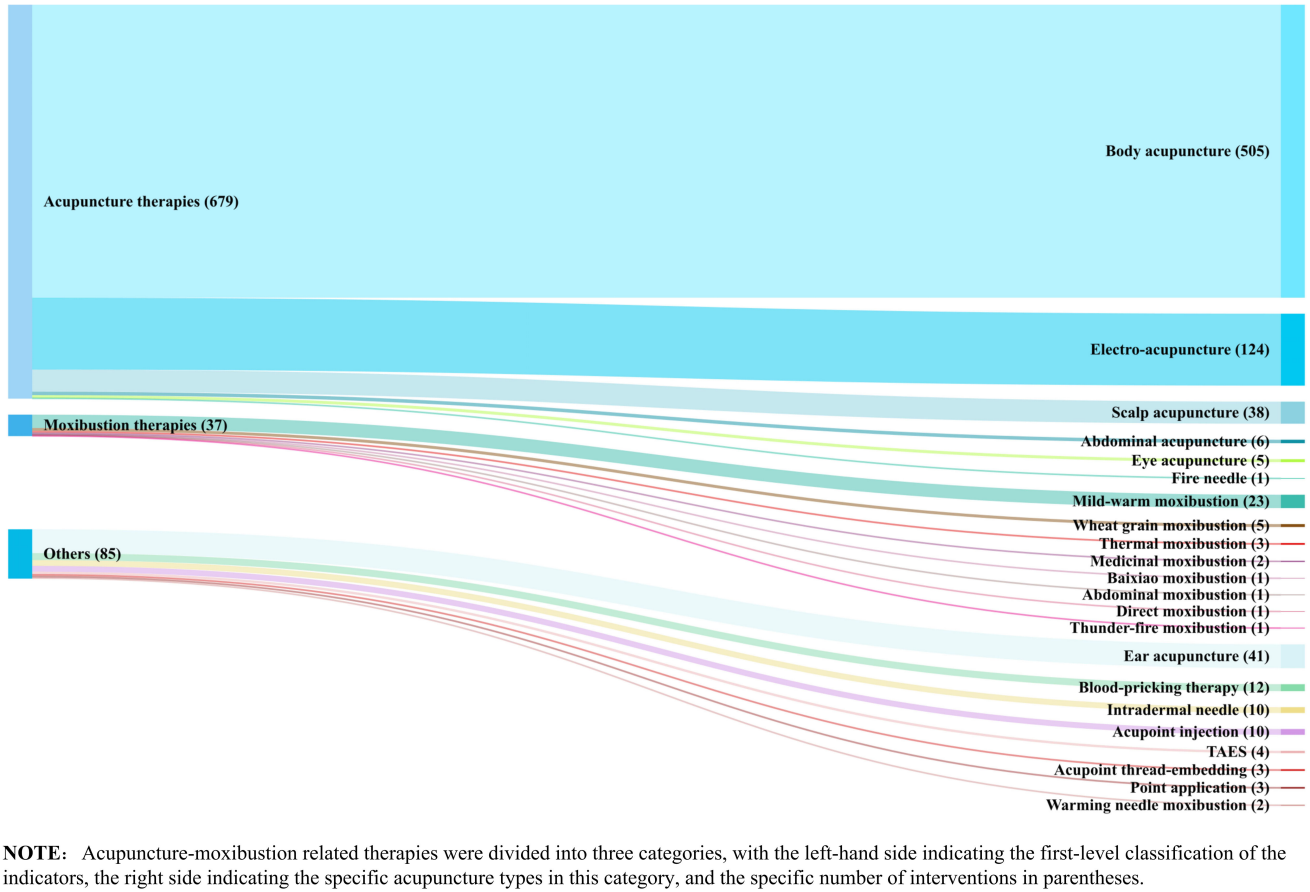
Distribution map of intervention measures categories in RCTs.

**Figure 6** illustrates the interventions used in the RCT studies. Acupuncture was the most widely used therapy (679,84.8%), among which body acupuncture, head acupuncture and electroacupuncture were the most frequently used. Mild-warm moxibustion is the most frequently used research method in moxibustion. In addition, Ear acupuncture and blood-pricking needling therapy have also been well applied.

The intervention analysis of 43 original clinical studies other than RCTs showed that body acupuncture was the most studied intervention object (25,69%), followed by electroacupuncture and moxibustion. At the same time, the combined use of different acupuncture methods, such as the combination of ear acupuncture and acupoint application, and the synergy of hand acupuncture and moxibustion, has also been used in the research. (See **Supplementary Appendix 4**).

The intervention analysis of 43 original clinical studies other than RCTs showed that body acupuncture was the most studied intervention object (25,69%), followed by electroacupuncture and moxibustion. At the same time, the combined use of different acupuncture methods, such as the combination of ear acupuncture and acupoint application, and the synergy of hand acupuncture and moxibustion, has also been used in the research. (See **Supplementary Appendix 4**).

#### 3.3.3 Acupuncture and moxibustion prescription

The information of meridians, acupoints, body parts and specific acupoints appeared in 666 original clinical studies was extracted, of which 9 studies did not mention definite acupoints. Finally, 657 acupuncture prescriptions were extracted, involving 287 acupoints, including some unofficial acupoints.

In this study, the frequency of acupoints was summarized, and the correlation network was drawn. From the point of use frequency, the top 10 acupoints of use frequency were Baihui (GV20), Neiguan (PC6), Taichong (LR3), Sishencong (EX-HN1), Shenmen (HT7), Shenting (GV24), Yingtang (EX-HN3), Sanyinjiao (SP6), Hegu (LI4), Zusanli (ST36). The top 10 meridians were Governor Meridian (DU), The Extra Ordinary Points, Bladder meridian (BL), Pericardium meridian (PC), Liver meridian (LR), Large Intestinal meridian (LI), Stomach meridian (ST), Spleen meridian (SP), Heart meridian (HT), and Gallbladder meridian (GB). Various parts of the body including Head, Forethigh, Foot, Lower leg, Back, Hand were considered high-frequency areas. The high-frequency use of specific acupoints with special therapeutic effects were Yuan primary point, Shu-stream point, Luo-connecting point, Eight confluence points, and Back-shu point, respectively (**Supplementary Appendix 5**).

IBM SPSS Modeller 18.0 software was used to analyze the association rules of the above four parts and make a network diagram. The results were presented in **Table 1**. The thickness and depth of the line in the figure represent the connection strength between the parts. GV20 and PC6 had the highest correlation, indicating that the frequency of their co-occurrence was the highest. Similarly, DU and The Extra Ordinary Points, were considered the most connected meridian. Forethigh-head and Shu-stream point-Yuan primary point were considered to be the most relevant acupoint and specific acupoint, respectively.

**Table 1:** The high-frequency links in acupuncture and moxibustion prescription.

Content	High-frequency Link	Frequency	Network diagram
Acupoint	GV20 — PC6	234	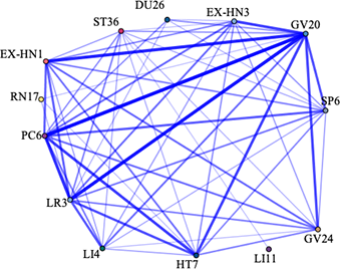
	GV20—LR3	221	
	EX-HN1—GV20	208	
	LR3—PC6	194	
	HT7—PC6	179	
Meridian	DU—The Extra Ordinary Points	357	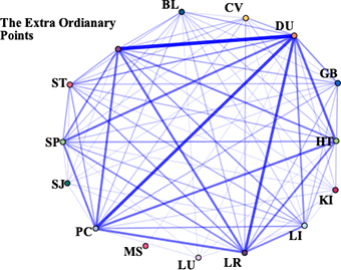
	DU—PC	291	
	DU—LR	266	
	DU—HT	225	
	PC—The Extra Ordinary Points	210	
Body parts	Forethigh—Head	358	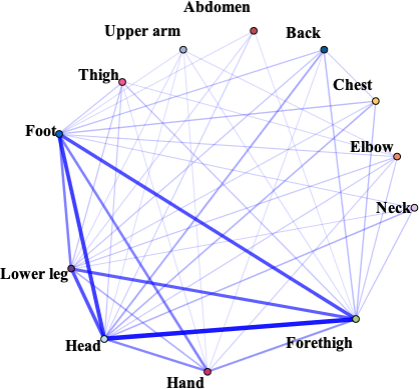
	Foot—Head	297	
	Foot—Forethigh	263	
	Head—Lower leg	260	
	Forethigh—Lower leg	236	
Specific points	Shu-stream point—Yuan primary point	367	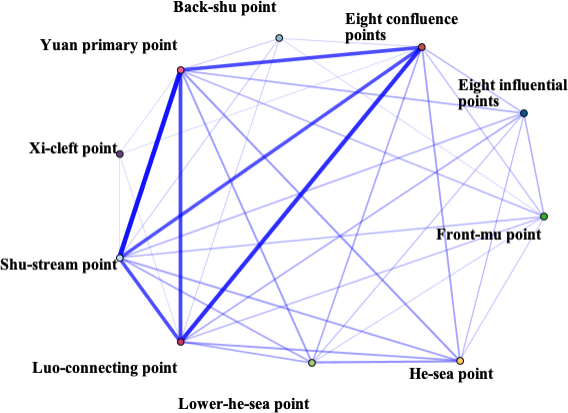
	Eight confluence points—Luo-connecting point	325	
	Luo-connecting point—Yuan primary point	279	
	Eight confluence points—Yuan primary point	272	
	Luo-connecting point—Shu-stream point	268	

#### 3.3.4 Evidence characteristics of Intervention duration

Evidence showed that of 666 clinical studies, 18 did not specify the duration of intervention. The treatment time was mainly concentrated in 2-4 weeks and 4-6 weeks. This may be related to the characteristics of the acupuncture intervention and the primary disease of stroke, as longer intervention duration may cause higher drop-out rate for clinical research. It can also be more challenging for patients and their families. The longest intervention period was up to 5 months. In addition, it is worth noting that only 5 studies were followed up to determine the long-term effects of acupuncture intervention(**Supplementary Appendix 6**).

#### 3.3.5 Evidence characteristics of outcome indicators

The outcome indicators of clinical studies on acupuncture related interventions for PSD were classified and analyzed, mainly using the efficacy evaluation: (1) Clinical efficacy; (2) Major clinical symptoms, including HAMD, SDS, SAS, SERS; (3) associated symptoms, including motor dysfunction, cognitive impairment, sleep disorders; (4) assessment of overall health status, mainly quality of life; (5) Objective examination indicators, including electrocardiogram, electroencephalogram, inflammatory factors, immune function, ultrasound imaging; (6) health economic evaluation, including length of stay and cost; (7) Safety evaluation, including adverse drug reactions and acupuncture adverse events evaluation; (8) TCM symptom score; And (9) others, such as treatment satisfaction, disease prognosis risk, compliance, etc.

**Figure 7:**
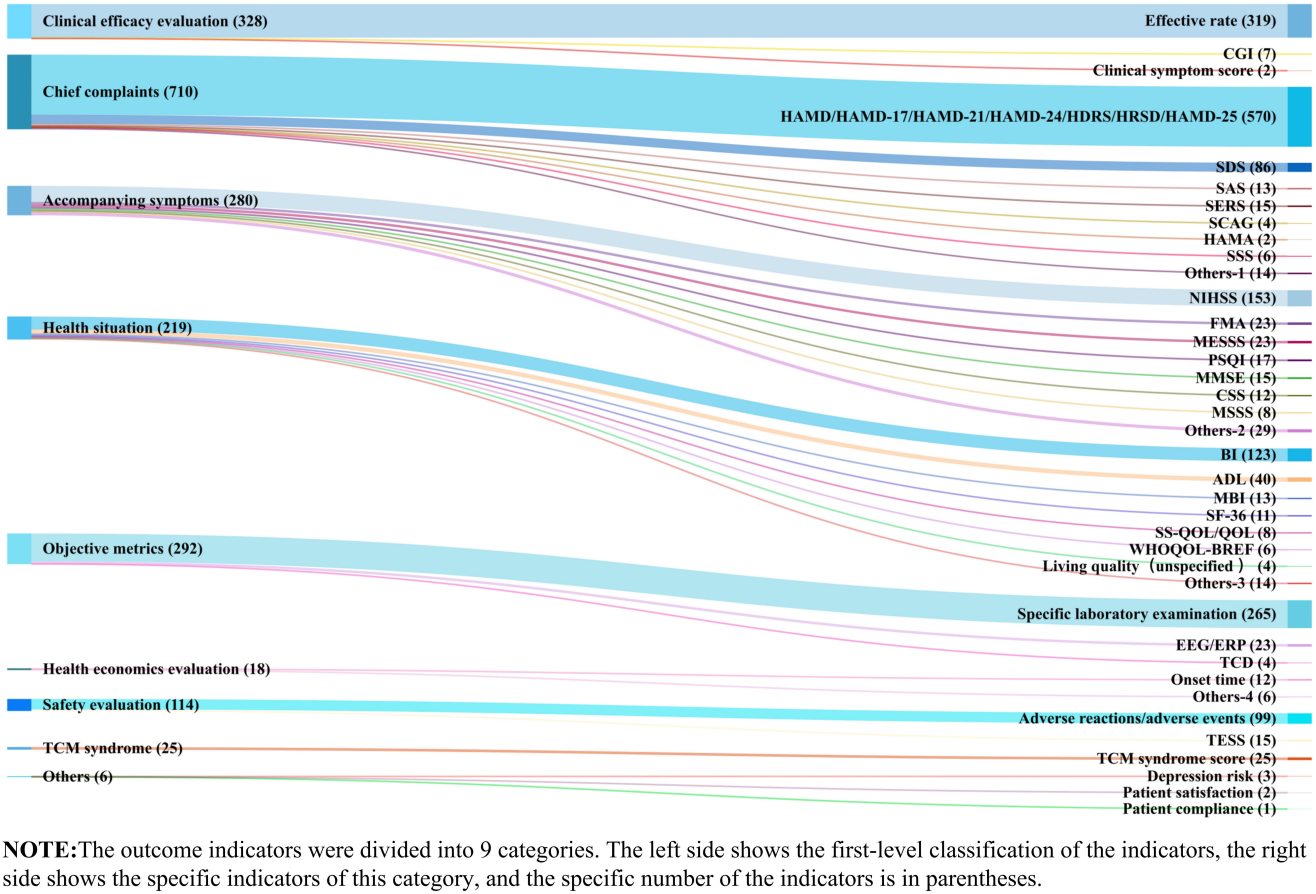
Evidence Characteristics of Outcome indicators.

As shown in **Figure 7**, studies mainly focus on the improvement of the degree of depression, which is mainly evaluated by subjective scales. Although the HAMD is divided into many versions, but most studies do not elaborate on the version, which affects the rigor of the study to a certain extent. Secondly, most of the studies described the clinical efficacy of acupuncture intervention by the effective rate, which was mainly calculated based on the reduction rate of HAMD. Besides, neurological recovery and quality of life were also key points of interest. In terms of objective indicators, research mainly paid attention to hematological indicators and electroencephalogram. It should be noted that acupuncture and moxibustion therapy as a characteristic treatment method under the guidance of traditional Chinese medicine theory, there are few indicators related to the improvement of TCM symptoms in the outcome indicators, indicating that the evidence gap needs to be filled.

### 3.4 Methodological quality assessment

About 45.6% of the 623 randomized controlled trials were classified as low risk of bias by using reasonable randomization methods, such as using random number table and computer software. Some studies used unreasonable random methods such as admission order and hospital of patients, and were rated as having high risk of bias. Only 16 articles used the envelope concealment scheme, which was considered to have a low risk of bias. The remainder did not describe any concealment scheme. Due to the particularity of acupuncture and moxibustion intervention, the blinded evaluation is mostly at high risk of bias, and the integrity of the data in the study is generally satisfactory. All studies reported outcome assessment measures listed in the study methods, but other potential biases were not explicitly mentioned (**Supplementary Appendix 7**). The vast majority of studies did not have a protocol in advance, so selective reporting was not known and was rated as unknown risk.

The quality of 34 SRs/MAs was evaluated. All studies described the types of included literatures, population, intervention, control, and outcome indicators, but lacked the description of treatment course, drug dosage, and location. Only 6 articles had a pre-protocol and were registered. Although the search strategy was explained in all the SRs/MAs, there was a lack of retrieval of grey literature. The literature screening and data extraction were clearly reported. The reasons for the exclusion of each literature were not stated in any of the systematic reviews. 2 studies did not use appropriate tools to assess the risk of bias in each included study, and none of the studies reported the funding sources of each study.2 studies did not use reasonable statistical methods, and about half of the studies considered heterogeneity, but the reasonable explanation and discussion were not enough. Conflicts of interest were noted in 9 studies, but none of the Chinese articles mentioned this. The specific evaluation is provided in the **Supplementary Appendix 8**.

### 3.5 Comprehensive evidence presentation

**Figure 8:**
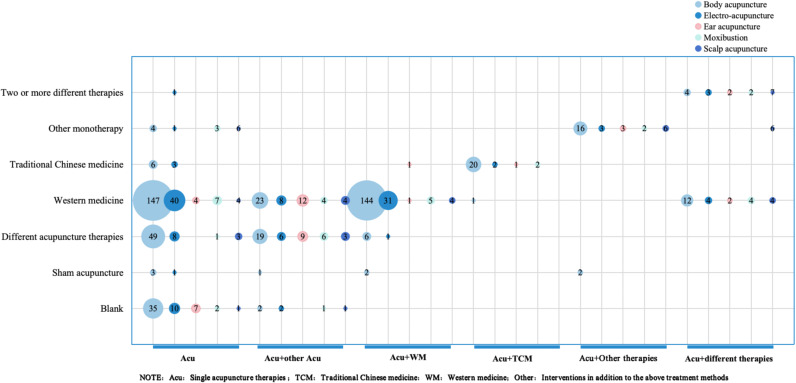
Bubble plot of clinical studies.

The bubble diagram presented in **Figure 8** shows that most RCTs of acupuncture for PSD use single acupuncture as the main intervention. As the most common control method, western medicine is widely used in research. The combined use of multiple acupuncture methods and its combination with western medicine are also the issues of concern in current clinical research.

**Figure 9:**
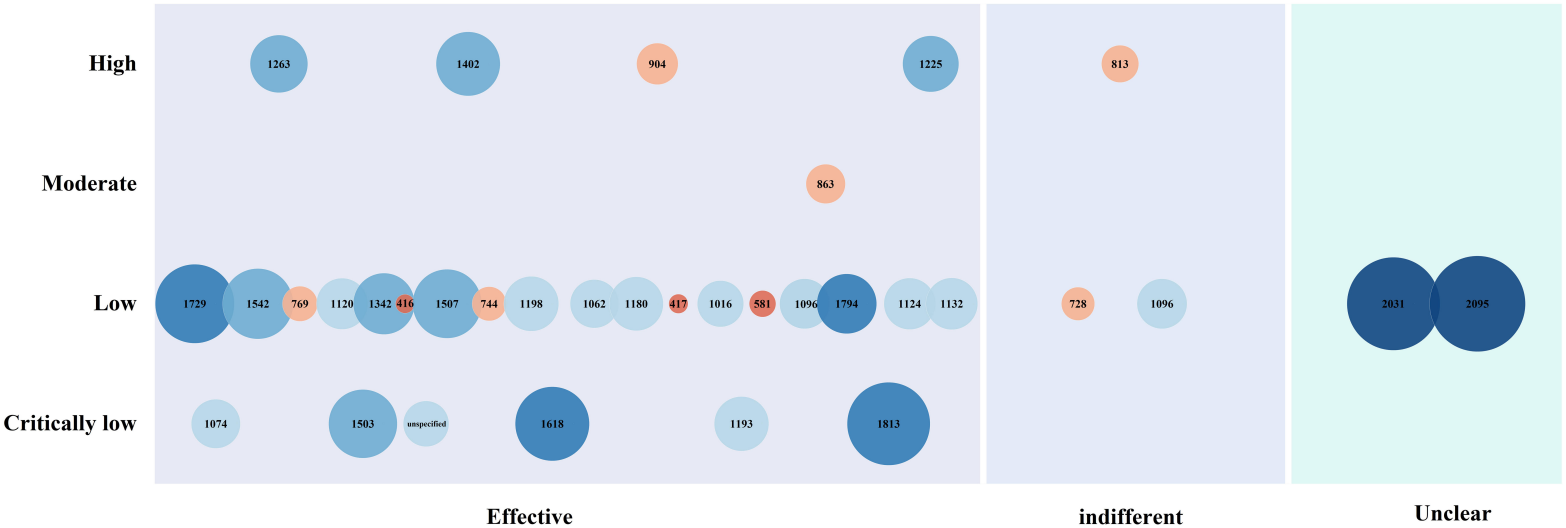
Evidence map of systematic reviews.

The 34 SRs/MAs included in the evidence plot of **Figure 9** are presented as 34 bubbles, and the bubble size represents the sample size included in each studies. The X-axis represents the therapeutic effect of acupuncture intervention on PSD obtained from studies, and the Y-axis reflects the quality of relevant literature. Overall, acupuncture and moxibustion is effective in the treatment of PSD, but the quality of these studies was assessed as poor or low. Therefore, more high-quality studies are needed in the future.

## 4 Discussion

We collected 700 clinical evidences on acupuncture related therapies for PSD, including 666 primary clinical studies and 34 SRs/MAs. The trend of publications showed a fluctuating growth, and the growth rate nearly doubled in the past decade (26, 27), The first systematic review of acupuncture for PSD was published in 2009 (28). Relevant studies were mainly from China, and 89% of the evidence came from RCT studies. The sample size of studies was mostly 30-90. The controls of “acupuncture plus western medicine versus antidepressant” and “acupuncture versus antidepressant” were used the most in the study. GV20 and DU are the most frequently used acupoints and meridians. The intervention period of the whole study was mostly in 2-4 weeks, and there was a lack of follow-up. The HAMD was the main outcome measure to evaluate the improvement of clinical symptoms and effective rate. The overall evidence from clinical studies and systematic reviews suggests that acupuncture has advantages in improving PSD, but most of the them are of low quality. Based on the current evidence we found, our main observations were as follows:

### 4.1 Regional cooperation needs to be strengthened

Our evidence suggested that the studies were mainly completed in China, and most of them were single-center studies, and there was a lack of multi-center RCT trials, which was one of the sources of research bias. Although we included all types of clinical studies, randomized, controlled trials (RCTS) are still considered the gold standard for clinical trial design because they provide the clearest evidence of the effect of an exposure or intervention on outcomes (29). However, to complete a high-quality RCT, it is necessary to identify and eliminate all factors that affect the outcome other than the research purpose, which has many difficulties in practical operation and medical ethics. Studies have shown that case series and case reports also play a certain role in evidence-based medicine, which can help us to better judge the status of research (30). Therefore, it is necessary to carry out multi-center high-quality research and strengthen regional cooperation in the future research on PSD to minimize regional and selection bias. At the same time, when the research funds or implementation conditions are not enough, other types of research can also be selected on the basis of rigorous design to obtain reliable research results.

### 4.2 The study protocol design should be improved

The duration of stroke can affect the symptoms and severity of PSD (31). A study found that PSD mainly occurred after an ischemic stroke and peaked between 3 and 6 months after ischemia (32), while most of the clinical studies in this study did not distinguish between ischemic and hemorrhagic stroke and did not provide a detailed description of the course of disease. In addition, there is no unified standard to accurately distinguish the severity of depression, and there is no authoritative guidelines/diagnostic criteria for the diagnosis of PSD, which makes the study design flawed. Studies have shown that the occurrence of PSD is related to the location of the stroke. Lesions occurring in specific areas such as the prefrontal cortex, limbic regions, and basal ganglia can disrupt key pathways of motion-related neurotransmitters, so stroke patients in this area are more likely to suffer from depression (33–36). However, this evidence showed that few clinical studies described the location of stroke in PSD patients, which could not provide better help for the treatment of PSD with acupuncture, especially in the use of scalp acupuncture. Sample size estimation is related to the reliability, reproducibility and efficiency of research results (37–39). Due to the complexity of researchers, resources, and regional differences, the sample sizes of the included studies in this evidence map are generally small, and most of them lack sample size estimation. It is unknown whether the test power is reasonable. In the future, the inclusion and exclusion criteria should be strictly formulated according to the latest guidelines, and the formulation of research protocols should be more strict and standardized to achieve high quality of research.

### 4.3 Acupuncture operation needs to be standardized

Each acupuncture operation has its own different operating steps and implementation process. Whether it is appropriate to choose and operate it is not only directly related to medical safety, but also directly affects the therapeutic effect of the disease (40). A network meta-analysis of 12 acupuncture methods showed that scalp acupuncture plus conventional acupuncture may be the most effective and safe method to improve the condition of patients with PSD (41). Although the course of treatment, time and operation of acupuncture intervention have some subjectivity and flexibility, in order to draw reliable conclusions, researchers need to standardize the study design. The description of acupuncture intervention in current studies is not detailed enough, and even many of them do not describe the operation process, needle depth and manipulation. Moreover, some research treatment records are unknown, which cannot guarantee reliable conclusions from studies. On the other hand, the qualifications and practice years of operators are also different, which may have a certain impact on the clinical efficacy and evaluation. The lack of follow-up may have contributed to the inadequately demonstrated long-term efficacy of acupuncture. It is hoped that more research can answer some core questions of acupuncture treatment for stroke, such as the choice of acupuncture method, which frequency and course of treatment, and which index can best reflect the efficacy of acupuncture.

### 4.4 Subjective and objective indicators should be combined

Outcome indicators are extremely important to measure the effectiveness of interventions. The objectivity and rationality of their application are not only closely related to efficacy evaluation, but also affect clinical decision-making. PSD is regarded as a psychiatric disorder rather than a neurological disorder. It is mostly evaluated by scales, including self-rating scales, clinician-rating scales, and depression assessment scales for specific populations. Due to the lack of uniform criteria, the validity of these scales varies greatly, leading to differences in efficacy assessments (42). Our study suggests that effective rate is a priority in the clinical evaluation of PSD, but there are differences in its definition or criteria among different studies. At the same time, HAMD is widely used as the primary outcome measure, but the efficacy of different versions is different, and HAMD does not have high validity in all age groups. The study suggests that PHQ-9 has good reliability and validity (43). Meta-analysis showed that The PHQ-9 had a higher diagnostic efficiency for any Depression and the acute phase after stroke, while The Hamilton Rating Scale for Depression performed better for major depression and the chronic phase after stroke (42). Therefore, it is necessary to select relevant scales strictly according to the study population. Currently, there are specific scales for PSD, such as the Post-Stroke Depression Scale (PSDS) (44) and the Post-Stroke Depression Prediction Scale (DePreS) (45). It may also be considered as an outcome measure in future studies. However, as an outcome indicator, the subjective scale has poor accuracy and sensitivity, so it cannot well reflect the real effect of acupuncture treatment. Therefore, the combination of subjective and objective indicators can more accurately reflect the efficacy of acupuncture. A prospective study suggested that higher levels of systemic immune inflammation index (SII), neutrophil-to-lymphocyte (NLR), platelet-to-lymphocyte (PLR), and derived neutrophil-to-lymphocyte ratio (dNLR) were associated with increased prevalence of PSD, especially SII (46). In addition, BDNF is a valuable predictive biomarker and potential therapeutic target for PSD, which can be used to evaluate the efficacy of acupuncture (47) (48). On the other hand, our evidence also suggests that few studies have focused on the side effects of antidepressants in clinical trials, although some studies have suggested that the risk-benefit profile should be considered when deciding whether to prescribe antidepressants (49). In the future, the selection of outcome indicators needs to be comprehensively considered based on the included population, index efficacy and research purpose, and the subjective and objective indicators should be combined for a comprehensive evaluation. In addition, no imaging studies on acupuncture intervention for PSD were found in our evidence, which is a research gap that can be filled in future studies.

### 4.5 High-quality studies are still scarce

All of the examined studies cited randomization, but half of the RCTS did not specify a specific randomization method. Of the studies describing randomization methods, the vast majority did not report the rationale for their sample size determination. They also omitted to describe aspects such as allocation concealment, blinding Settings, etc. This design flaw may introduce bias that may affect the reliability of the findings. Therefore, future research efforts should favor more extensive double-blind randomized controlled trials with extended follow-up. Adherence to appropriate trial design will help to provide a large amount of evidence-based information for clinical practice. The methodological quality of the included studies was low (using inadequately designed statistical methods) and the quality of the studies was low. Some articles did not specifically describe the method of randomization, and most of them did not mention allocation concealment. Most of them do not mention the blinding method and the particularity of acupuncture research, so it is difficult to achieve double blinding in clinical trials. Incomplete or inconsistent baseline information, scarcity of original data; There was a potential publication bias, which may lead to low evidence quality, overreporting of positive results and underreporting of adverse events. Most of the literature did not describe the drop-out of patients and loss of follow-up/withdrawal and how to deal with it. Inadequate description of statistical methods; Most studies did not provide adequate, complete information. The quality of meta-analysis depends on the quality of the RCT included in the meta-analysis and its control of bias. Low-quality RCT studies will reduce the authenticity of the results and make the conclusions unreliable.

## 5 Limitations

This study is the first to use evidence maps to clarify and present the current status of clinical literature as well as systematic reviews/meta-analyses and clinical research on acupuncture treatment for PSD. The findings suggest that acupuncture has some advantages in the treatment of PSD. However, there is room for improvement in our study.

Notwithstanding the rigorous screening of the eight databases, given that acupuncture is an external therapeutic modality of Traditional Chinese Medicine (TCM), the preponderance of research sources is predominantly from China. This geographical bias in research origin is the underlying cause of the relatively singular study types and evidence composition. In future research endeavors, it is recommended to incorporate a more diverse range of databases. In this study, our analytical scope was circumscribed to the interventions alone did not assess the duration or frequency of treatment for each intervention or the specific names and dosages of comparator medications. These methodological constraints are liable to exert an influence on the external validity and translational utility of the study findings.

## 6 Conclusions

With the development of economy and society, the number of stroke patients is increasing and the incidence of PSD is also increasing. Acupuncture has obvious advantages in the prevention and treatment of PSD. In order to better transform the evidence, future studies need to formulate strict inclusion and exclusion criteria, standardize acupuncture operation process, select reasonable outcome indicators, conduct more rigorous design and higher quality clinical trials, and conduct higher quality SRs/MAs.

## Data Availability

The original contributions presented in the study are included in the article/**Supplementary Material**. Further inquiries can be directed to the corresponding author.
