# Supplementary material for: Acupuncture therapies for post-stroke depression: the evidence mapping of clinical studies
Source: Front Psychiatry. 2025 Mar 4;16:1523050. doi: 10.3389/fpsyt.2025.1523050 (PMC11914146; doi:10.3389/fpsyt.2025.1523050)
Supplement: Supplementary file 1 [file Supplementaryfile1.pdf]

Supplementary Material

Appendix 1 Search strategy in selective databases.

Appendix 2 The list of included studies

Appendix 3.Distribution and situation of countries and regions

Appendix 4 Overview of other original study interventions

Appendix 5 Frequency statistics of acupoint related information

Appendix 6 Intervention duration of clinical studies

Appendix 7 Assessment of risk of bias in clinical randomized controlled trials

Appendix 8 Quality assessment of SRs/MAs

Appendix 1. Search strategy in selective databases.

Database	Search Term	Results
PubMed	#1: (((((((((((((((((((((((((((((((((((((Stroke, Acute[Title/Abstract]) OR (Brain Infarction[Title/Abstract])) OR (Cerebral Infarct[Title/Abstract])) OR (Intracerebral Hemorrhage[Title/Abstract])) OR (Cerebral Hemorrhage[Title/Abstract])) OR (Cerebral Brain Hemorrhage[Title/Abstract])) OR (Hemorrhagic Stroke[Title/Abstract])) OR (Hypertensive Intracerebral Hemorrhage[Title/Abstract])) OR (Hemorrhage, Cerebrum[Title/Abstract])) OR (Cerebrum Hemorrhage[Title/Abstract])) OR (Cerebral Parenchymal Hemorrhage[Title/Abstract])) OR (Hemorrhage, Cerebral Parenchymal[Title/Abstract])) OR (Parenchymal Hemorrhage, Cerebral[Title/Abstract])) OR (Hemorrhage, Intracerebral[Title/Abstract])) OR (Hemorrhage, Cerebral[Title/Abstract])) OR (Cerebral Hemorrhage[Title/Abstract])) OR (Brain Hemorrhage, Cerebral[Title/Abstract])) OR (Hemorrhage, Cerebral Brain[Title/Abstract])) OR (Cerebrovascular Accident[Title/Abstract])) OR (Acute Cerebrovascular Accident[Title/Abstract])) OR (CVA[Title/Abstract])) OR (Vascular Accident, Brain[Title/Abstract])) OR (Brain Vascular Accident[Title/Abstract])) OR (Stroke, Cerebral[Title/Abstract])) OR (Stroke, Cerebrovascular[Title/Abstract])) OR (Brain Ischemia[Title/Abstract])) OR (Thrombotic Stroke[Title/Abstract])) OR (Ischemic Stroke[Title/Abstract])) OR (Wake up Stroke[Title/Abstract])) OR (Embolic Stroke[Title/Abstract])) OR (Acute Stroke[Title/Abstract])) OR (Cerebral Stroke[Title/Abstract])) OR (Cerebrovascular Stroke[Title/Abstract])) OR (Apoplexy, Cerebrovascular[Title/Abstract])) OR (Cerebrovascular Apoplexy[Title/Abstract])) OR (Apoplexy[Title/Abstract])) OR (Stroke[Title/Abstract])) OR (Stroke[MeSH Terms])<br><br>#2: (((((Depression[MeSH Terms]) OR (Depressed[Title/Abstract])) OR (Depressive Symptoms[Title/Abstract])) OR (Depressive disorder[Title/Abstract])) OR (Emotional Depression[Title/Abstract])) OR (Mood Disorder[Title/Abstract])<br><br>#3: ((Acupuncture[MeSH Terms]) OR (Moxibustion[MeSH Terms])) OR (Acupuncture Therapy[MeSH Terms])<br><br>#4:((Acupuncture[Title/Abstract]) OR (Acupuncture Therapy[Title/Abstract])) OR (Acupuncture Treatment[Title/Abstract])) OR (Treatment, Acupuncture[Title/Abstract])) OR (Therapy, Acupuncture[Title/Abstract])) OR (Manual Acupuncture[Title/Abstract])) OR (Acupuncture-moxibustion[Title/Abstract])) OR (Moxibustion[Title/Abstract])) OR (Moxabustion[Title/Abstract])) OR (Warm needle[Title/Abstract])) OR (Warm Acupuncture[Title/Abstract])) OR (Thermoacupuncture[Title/Abstract])) OR (Electroacupuncture[Title/Abstract])) OR (Electro-acupuncture[Title/Abstract])) OR (electric acupuncture[Title/Abstract])) OR (Acupuncture Point[Title/Abstract])) OR (Point, Acupuncture[Title/Abstract])) OR (Acupoint[Title/Abstract])) OR (Pharmacopuncture[Title/Abstract])) OR (Pharmacoacupuncture Treatment[Title/Abstract])) OR (Treatment,	97

	Pharmacoacupuncture[Title/Abstract])) OR (Pharmacoacupuncture Therapy[Title/Abstract])) OR (Therapy, Pharmacoacupuncture[Title/Abstract])) OR (Acupoint injection[Title/Abstract])) OR (acupuncture injection[Title/Abstract])) OR (pharmaco-acupuncture[Title/Abstract])) OR (auricular needle[Title/Abstract])) OR (Acupunctures, Ear[Title/Abstract])) OR (Ear Acupuncture[Title/Abstract])) OR (Ear needle[Title/Abstract])) OR (earlobe acupuncture[Title/Abstract])) OR (Auricular Acupuncture[Title/Abstract])) OR (Auriculotherapy[Title/Abstract])) OR (Acupuncture, Auricular[Title/Abstract])) OR (auricular plaster therapy[Title/Abstract])) OR (auricular point sticking[Title/Abstract])) OR (Auricular pressure[Title/Abstract])) OR (Fire needle[Title/Abstract])) OR (Fire acupuncture[Title/Abstract])) OR (Acupoint catgut embedding[Title/Abstract])) OR (Scalp acupuncture[Title/Abstract])) OR (Scalp needle[Title/Abstract])) OR (Scalp electroacupuncture[Title/Abstract])) OR (scalp stimulation[Title/Abstract])) OR (Eye needle[Title/Abstract])) OR (Eye acupuncture[Title/Abstract])) OR (Abdominal acupuncture[Title/Abstract])) OR (Abdominal acupuncture[Title/Abstract])) OR (filiform needle[Title/Abstract])) OR (silver needle[Title/Abstract])) OR (three-edged needle[Title/Abstract])) OR (intradermal needle[Title/Abstract])) OR (Point application[Title/Abstract])) OR (needle-embedding[Title/Abstract])) OR (Catgut Embedding[Title/Abstract])) OR (pricking therapy[Title/Abstract])) OR (point injection[Title/Abstract])) OR (Skin Acupuncture[Title/Abstract])) OR (transcutanclus electrical acupoint stimulation[Title/Abstract])) OR (TEAS[Title/Abstract])) OR (electrical acupoint stimulation[Title/Abstract])) OR (Acupuncture Point[Title/Abstract])) OR (Acupoint[Title/Abstract])<br>#5:(#3) OR (#4)<br>#6: ((#1) AND (#3)) AND (#5)	
Web of Science	((TS=(Stroke OR "Acute Stroke" OR "Ischemic Stroke" OR "Hemorrhagic Stroke" OR "Cerebral Stroke" OR "Cerebrovascular Stroke" OR "Cerebrovascular Accident" OR "Acute Cerebrovascular Accident" OR Apoplexy OR "Cerebrovascular Apoplexy" OR "Vascular Accident" OR "CVA" OR "Brain Vascular Accident" OR "Post-stroke")) AND TS=(Depression OR "Depressive Symptoms" OR "Depressive disorder" OR "Emotional Depression" OR "Mood Disorder")) AND TS=(Acupuncture OR Acupuncture Therapy OR Acupuncture Treatment OR Treatment, Acupuncture OR Therapy, Acupuncture OR Manual Acupuncture OR Acupuncture-moxibustion OR Moxibustion OR Moxabustion OR Warm needle OR Warm Acupuncture OR Thermoacupuncture OR Electroacupuncture OR Electro-acupuncture OR electric acupuncture OR Acupuncture Point OR Point, Acupuncture OR Acupoint OR Pharmacopuncture OR Pharmacoacupuncture Treatment OR Treatment, Pharmacoacupuncture OR Pharmacoacupuncture Therapy OR Therapy, Pharmacoacupuncture OR Acupoint injection OR acupuncture injection OR pharmaco-acupuncture OR auricular needle OR Acupunctures, Ear OR Ear Acupuncture OR Ear needle OR earlobe acupuncture OR Auricular Acupuncture OR Auriculotherapy OR Acupuncture, Auricular OR auricular plaster therapy OR auricular point sticking OR Auricular pressure OR Fire needle OR Fire acupuncture OR Acupoint catgut embedding OR Scalp acupuncture OR Scalp needle OR Scalp electroacupuncture OR scalp stimulation OR Eye needle OR Eye acupuncture OR Abdominal acupuncture OR Abdominal needle OR filiform needle OR silver needle OR three-edged needle OR intradermal	320

	needle OR Point application OR needle-embedding OR Catgut Embedding OR pricking therapy OR point injection OR Skin Acupuncture OR transcutanclus electrical acupoint stimulation OR TEAS OR electrical acupoint stimulation OR Acupuncture Point OR Acupoint)	
Embase	#1:stroke:ti,ab,kw OR 'acute stroke':ti,ab,kw OR 'ischemic stroke':ti,ab,kw OR 'hemorrhagic stroke':ti,ab,kw OR 'cerebral stroke':ti,ab,kw OR 'cerebrovascular stroke':ti,ab,kw OR 'cerebrovascular accident':ti,ab,kw OR 'acute cerebrovascular accident':ti,ab,kw OR apoplexy:ti,ab,kw OR 'cerebrovascular apoplexy':ti,ab,kw OR 'vascular accident':ti,ab,kw OR 'cva':ti,ab,kw OR 'brain vascular accident':ti,ab,kw OR 'post-stroke':ti,ab,kw<br>#2:depression:ti,ab,kw OR 'depressive symptoms':ti,ab,kw OR 'depressive disorder':ti,ab,kw OR 'emotional depression':ti,ab,kw OR 'mood disorder':ti,ab,kw<br>#3: acupuncture:ti,ab,kw OR 'acupuncture therapy':ti,ab,kw OR 'acupuncture treatment':ti,ab,kw OR 'treatment, acupuncture':ti,ab,kw OR 'therapy, acupuncture':ti,ab,kw OR 'manual acupuncture':ti,ab,kw OR 'acupuncture moxibustion':ti,ab,kw OR moxibustion:ti,ab,kw OR moxabustion:ti,ab,kw OR 'warm needle':ti,ab,kw OR 'warm acupuncture':ti,ab,kw OR thermoacupuncture:ti,ab,kw OR electroacupuncture:ti,ab,kw OR 'electro acupuncture':ti,ab,kw OR 'electric acupuncture':ti,ab,kw OR 'point, acupuncture':ti,ab,kw OR pharmacopuncture:ti,ab,kw OR 'pharmacoacupuncture treatment':ti,ab,kw OR 'treatment, pharmacoacupuncture':ti,ab,kw OR 'pharmacoacupuncture therapy':ti,ab,kw OR 'therapy, pharmacoacupuncture':ti,ab,kw OR 'acupoint injection':ti,ab,kw OR 'acupuncture injection':ti,ab,kw OR 'pharmaco acupuncture':ti,ab,kw OR 'auricular needle':ti,ab,kw OR 'acupunctures, ear':ti,ab,kw OR 'ear acupuncture':ti,ab,kw OR 'ear needle':ti,ab,kw OR 'earlobe acupuncture':ti,ab,kw OR 'auricular acupuncture':ti,ab,kw OR auriculotherapy:ti,ab,kw OR 'acupuncture, auricular':ti,ab,kw OR 'auricular plaster therapy':ti,ab,kw OR 'auricular point sticking':ti,ab,kw OR 'auricular pressure':ti,ab,kw OR 'fire needle':ti,ab,kw OR 'fire acupuncture':ti,ab,kw OR 'acupoint catgut embedding':ti,ab,kw OR 'scalp acupuncture':ti,ab,kw OR 'scalp needle':ti,ab,kw OR 'scalp electroacupuncture':ti,ab,kw OR 'scalp stimulation':ti,ab,kw OR 'eye needle':ti,ab,kw OR 'eye acupuncture':ti,ab,kw OR 'abdominal acupuncture':ti,ab,kw OR 'abdominal needle':ti,ab,kw OR 'filiform needle':ti,ab,kw OR 'silver needle':ti,ab,kw OR 'three-edged needle':ti,ab,kw OR 'intradermal needle':ti,ab,kw OR 'point application':ti,ab,kw OR 'needle embedding':ti,ab,kw OR 'catgut embedding':ti,ab,kw OR 'pricking therapy':ti,ab,kw OR 'point injection':ti,ab,kw OR 'skin acupuncture':ti,ab,kw OR 'transcutanclus electrical acupoint stimulation':ti,ab,kw OR teas:ti,ab,kw OR 'electrical acupoint stimulation':ti,ab,kw OR 'acupuncture point':ti,ab,kw OR acupoint:ti,ab,kw<br>#4: #1AND #2 AND #3	185
Cochrane Library	#1:(Stroke OR "Acute Stroke" OR "Ischemic Stroke" OR "Hemorrhagic Stroke" OR "Cerebral Stroke" OR "Cerebrovascular Stroke" OR "Cerebrovascular Accident" OR "Acute Cerebrovascular Accident" OR Apoplexy OR "Cerebrovascular Apoplexy" OR "Vascular Accident" OR "CVA" OR "Brain Vascular Accident" OR "Post-stroke"):ti,ab,kw<br>#2: (Depression OR "Depressive Symptoms" OR "Depressive disorder" OR "Emotional Depression" OR "Mood Disorder"):ti,ab,kw	341

	#3:(Acupuncture OR Acupuncture Therapy OR Acupuncture Treatment OR Treatment, Acupuncture OR Therapy, Acupuncture OR Manual Acupuncture OR Acupuncture-moxibustion OR Moxibustion OR Moxabustion OR Warm needle OR Warm Acupuncture OR Thermoacupuncture OR Electroacupuncture OR Electro-acupuncture OR electric acupuncture OR Acupuncture Point OR Point, Acupuncture OR Acupoint OR Pharmacopuncture OR Pharmacoacupuncture Treatment OR Treatment, Pharmacoacupuncture OR Pharmacoacupuncture Therapy OR Therapy, Pharmacoacupuncture OR Acupoint injection OR acupuncture injection OR pharmaco-acupuncture OR auricular needle OR Acupunctures, Ear OR Ear Acupuncture OR Ear needle OR earlobe acupuncture OR Auricular Acupuncture OR Auriculotherapy OR Acupuncture, Auricular OR auricular plaster therapy OR auricular point sticking OR Auricular pressure OR Fire needle OR Fire acupuncture OR Acupoint catgut embedding OR Scalp acupuncture OR Scalp needle OR Scalp electroacupuncture OR scalp stimulation OR Eye needle OR Eye acupuncture OR Abdominal acupuncture OR Abdominal needle OR filiform needle OR silver needle OR three-edged needle OR intradermal needle OR Point application OR needle-embedding OR Catgut Embedding OR pricking therapy OR point injection OR Skin Acupuncture OR transcutanclus electrical acupoint stimulation OR TEAS OR electrical acupoint stimulation OR Acupuncture Point OR Acupoint):ti,ab,kw<br><br>#4:#1AND #2 AND #3	
CNKI	(SU%='抑郁'+'抑郁状态'+"抑郁症'+'抑郁情绪'+'抑郁症状' AND TKA%='抑郁'+'抑郁状态'+'抑郁症'+'抑郁情绪'+'抑郁症状)AND (SU%='中风'+'卒中'+'脑梗死'+'脑梗塞'+'脑出血' AND TKA%='中风'+'卒中'+"脑梗死'+'脑梗塞'+'脑出血")AND (SU%='针刺'+'电针'+'针灸'+'刺法'+'经皮穴位电刺激'+'隔姜灸'+'隔药灸'+'隔附子饼灸'+'体针'+'耳针'+'头针'+'毫针'+'隔盐灸'+'耳穴贴压'+'热敏灸'+'梅花针'+'穴位敷贴'+"穴位贴敷'+'天灸'+"针刺治疗'+"针灸疗法'+'穴位埋线'+'火针'+'穴位注射'+"艾灸'+'灸法'+'灸疗'+'灸术'+'温针'+"针法'+'三棱针'+'皮肤针'+'芒针'+'眼针'+'手针'+'足针'+'腕踝针'+'平衡针'+"揿针'+"皮内针'+"腹针'+'舌针'+'项针' AND TKA%="针刺'+'电针'+"针灸'+'刺法'+'经皮穴位电刺激'+'隔姜灸'+'隔药灸'+'隔附子饼灸'+'体针'+'耳针'+'头针'+'毫针'+'隔盐灸'+'耳穴贴压'+'热敏灸'+'梅花针'+'穴位敷贴'+'穴位贴敷'+'天灸'+'针刺治疗'+"针灸疗法'+'穴位埋线'+'火针'+'穴位注射'+"艾灸'+'灸法'+'灸疗'+'灸术'+"温针'+"针法'+'三棱针'+'皮肤针'+'芒针'+'眼针'+'手针'+'足针'"+'腕踝针'+'平衡针'+'揿针'+'皮内针'+'腹针'+'舌针'+'项针)	1684
CBM	("针刺"[核心字段:智能] OR "电针"[核心字段:智能] OR "针灸"[核心字段:智能] OR "刺法"[核心字段:智能] OR "经皮穴位电刺激"[核心字段:智能] OR "隔姜灸"[核心字段:智能] OR "隔药灸"[核心字段:智能] OR "隔附子饼灸"[核心字段:智能] OR "体针"[核心字段:智能] OR "耳针"[核心字段:智能] OR "头针"[核心字段:智能] OR "毫针"[核心字段:智能] OR "隔盐灸"[核心字段:智能] OR "耳穴贴压"[核心字段:智能] OR "热敏灸"[核心字段:智能] OR "梅花针"[核心字段:智能] OR "穴位敷贴"[核心字段:智能] OR "穴位贴敷"[核心字段:智能] OR "天灸"[核心字段:智能] OR "针刺治疗"[核心字段:智能] OR "针灸疗法"[核心字段:智能] OR "穴位埋线"[核心字段:智能] OR "火针"[核心字段:智能] OR "穴位注射"[核心字段:智能] OR "艾灸"[核心字段:智能] OR "灸法"[核心字段:智能] OR "灸疗"[核心字段:智能] OR "灸术"[核心字段:智	1369

	能] OR "温针"[核心字段:智能] OR "针法"[核心字段:智能] OR "三棱针"[核心字段:智能] OR "皮肤针"[核心字段:智能] OR "芒针"[核心字段:智能] OR "眼针"[核心字段:智能] OR "手针"[核心字段:智能] OR "足针"[核心字段:智能] OR "腕踝针"[核心字段:智能] OR "平衡针"[核心字段:智能] OR "揿针"[核心字段:智能] OR "皮内针"[核心字段:智能] OR "腹针"[核心字段:智能] OR "舌针"[核心字段:智能] OR "项针"[核心字段:智能]) AND("中风"[核心字段:智能] OR "卒中"[核心字段:智能] OR "脑梗死"[核心字段:智能] OR "脑梗塞"[核心字段:智能] OR "脑出血"[核心字段:智能]) AND("抑郁"[核心字段:智能] OR "抑郁状态"[核心字段:智能] OR "抑郁症"[核心字段:智能] OR "抑郁情绪"[核心字段:智能] OR "抑郁症状"[核心字段:智能])	
VIP	M=(针刺 OR 电针 OR 针灸 OR 刺法 OR 经皮穴位电刺激 OR 隔姜灸 OR 隔药灸 OR 隔附子饼灸 OR 体针 OR 耳针 OR 头针 OR 毫针 OR 隔盐灸 OR 耳穴贴压 OR 热敏灸 OR 梅花针 OR 穴位敷贴 OR 穴位贴敷 OR 天灸 OR 针刺治疗 OR 针灸疗法 OR 穴位埋线 OR 火针 OR 穴位注射 OR 艾灸 OR 灸法 OR 灸疗 OR 灸术 OR 温针 OR 针法 OR 三棱针 OR 皮肤针 OR 芒针 OR 眼针 OR 手针 OR 足针 OR 腕踝针 OR 平衡针 OR 揿针 OR 皮内针 OR 腹针 OR 舌针 OR 项针) AND M=(中风 OR 卒中 OR 脑梗死 OR 脑梗塞 OR 脑出血) AND M=(抑郁 OR 抑郁状态 OR 抑郁症 OR 抑郁情绪 OR 抑郁症状)	1223
Wanfang database	主题:("针刺"OR"电针"OR"针灸"OR"刺法"OR"经皮穴位电刺激"OR"隔姜灸"OR"隔药灸"OR"隔附子饼灸"OR"体针"OR"耳针"OR"头针"OR"毫针"OR"隔盐灸"OR"耳穴贴压"OR"热敏灸"OR"梅花针"OR"穴位敷贴"OR"穴位贴敷"OR"天灸"OR"针刺治疗"OR"针灸疗法"OR"穴位埋线"OR"火针"OR"穴位注射"OR"艾灸"OR"灸法"OR"灸疗"OR"灸术"OR"温针"OR"针法"OR"三棱针"OR"皮肤针"OR"芒针"OR"眼针"OR"手针"OR"足针"OR"腕踝针"OR"平衡针"OR"揿针"OR"皮内针"OR"腹针"OR"舌针"OR"项针") and 主题:("中风"OR "卒中"OR"脑梗死"OR"脑梗塞" OR"脑出血") and 主题:("抑郁"OR"抑郁状态"OR"抑郁症"OR"抑郁情绪"OR"抑郁症状")	1842

Appendix 2. The list of included studies

Clinical trials in Chinese	1. Wang Meiqin, Zhang Jianzhi. Efficacy Analysis of the "Trinity" Therapy in the Treatment of Post - Stroke Depression. Hebei Medical Journal. 2011;33(10):1589 - 1590.<br><br>2. Geng Shuanglin, Shi Zhen. Clinical Effect Analysis of Different Acupuncture Therapies on the Improvement of Post - Stroke Depression Patients. Shanxi Medical Journal. 2020;49(23):3268 - 3270.<br><br>3. Wang Yanwu, Wang Chongmin, Sun Yunting. Observation on the Efficacy of Electroacupuncture with Different Frequencies in the Treatment of Post - Stroke Depression. Shanghai Journal of Acupuncture and Moxibustion. 2015;34(09):27 - 29.<br><br>4. Meng Fanhong, Xing Liling, Zhao Xiaoguang. Influence of Personalized Nursing Model Combined with Auricular Point Bianstone - Magnetic Bead Application on Post - Stroke Depression. Capital Food Medicine. 2022;29(10).<br><br>5. Huang Yanzhu. Exploratory Study on the Application of Traditional Chinese Medicine Scalp Electroacupuncture in Reducing the Incidence of Post - Stroke Depression (PSD). Health Care Guide. 2019;(30):313.<br><br>6. Shi Yanyu, Zheng Kai, Zhao Fuli. Analysis of the Treatment of Post - Stroke Depression by Traditional Chinese Medicine Rehabilitation Acupuncture. Full - text Edition of Medicine and Health in Chinese Science and Technology Journal Database. 2022;(11).<br><br>7. Tang Zenian, Yuan Lingzhong. Analysis of the Treatment of Post - Stroke Depression by Traditional Chinese Medicine Rehabilitation Acupuncture. Da Jia Jian Kang (Academic Edition). 2015;9(04):37.<br><br>8. Wang Ting. Analysis of the Treatment of Post - Stroke Depression by Traditional Chinese Medicine Rehabilitation Acupuncture. Jian Kang Bi Du. 2020;(14):167.<br><br>9. Zheng Qiufeng. Efficacy Observation of Traditional Chinese Medicine Acupuncture in the Treatment of Post - Stroke Depression Patients. Medical Equipment. 2016;29(14):137.<br><br>10. Zhan Jinyu. Observation and Research on the Treatment of Post - Stroke Depression by Acupuncture. Medical Information (Medicine and Computer Applications). 2014;0(25).<br><br>11. Che Yanhua, Wang Yanmei. Efficacy Evaluation of Acupuncture in the Treatment of Post - Stroke Depression. Medicine Frontiers. 2014;(32):338 - 338.<br><br>12. Sun Yuanzheng, Jia Shuya. Clinical Observation on the Treatment of Post - Stroke Depression by Yu's Scalp - Point Cluster Needling. Shanghai Journal of Acupuncture and Moxibustion. 2012;31(08):564 - 565.<br><br>13. Ma Liying, Gong Shufeng, Huo Jin. Clinical Observation on the Treatment of Post - Stroke Depression with Yu's Scalp Acupuncture as the Main Therapy. Journal of Clinical Acupuncture and Moxibustion. 2012;28(05):66 - 68.<br><br>14. He Qianchao, Liu Tai, Lu Yinyan, Gao Yuguang, He Qing, Huang He. Influence of Five - Element Dialectical Acupuncture on the Quality of Life of Post - Stroke Depression Patients. Journal of Clinical Psychosomatic Diseases. 2016;22(1).

15. Liu Tai, Zhou Zhi, Gao Yuguang, et al. Randomized Controlled Clinical Study on the Treatment of Post - Stroke Depression by Five - Element Acupuncture. Journal of Sichuan Traditional Chinese Medicine. 2016;34(10):176 - 178.

16. Wang Yuying, Yuan Aihong, Yang Jun, Pan Bin, Xie Hongyu, Zhang Xueqin. Influence of the Method of Reusing Yuan - Source Points in Five - Element Acupuncture on the Emotions and Sleep of Post - Stroke Depression Patients. Journal of Anhui University of Chinese Medicine. 2023;42(01):49 - 53.

17. Zhang Yao, Sun Jinglong, Lin Chengjie. Observation on the Efficacy of Five - Element Music Therapy Combined with Acupuncture in the Treatment of Post - Stroke Depression. Inner Mongolia Journal of Traditional Chinese Medicine. 2018;37(07):99 - 101.

18. Wu Shengbing, Yang Dan. Efficacy Observation of Pricking and Blood - Letting at Jing - Well Points on Post - Stroke Depression. Liaoning Journal of Traditional Chinese Medicine. 2015;42(1).

19. Yang Dan, Wu Shengbing, Zeng Xian, Yang Mei, Yang Xiaofang. Research on the Influence of Jing - Well Point Blood - Letting Therapy on the Depressive State and Serum 5 - Hydroxytryptamine Level of Post - Stroke Depression Patients. Chinese General Practice. 2016;19(4).

20. Liu Hongwen. Clinical Efficacy of Treating Post - Stroke Depression from the Liver and Spleen. Chinese Medicine Modern Distance Education of China. 2015;13(05):25 - 27.

21. Nie Rongrong, Huang Chunhua, Fu Wenbin. Efficacy Observation of Treating Post - Stroke Depression from the Spleen and Stomach. Chinese Acupuncture & Moxibustion. 2011;31(04):325 - 328.

22. Sun Jianhua, Zhu Shanshan, Pei Lixia. Clinical Observation on 30 Cases of Post - Stroke Depression Treated by Brain - Nourishing and Mind - Regulating Acupuncture. Jiangsu Journal of Traditional Chinese Medicine. 2009;41(11):46 - 47.

23. Zhu Shanshan, Sun Jianhua. Treatment of 30 Cases of Post - Stroke Depression by Brain - Nourishing and Mind - Regulating Acupuncture. Journal of Traditional Chinese Medicine. 2008;(07):653.

24. Zhu Shanshan, Sun Jianhua. Clinical Study on the Treatment of 30 Cases of Post - Stroke Depression by Brain - Nourishing and Mind - Regulating Acupuncture. Journal of Zhejiang Chinese Medical University. 2008;(06):803 - 804.

25. Bao Chao. Clinical Observation on 62 Cases of Post - Stroke Depression Treated by Brain - Nourishing and Mind - Regulating Acupuncture. Shanghai Journal of Acupuncture and Moxibustion. 2005;(10):9 - 10.

26. Luo Junting, Guo Huiming, Guo Min, et al. Therapeutic Effect of Acupuncture with the Method of Invigorating the Spleen and Regulating Qi Combined with Wheat - Grain Moxibustion on Mild - to - Moderate Post - Stroke Depression of Liver - Depression and Spleen - Deficiency Type. Medical Innovation of China. 2021;18(15):78 - 81.

27. Wang Qiuhua, Liu Lanlan. Clinical Study on the Treatment of Post - Stroke Depression by Acupuncture at Six - Spirit Points Combined with Scalp Acupuncture. Journal of Clinical Acupuncture and Moxibustion. 2020;36(03):24 - 27.

28. Li Si, Yang Yuanqing, Xue Li, Zhou Xingya, Xing Xiaotong. Influence of Nourishing - Blood and Softening - Liver Acupuncture on Plasma 5 - HT of Post - Stroke Depression Patients. Shanghai Journal of Acupuncture and Moxibustion. 2019;38(08):852 - 855.

29. Xie Yanlong, Ji Xuequn, Zhang Ping, Li Si, Wu Hong. Clinical Study on the Treatment of Post - Stroke Depression by Nourishing - Blood and Softening - Liver Acupuncture. Shanghai Journal of Acupuncture and Moxibustion. 2017;36(12):1425 - 1430.

30. Jin Yingai. Clinical Observation on the Treatment of Multiple Cerebral Infarction - Induced Depression by Blood - Letting Puncture Therapy. Chinese Archives of Traditional Chinese Medicine. 2005;(04):752 - 753.

31. Su Zhiwei, Zhang Zhenwei. Treatment of 30 Cases of Post - Stroke Depression by the Method of Pricking Blood to Dredge the Channels. Shaanxi Journal of Traditional Chinese Medicine. 2010;31(12):1651 - 1652.

32. Wang Hongliang, Hu Fangmei, Liu Guohua, Duan Hongbo, Yuan Chunxiao, Cui Youxiang. Influence of Strengthened Yang - Needling at Baihui on the MIP - 1α and NF - κB/iNOS/NO Signaling Pathway in Post - Stroke Depression Patients. Chinese Journal of Integrative Medicine on Cardio - Cerebrovascular Disease. 2021;19(07):1184 - 1188.

33. Wang Hongliang, Cui Youxiang, Hu Fangmei, Liu Guohua, Duan Hongbo, Yuan Chunxiao. Efficacy Observation of Early Adjuvant Treatment of Post - Ischemic Stroke Depression by Strengthened Yang - Needling at Baihui Point. Modern Journal of Integrated Traditional Chinese and Western Medicine. 2020;29(04):363 - 366.

34. Cheng Weiping, Hou Jing. Clinical Experience in the Treatment of Post - Stroke Depression by Strengthened Yang - Needling at Baihui Point. International Journal of Traditional Chinese Medicine. 2012;34(3).

35. Cao Xiaojun, Song Weixi. Clinical Observation on the Treatment of Post - Ischemic Stroke Depression by Acupuncture with the Method of Resolving Phlegm, Relieving Depression and Promoting Blood Circulation to Dredge the Channels. Clinical Journal of Traditional Chinese Medicine. 2014;6(33):7 - 9.

36. Zhang Jinjing, Chen Ruiquan, Xiao Hongbo, et al. Clinical Effect of Huayu Tongluo Moxibustion in the Treatment of Mild - to - Moderate Post - Stroke Depression Patients. China Medical Herald. 2023;20(10):105 - 108.

37. Shen Rong, Yuan Aihong, Tang Youbin, et al. Influence of Huayu Tongluo Moxibustion Combined with Low - Frequency Repetitive Transcranial Magnetic Stimulation on Mild - to - Moderate Post - Stroke Depression Patients. Acta Rehabilitologica Sinica. :1 - 7.

38. Shao Jun, Li Bo, Yuan Aihong, et al. Efficacy Observation of Huayu Tongluo Moxibustion Combined with Press - Needle in the Treatment of Mild - to - Moderate Post - Cerebral Infarction Depression. Chinese Acupuncture & Moxibustion. 2022;42(11):1211 - 1215.

39. Liang Hui, Huang Liwu, Dou Weihua, Wu Peng. Influence of Pricking and Blood - Letting at the Twelve Jing - Well Points on Post - Stroke Depressive State. Guangxi Journal of Traditional Chinese Medicine. 2014;37(01):52 - 54.

40. Jin Lu, Zhang Quan'ai, Di Zhong. Clinical Observation on 50 Cases of Post -

Stroke Depression Treated by the Method of Mai - Li - Jiu Moxibustion at the Twelve Jing - Well Points and Da - Jie - Jing. Zhejiang Journal of Traditional Chinese Medicine. 2021;56(10):745 - 746.

41. Liu Shuying. Efficacy Observation of Awakening the Mind and Opening the Orifices Acupuncture in the Treatment of Post - Stroke Depression under the Stroke Unit Model and Its Influence on Serum Th - Type Cytokines. Shanghai Journal of Acupuncture and Moxibustion. 2022;41(01):35 - 42.

42. Liu Sukun, Zhao Xiumin, Xi Zhimei. Incidence of Post - Stroke Depression and Acupuncture Treatment. Chinese Acupuncture & Moxibustion. 2006;(07):472 - 474.

43. Wang Yanwen, Han Guanxian, Huo Xuewen. Clinical Study on the Treatment of Post - Stroke Depression by the Method of Yuan - Luo Connecting Meridian Moxibustion. Journal of New Chinese Medicine. 2020;52(09):124 - 127.

44. Wang Yang, Wang Yujuan, Wu Jiulong, Xu Tianshu. Influence of Yuan - Luo Mind - Regulating Acupuncture on Serum IGF - 1, BDNF, NGF Levels and Neurological Function in Post - Stroke Depression Patients. Journal of Clinical Acupuncture and Moxibustion. 2020;36(12):17 - 20.

45. Wu Xiaoling. Effect Observation of Oral Administration of Paroxetine Plus Acupuncture at the Eight Confluent Points of the Eight Extraordinary Meridians in the Treatment of Post - Stroke Depression. China Modern Doctor. 2009;47(27):87 - 88.

46. Mi Yiqun, Chen Yi. Comparative Analysis of the Treatment of Post - Stroke Depression with Siguān Points as the Main Therapy. Chinese Journal of Clinical Rehabilitation. 2003;(31):4250.

47. He Dongmei, Tan Lili, Tang Rong, Ma Jing, Huang Qin. Clinical Observation on the Treatment of Mild Post - Stroke Depression (PSD) by Medicated - Separation Moxibustion Based on Timing and Syndrome Differentiation. Journal of Sichuan Traditional Chinese Medicine. 2023;41(01):206 - 210.

48. Wang Dai, He Manman, Li Zhishen, et al. Exploration on the Influence of Jianpi Huatan Decoction Combined with Acupuncture on Serum SP, NPY and Inflammatory Mediators in Post - Stroke Depression Patients Based on the Brain - Gut Axis Theory. Progress in Modern Biomedicine. 2023;23(21):4182 - 4186.

49. Cai Xiaojun, Wang Xuling, Wu Chaoquan, Hu Chunmei, Liu Haifeng. Research on the Immunological Mechanism of the Treatment of Post - Stroke Depression by Multi - Point Injection of Acupuncture and Medicine Combined with Auricular Point Application. Heilongjiang Journal of Traditional Chinese Medicine. 2018;47(02):77 - 79.

50. Cai Xiaojun, Zhang Xiaoyun, Wu Chaoquan, Hu Chunmei, Liu Haifeng, Wang Xuling. Clinical Observation on the Treatment of Post - Stroke Depression by Multi - Point Injection of Acupuncture and Medicine Combined with Auricular Point Application. Heilongjiang Journal of Traditional Chinese Medicine. 2018;47(03):84 - 86.

51. Liu Jiahui, Li Zhaoyuan, Chen Ling, et al. Clinical Efficacy Study on the Treatment of Post - Cerebral Infarction Depression by the Combination of Scalp and Body Acupuncture. Healthy Women. 2023;(3):29 - 30.

52. Bai Qiang. Clinical Observation on the Treatment of Post - Cerebral Infarction Depression by the Combination of Scalp and Body Acupuncture. Chinese Archives of

	Traditional Chinese Medicine. 2011;29(02):409 - 411.<br><br>53. Huang Yong, Chen Jing, Zou Jun. Influence of Scalp Electroacupuncture on Post - Stroke Depressive Disorder. Chinese Journal of Clinical Rehabilitation. 2005;9(40):172 - 173.<br><br>54. Huang Yong, Xia Dongbin, Zou Jun. Clinical Observation on 46 Cases of Post - Stroke Depression Treated by Scalp Electroacupuncture. Chinese Journal of Basic Medicine in Traditional Chinese Medicine. 2003;(02):60 - 62.<br><br>55. Ding Mingqiao, Lei Huimin, He Ling, Cao Shuyi, Tian Huifang. Efficacy Observation of Scalp Electroacupuncture Combined with Kai Si Guan in the Treatment of Post - Stroke Depression. Shanghai Journal of Acupuncture and Moxibustion. 2011;30(10):661 - 662.<br><br>56. Wang Ping, Ji Qingming, Huo Xiaoli. Clinical Observation on the Treatment of Post - Stroke Depression with Scalp Acupuncture as the Main Therapy. Shanghai Journal of Acupuncture and Moxibustion. 2004;(10):15 - 16.<br><br>57. Xie Teng, Yu Zhihong, Wang Yihong. Clinical Efficacy Observation on 30 Cases of Post - Stroke Depression Treated by the Main Method of Withdrawing - Needle Technique of Scalp Acupuncture. Prevention and Treatment of Cardio - Cerebral Vascular Diseases. 2015;15(03):254 - 255 + 257.<br><br>58. Huang Xiaona. Clinical Efficacy Observation on the Treatment of Post - Stroke Depression by the Main Method of Withdrawing - Needle Technique of Scalp Acupuncture. Da Yi Sheng. 2023;8(10):80 - 82.<br><br>59. Song Ying, Liang Haorong. Efficacy Observation of Scalp Acupuncture in the Treatment of Post - Stroke Depression. Shanghai Journal of Acupuncture and Moxibustion. 1999;(01):10 - 11.<br><br>60. Li Xiaojun. Clinical Study on the Treatment of Post - stroke Depression with Head - points as the Main Therapy. Shanghai Journal of Acupuncture and Moxibustion. 2004;(10):13 - 14.<br><br>61. Sang Peng, Wang Huibing. Clinical Observation on the Treatment of Post - stroke Depression by Transcranial Penetrating Acupuncture at Head Acupoints. Chinese Journal of Traditional Chinese Medicine and Pharmacy. 2023;30(05):921 - 924.<br><br>62. Yuan Ping, Zhang Yajuan, Dong Guirong. Clinical Research on the Treatment of Post - stroke Depression by Penetrating Acupuncture at Head Acupoints. Journal of Clinical Acupuncture and Moxibustion. 2006;(11):3 - 4.<br><br>63. Yu Qinming, Wang Dan, Li Qin, Zhang Bo, Chen Hongmei. Clinical Observation on the Treatment of Post - stroke Depression by Acupuncture at Head Acupoints Combined with Yu - Yuan Point Compatibility. Journal of Clinical Acupuncture and Moxibustion. 2019;35(06):27 - 30.<br><br>64. Wang Lihong, Tian Wei, Niu Hua, Mao Dapeng, Yang Tianye, Li Huilan. Clinical Observation on the Effect of "Shen" Acupoints on the Head in the Treatment of Post - stroke Depression. Chinese Medicine Modern Distance Education of China. 2017;15(04):107 - 109.<br><br>65. Sheng Guobin, Gao Chen, Yu Jingkun, Kong Ying, Liu Gang. Clinical Efficacy Observation of Head Electro - acupuncture Therapy in the Treatment of Post - stroke Depression. Information on Traditional Chinese Medicine. 2013;30(03):125 - 126.

66. Wu Yuefeng, Feng Ling, Cai Luojie, Ru Wenya, Xu Xiaofeng, Gong Jianqiu, Ping Renxiang. Intervention of Adjuvant Head Electro - acupuncture Therapy on Post - stroke Depression. Chinese Journal of Physical Medicine and Rehabilitation. 2010;32(4).

67. Dong Jianping, Chen Chen, Li Ying. Influence of Transcranial Penetrating Electro - acupuncture at Head on the Electroencephalogram Changes of Patients with Post - stroke Depression before and after Treatment. Journal of Clinical Acupuncture and Moxibustion. 2013;29(12):17 - 19.

68. Ding Huiqin, Dong Handong, Yang Jingjuan. Clinical Research on the Treatment of Post - stroke Depression by Transcranial Penetrating Electro - acupuncture at Head, Body Acupuncture Combined with Fluoxetine. Journal of New Chinese Medicine. 2020;52(03):143 - 146.

69. Dong Jianping, Xu Yueze, Zhang Yan, Li Ying, Chen Chen. Clinical Research on the Treatment of Post - stroke Depression by Transcranial Penetrating Electro - acupuncture at Head. Chinese Journal of Traditional Chinese Medicine and Pharmacy. 2017;24(04):395 - 398.

70. Dong Jianping, Sun Weiyi, Wang Shun, Wu Zhiquan, Liu Fei. Clinical Observation on the Treatment of Post - stroke Depression by Transcranial Penetrating Electro - acupuncture at Head. Chinese Acupuncture & Moxibustion. 2007;(04):241 - 244.

71. Dong Jianping, Wang Dandan, Tan Jiahui. Clinical Observation on 60 Cases of Post - stroke Depression Treated by Transcranial Penetrating Electro - acupuncture at Head. Heilongjiang Journal of Traditional Chinese Medicine. 2017;46(04):42 - 44.

72. Shen Qin, Qiu Jianzhong, Huang Yong, Guo Jiaquan, Chen Junqi. Clinical Observation on the Improvement of Post - stroke Depressive State by Head Matrix Acupuncture. Liaoning Journal of Traditional Chinese Medicine. 2010;37(04):718 - 720.

73. Dong Yongshu, Liu Xiansong, Xing Shuli. Clinical Efficacy Analysis of Retaining Needles at Head Acupoints in the Treatment of Post - stroke Depression. Medical Information (Medicine and Computer Applications). 2014;0(32).

74. Gong Jin, Cao Zhigang. Treatment of Post - stroke Depression by Transcutaneous Electrical Nerve Stimulation at Head Acupoint Areas. The Journal of Practical Medicine. 2006;(19):2256 - 2257.

75. Liu Xiaohui, Zhong Huiliang. Treatment of 36 Cases of Post - stroke Depression by Combining Head Acupuncture and Body Acupuncture. Lishizhen Medicine and Materia Medica Research. 2006;(07):1276.

76. Fang Ming, Chen Xiao, Sun Lili, et al. Clinical Observation on the Treatment of Post - stroke Depression by Prolonged Retention of Needles in Head Acupuncture. China Practical Medicine. 2023;18(21):127 - 130.

77. Xue Shunhang, Zhu Wenjie, Hou Keqiang, Fang Ming. Effect of Prolonged Retention of Needles in Head Acupuncture Combined with Cerebral Electrical Bionic Stimulation on Patients with Post - stroke Depression. China Contemporary Medicine. 2023;30(13):94 - 97 + 101.

78. Yao Ziqiang. Treatment of 31 Cases of Post - stroke Depression by Head and

Body Acupuncture. Chinese Medicine Modern Distance Education of China. 2013;11(02):61 - 62.

79. Shang Yanjie, Zhou Zhenkun, Xing Yue, Yu Xiaonan, Zhao Fengyun. Clinical Observation on the Treatment of 30 Cases of Post - stroke Depression by Head Acupuncture. Journal of Clinical Acupuncture and Moxibustion. 1999;(12):7 - 8.

80. Liu Jianfeng. Clinical Effect Analysis of Head Acupuncture in the Treatment of Post - stroke Depression. Full - text Edition of Medicine and Health in Chinese Science and Technology Journal Database. 2021;(12).

81. Yang Dingrong. Efficacy Observation on the Treatment of 60 Cases of Post - stroke Depression by Head Acupuncture. Journal of New Chinese Medicine. 2007;(10):29 - 30.

82. Feng Ling, Ru Wenya, Sun Xinfang, Cai Luojie, Du Xiaobei, Wu Yuefeng. Short - term Efficacy Observation of Head Acupuncture in the Prevention and Treatment of Post - stroke Depression. Chinese Journal of Rehabilitation Medicine. 2009;24(05):456 - 457.

83. Shi Caiping. Clinical Efficacy Observation on the Treatment of Post - stroke Depression by Combining Head Acupuncture and Body Acupuncture. Health Preservation. 2016;(4):188.

84. Sun Xianglong. Efficacy Observation on the Treatment of Post - stroke Depression by Combining Head Acupuncture and Electro - acupuncture. Jilin Journal of Traditional Chinese Medicine. 2011;31(11):1092 - 1093.

85. Zhu Yonggang, Zhao Qin, Bai Xinyu, Su Qinglun. Treatment of Post - stroke Depression by Combining Head Acupuncture and Auricular Point Application. Journal of Clinical Acupuncture and Moxibustion. 2012;28(02):17 - 18.

86. Zhu Yonggang, Zhao Qin, Bai Xinyu, Su Qinglun, Xu Yingda, Yang Jin. Clinical Research on the Treatment of Patients with Post - stroke Depression by the Therapy of Combining Head Acupuncture and Auricular Point Application. Journal of Nanjing University of Chinese Medicine. 2014;30(04):323 - 325.

87. Kong Xiangyan. Research on the Influence of Combining Head Acupuncture and Back - shu Points on the Depressive Function of Patients with Post - stroke Depression. Journal of Liaoning University of Traditional Chinese Medicine. 2015;17(07):191 - 193.

88. Liu Xiaoxue, Li Yan, Feng Yao. Clinical Efficacy Observation on the Treatment of Depression after Cerebral Infarction by Combining Head Acupuncture and Body Acupuncture. Chinese Journal of Traditional Chinese Medicine and Pharmacy. 2021;28(05):779 - 780.

89. Yue Lin. Efficacy Observation on the Treatment of Post - stroke Depression by Combining Head Acupuncture and Auricular Point Application. Shanghai Journal of Acupuncture and Moxibustion. 2016;35(07):796 - 798.

90. Yang Shenqiu, Wang Weihua. Efficacy Observation on the Treatment of Post - stroke Depression by Combining Head Acupuncture and Medication. Shanghai Journal of Acupuncture and Moxibustion. 2013;32(01):9 - 11.

91. Liu Fangchao. Efficacy Observation on the Treatment of Post - stroke Depression by Combining Head Acupuncture and Exercise Therapy. Shanghai Journal of

Acupuncture and Moxibustion. 2011;30(11):733 - 734.

92. Sun Yunting, Bao Yehua, Wang Shuling, Chu Jiamei, Li Liping. Efficacy Observation on the Treatment of Post - stroke Depression by Acupuncture at Points Selected According to the Midnight - noon Ebb - flow Theory Combined with Fluoxetine. Chinese Acupuncture & Moxibustion. 2015;35(02):119 - 122.

93. Yu Changbing, Zhang Dan, Luo Qiping, Huang Dan, Ni Xia. Influence of the Treatment of Opening Acupoints with Chu Needle According to the Midnight - noon Ebb - flow Theory on Patients with Post - stroke Depression. Medical Information. 2020;33(4).

94. He Lijuan. Efficacy Observation on the Treatment of Post - stroke Depression by the Midnight - noon Ebb - flow Theory Combined with Flupentixol and Melitracen Tablets. Journal of Clinical Acupuncture and Moxibustion. 2017;33(05):36 - 38.

95. Gu Ting, Wang Ruihui, Ke Zenghui, Wang Dong. Syndrome - differentiated Application of Sun Simiao's Thirteen Ghost Acupoints in the Treatment of Sequelae of Stroke. Journal of Clinical Acupuncture and Moxibustion. 2019;35(09):1 - 4.

96. Wang Yiming, Zhang Xinxin, Yang Xiaoyue. Clinical Research on the Treatment of Post - stroke Depression by Sun's Abdominal Acupuncture. Shandong Journal of Traditional Chinese Medicine. 2020;39(01):26 - 29.

97. Zhang Huili, Wang Wengang, Zhao Xin, et al. Clinical Research on the Treatment of Post - ischemic - stroke Depression by the Acupuncture Method of Calming the Mind and Opening the Orifices. Journal of Hebei University of Chinese Medicine. 2021;36(02):34 - 36.

98. Wang Xiaoyin, Zeng Kexue, Chen Jingjie, Qin Min. Efficacy Observation on the Treatment of 32 Cases of Post - stroke Depression by Scalp Acupuncture for Soothing the Mind and Awakening the Brain. Journal of Yunnan Traditional Chinese Medicine and Materia Medica. 2014;35(05):56 - 57.

99. Li Xiangqiong, Huang Min, Liu Zhaoping, Zhang Jie. Comparison of the Clinical Efficacy of the Liver - soothing and Mind - regulating Acupuncture Method and Pulsed Magnetic Therapy on Patients with Post - stroke Depression. Heilongjiang Medicine. 2023;36(02):265 - 267.

100. Wang Jingxin, Wang Yu, Li Yilan, et al. Clinical Effect of Paroxetine Combined with Traditional Chinese Medicine Acupuncture in the Treatment of Post - stroke Depression. Henan Medical Research. 2019;28(15):2793 - 2795.

101. Mi Xiaojuan. Adjuvant Treatment of 30 Cases of Post - stroke Depression with Paroxetine and Electro - acupuncture. Journal of Practical Traditional Chinese Internal Medicine. 2012;26(12):63 - 64.

102. Sun Yihan, Zhou Jianhua, Zhuang Lixing. Efficacy Observation on the Treatment of Post - stroke Depression by Zhuang's Mind - regulating Acupuncture Method. Journal of Liaoning University of Traditional Chinese Medicine. 2019;21(03):71 - 74.

103. Cheng Yuan, Zhao Jianjun. Randomized Controlled Clinical Study on the Treatment of Post - stroke Depression with Abdominal Acupuncture. Chinese Archives of Traditional Chinese Medicine. 2007;(09):1888 - 1891.

104. Zhou Xiaoyan. Analysis of the Symptom Outcome of Post - stroke Depression

Treated with Acupuncture. China Health Care & Nutrition. 2019;29(33):28.

105. Wu Yuhe. Clinical Research on the Treatment of Depressive Emotions in Stroke Patients with Rehabilitation Acupuncture. China Health Standard Management. 2017;8(04):103 - 104.

106. Feng Hui, Ding Xiaorong. Clinical Research on the Treatment of Post - stroke Depression with the Acupuncture Method of Opening the Orifices, Promoting Qi Circulation and Relieving Depression. Acta Universitatis Traditionis Medicalis Sinensis Pharmacologiaeque Shanghai. 2013;27(03):42 - 44 + 62.

107. Yu Hongwu, Zhu Caifeng, Chen Ying, Hu Jun, Cai Shengchao, Cao Yi. Acupuncture at Siguanshu Points Plus Renzhong for the Treatment of 32 Cases of Post - stroke Depressive State. Journal of Clinical Traditional Chinese Medicine. 2007;(03):245 - 246.

108. Yu Hongwu, Zhu Yan, Pan Yuzhen, et al. Clinical Research on the Prevention of Post - stroke Depression by Acupuncture at Siguanshu Points Plus Renzhong. Journal of Clinical Traditional Chinese Medicine. 2017;29(04):504 - 506.

109. Peng Minhong, Liu Liqun. Efficacy and Prognosis Analysis of the Treatment of Post - stroke Depression by the Method of Opening Siguanshu Points. Heilongjiang Medical Journal. 2020;44(06):748 - 750.

110. Hu Hongping, Lin Jun, Che Dewen. Clinical Observation on the Treatment of 52 Cases of Post - stroke Depression by Opening Siguanshu Points and Awakening the Brain and Opening the Orifices. Psychologies. 2021;16(08):61 - 62 + 64.

111. Ding Da. Efficacy Observation on the Treatment of 25 Cases of Post - stroke Depression by the Acupuncture Method of Opening the Orifices, Relieving Depression and Regulating the Mind. Forum on Traditional Chinese Medicine. 2016;31(05):43 - 44.

112. Ran Jie, Wang Yanjuan, Fan Yonglei. Clinical Efficacy of Abdominal Acupuncture for Guiding Qi to the Origin Combined with Western Medicine in the Treatment of Patients with Post - stroke Depression. Qingdao Medical Journal. 2022;54(1).

113. Li Guozhong. Treatment of Post - stroke Depression by Psychological Rehabilitation Combined with Head and Abdominal Acupuncture. Chinese Journal of Practical Nervous Diseases. 2017;20(13):86 - 88.

114. Wang Wei, Zhang Yawen. Treatment of 70 Cases of Post - stroke Depression by the Method of "Emotional Restraint" Combined with Moxibustion. Abstracts of Medicine and Health in Chinese Science and Technology Journal Database. 2022;(12).

115. Li Yanlan, Zhang Jinhua. Treatment of 60 Cases of Post - stroke Depression by the Method of Regulating Emotions and Desires Combined with Moxibustion. Guangming Journal of Chinese Medicine. 2022;37(06):950 - 952.

116. Liang Hui, Chen Wei, Lin Fei. Clinical Observation on the Treatment of Post - stroke Depressive State Mainly by Blood - letting at the Twelve Jing - points of the Hand. Shanghai Journal of Acupuncture and Moxibustion. 2013;32(06):457 - 458.

117. Tang Jiqin, Sun Yingxia, Dong Bo, Ma Lihong. Influence of Antidepressant Drugs Combined with Liver - soothing and Mind - regulating Acupuncture on the

	Quality of Life of Elderly Patients with Post - stroke Depression. Guiding Journal of Traditional Chinese Medicine and Pharmacy. 2014;20(09):11 - 13.<br><br>118. Liu Lanqing, Han Yuhui, Shi Fangyu. Efficacy Observation on the Treatment of Post - stroke Depression by the Method of Parallel - needling with Multiple Needles Combined with Medication. Shanghai Journal of Acupuncture and Moxibustion. 2022;41(10):970 - 974.<br><br>119. Jin Yu, Wang Fei, Zhang Jinglan, Wang Cui. Clinical Observation on the Treatment of Post - stroke Depression with Press - needles. Journal of Practical Traditional Chinese Medicine. 2023;39(07):1421 - 1423.<br><br>120. Wang Yongliang, Wu Jianli, Bao Rui, et al. Clinical Research on the Treatment of Post - stroke Depression by Press - needles Combined with Yu's Cluster Acupuncture. Journal of Clinical Acupuncture.2021;37(07):15-20.<br>121. Shao Xin, Zhu Chunlin, Yang Wanfang, Tang Xing, Zhang Jicheng. Clinical Study on the Synergistic Effect of Press - Needle at Auricular Points in the Treatment of Post - stroke Depression. Journal of Sichuan of Traditional Chinese Medicine. 2023;41(03):211 - 213.<br><br>122. Huang Bin. Clinical Study on the Treatment of Post - stroke Depression with Press - Needles Combined with Fluoxetine. Journal of New Chinese Medicine. 2021;53(20).<br><br>123. Sheng Yushan. Adjuvant Treatment of 60 Cases of Post - stroke Depression with Venlafaxine Sustained - release Tablets and Electro - acupuncture. Chinese Medicine Modern Distance Education of China. 2013;11(08):37 - 38.<br><br>124. Gao Shan, Li Lei, Yang Faming, Wei Ling. Prevention and Treatment of 40 Cases of Post - stroke Depression by Early Intervention with New Nine - needle Therapy. Shaanxi Journal of Traditional Chinese Medicine. 2013;34(10):1382 - 1383.<br><br>125. Chen Aiwen, Gao Yuan, Wang Guantao, Li Jia, Shen Weidong. Influence of Early Acupuncture Intervention on Post - stroke Depression: A Randomized Controlled Study. Chinese Acupuncture & Moxibustion. 2018;38(11):1141 - 1144.<br><br>126. Tian Xiaowen, Zhang Quanming. Clinical Efficacy Evaluation of Acupuncture with Zhi San Zhen as the Main Method in the Treatment of Post - stroke Depression. Shanghai Journal of Acupuncture and Moxibustion. 2011;30(10):663 - 665.<br><br>127. Yan Xingzhou, Sun Yuming, Xu Lei, Zhang Xueyun, Jiang Yong. Treatment of Post - stroke Depression by Combining Zhi San Zhen with the Method of Nourishing the Heart and Calming the Mind. Journal of Clinical Acupuncture and Moxibustion. 2008;24(12):11 - 12.<br><br>128. Zeng Kexue, Gai Juanjuan, Guo Chunxiu, Wang Dejun. Clinical Observation on 41 Cases of Post - stroke Depression Treated by Zeng's Taiji Acupuncture Method. Hunan Journal of Traditional Chinese Medicine. 2013;29(06):63 - 64.<br><br>129. Huang Jing, Zhang Yining, Pang Xiuyu. Clinical Effect of Bupleurum and Cinnamon Twig Decoction combined with Mind - regulating and Depression - relieving Acupuncture in the Treatment of Post - stroke Depression. China Medical Herald. 2023;20(13):140 - 143 + 165.<br><br>130. Wang Jinhua. Effect of Chaiqin Wendan Decoction Combined with Acupuncture in the Treatment of Post - stroke Depression of Phlegm - heat Disturbing the Interior Type. Inner Mongolia Journal of Traditional Chinese Medicine. 2023;42(06):101 -

102.

131. Wei Ling. Treatment of 60 Cases of Post - stroke Depression with Filiform Needles Combined with Pricking with Plum - blossom Needles. Chinese Journal of Integrative Medicine on Cardio - Cerebrovascular Disease. 2010;8(04):493 - 494.

132. Shi Lihua. Clinical Study on the Treatment of Post - stroke Depression in Young Patients with Fluoxetine Combined with Electro - acupuncture. Youjiang Medical Journal. 2008;(05):538 - 539.

133. Cui Youxiang, Hu Fangmei, Wang Hongliang, Liu Guohua, Duan Hongbo, Yuan Chunxiao. Clinical Efficacy and Safety Analysis of Fluoxetine Combined with Acupuncture Therapy in the Treatment of Post - stroke Depression. China Prescription Drug. 2019;17(06):99 - 100.

134. Zhang Zuopeng, Li Dan. Effect Observation and Safety Analysis of Fluoxetine Combined with Acupuncture in the Treatment of Post - stroke Depression. Journal of North Pharmacy. 2021;18(02):48 - 49.

135. Zhao Hui, Bai Yuyan, Wen Guilian. Efficacy of Citalopram Hydrobromide Tablets Combined with Acupuncture in the Treatment of Post - stroke Depression (PSD) and Its Influence on Neuro - immune Inflammatory Indexes. Chongqing Medicine. 2018;47(14):1952 - 1954 + 1957.

136. Xu Chengyan, Huang Sicong, Huang Shuiming. Clinical Efficacy of Huoxue Shugan Decoction Combined with Auricular Point Pressing Beans in the Treatment of Post - stroke Depression of Liver - depression and Blood - deficiency Type. Journal of Practical Traditional Chinese Internal Medicine. 2023;37(9).

137. Zhang Ji. Analysis of the Clinical Effect of Transcranial Penetrating Electro - acupuncture at the Head in the Treatment of 64 Cases of Post - stroke Depression. Chinese Continuing Medical Education. 2016;8(07):177 - 178.

138. Wang Gaili. Analysis of the Clinical Research on the Treatment of Post - stroke Depression with Acupuncture. Chinese Continuing Medical Education. 2018;10(08):143 - 145.

139. Wu Yin. Discussion on the Clinical Efficacy of Acupuncture in the Treatment of Post - stroke Depression. Contemporary Medicine Forum. 2014;12(20):11 - 12.

140. Shuang Yuan. Brief Discussion on the Clinical Efficacy and Safety of Acupuncture in the Treatment of Post - stroke Depression. Full - text Database of Medicine and Health in Chinese Science and Technology Journal Database. 2022;(4).

141. Huang Yan, Yang Bin. Efficacy Observation on the Treatment of 112 Cases of Post - stroke Depression of Heart - spleen Deficiency Type with Superficial Acupuncture. Strait Pharmaceutical Journal. 2016;28(12):220 - 221.

142. Deng Yanli, Yang Degong. Clinical Observation on 30 Cases of Post - stroke Depression of Qi - stagnation and Phlegm - depression Syndrome Treated with Mild Moxibustion. Jiangsu Journal of Traditional Chinese Medicine. 2013;45(5).

143. Deng Yanli, Tu Chengyun, Liu Qiao, Yang Degong. Clinical Observation on 40 Cases of Post - stroke Depression of Liver - qi Stagnation Syndrome Treated with Mild Moxibustion. Inner Mongolia Journal of Traditional Chinese Medicine. 2014;33(35):94 - 95.

144. Zhao Xiufen, Huang Bixia, Yao Rujie. Influence of Mild Moxibustion Combined with Emotional Nursing on Patients with Post - stroke Depression. Journal of Snake. 2019;31(03):391 - 392 + 416.

145. Chen Jun. Efficacy Observation on the Treatment of Post - stroke Depression of Liver - depression, Spleen - deficiency and Phlegm - dampness Endogenous Type with Wendan Jieyu Decoction Combined with Mind - regulating and Qi - regulating Acupuncture. Chinese Manipulation & Rehabilitation Medicine. 2018;9(12):48 - 50.

146. Pan Qiulan, Wang Haiyan, Zhang Limei. Clinical Observation on the Treatment of Post - stroke Depression with Warm - needling Moxibustion. Journal of Emergency in Traditional Chinese Medicine. 2008;(06):748 - 749.

147. Yang Xiaorui, Lian Jianping. Clinical Observation on the Treatment of Post - stroke Depression with Fire - needle Combined with Mind - regulating Acupuncture. World Latest Medicine Information (Continuous Electronic Journal). 2020;20(28):157, 159.

148. Guo Ruiyou, Su Li, Liu Lian, Wang Caixia. Influence of the Linggui Bafa Method on the Efficacy and Quality of Life of Post - stroke Depression Patients. Chinese Acupuncture & Moxibustion. 2009;29(10):785 - 790.

149. Wang Lihong, Tian Wei, Niu Hua, Mao Dapeng. Clinical Observation on the Treatment of Post - stroke Depression with the Linggui Bafa Method Combined with Cognitive Behavioral Therapy. Chinese Medicine Modern Distance Education of China. 2018;16(22):92 - 95.

150. Wan Guoqiang, Ma Hongmei, Zhou Guoxiang. Clinical Research on the Treatment of Post - stroke Depression with Heat - sensitive Moxibustion. Journal of Clinical Acupuncture and Moxibustion. 2011;27(07):9 - 11.

151. Wang Zhaoling, Wang Liling, Jiang Yongping, Li Yewu, Hu Jianping. Clinical Efficacy Observation on the Treatment of Post - stroke Depression with Heat - sensitive Moxibustion Combined with Acupuncture. Practical Clinical Journal of Integrated Traditional Chinese and Western Medicine. 2014;14(10):72 - 73.

152. Gao Fan, Deng Kesui, Liu Jiao, Huang Jianhua, Zhang Qunfang, Rao Ting, Song Huimin, Li Xinyuan. Clinical Observation on the Treatment of Post - stroke Depression with Heat - sensitive Moxibustion Combined with Modified Morita Therapy. Jiangxi Journal of Traditional Chinese Medicine. 2019;50(12).

153. Sun Jianhong, Huang Chunmin, Mao Yadi. Research on the Synergistic Effect of Bio - wave Acupoint Stimulation on Post - stroke Depression Patients. Zhejiang Journal of Traumatic Surgery. 2022;27(5).

154. Bao Wenyang. Analysis of the Effect of Head - acupuncture Therapy in the Treatment of Post - stroke Depression. Contemporary Medicine Forum. 2016;14(09):29 - 30.

155. Huang Shile, Wei Ying, Zhang Ziru. Clinical Randomized Controlled Study on the Treatment of Post - stroke Depression with Electro - acupuncture at "Shenwu Xing Group Acupoints" Combined with Western Medicine. Shanghai Journal of Traditional Chinese Medicine. 2014;48(07):33 - 36.

156. Wu Xiaoyun, Huo Xiaoning. Controlled Study on the Treatment of Post - stroke Depression with Electro - acupuncture at Sanyinjiao and Yintang. Gansu Journal of Traditional Chinese Medicine. 2007;(06):53 - 54.

157. Jiao Haifeng, Liu Wanming, Hao Xizhi, Zhang Liang. Clinical Observation on 33 Cases of Post - stroke Depression Treated with Electro - acupuncture and Paroxetine. Medical Recapitulate. 2012;18(06):950 - 951.

158. You Xueru. Systematic Review on the Comparison between Electro - acupuncture and Antidepressant Drugs in the Treatment of Post - stroke Depression. Healthy Women. 2021;(41):146.

159. Chen Xinzhi, Wang Jian, Wang Jinghui, Zang Lixin. Clinical Research on the Treatment of Post - stroke Depression with the Electro - acupuncture Method of Nourishing the Brain and Calming the Mind. Liaoning Journal of Traditional Chinese Medicine. 2005;(05):464 - 465.

160. Jiang Hong, Wen Xinyi, Shi Tinghui, Zhang Suming. Efficacy Observation on the Treatment of Depressive State after Lacunar Cerebral Infarction with Electro - acupuncture Stimulation. Chinese Journal of Physical Medicine and Rehabilitation. 2006;(12):807 - 810.

161. Jiang Hong, Zhang Suming, Shi Tinghui, Wen Xinyi. Application of Electro - acupuncture in the Treatment of Post - stroke Depression in the Elderly. Chinese Journal of Gerontology. 2007;(11):1101 - 1103.

162. Li Xiaoyan, Shi Guochen, Dong Qinjian. Treatment of 70 Cases of Post - stroke Depression Patients with Electro - acupuncture at Tou Sanshen and Si Guan Points. Heilongjiang Journal of Traditional Chinese Medicine. 2013;42(01):31 - 32.

163. Tan Feng, Meng Di, Liang Yangui, Tan Jiuqing. Research on the Influence of Electro - acupuncture on the Neurological Function of Patients with Acute Post - stroke Depression. Journal of Emergency in Traditional Chinese Medicine. 2017;26(02):198 - 200.

164. Peng Huiyuan, He Xijun, Zhao Minghua. Influence of Electro - acupuncture on the Activities of Daily Living and Quality of Life of Post - stroke Depression Patients. Shanghai Journal of Acupuncture and Moxibustion. 2010;29(07):425 - 427.

165. Li Juebao, Ye Xiangming, Cheng Ruidong, Zhu Genying, Yang Ting. Influence of Electro - acupuncture on the Cerebral Blood Flow of Post - stroke Depression Patients. Chinese Journal of Rehabilitation Theory and Practice. 2015;21(02):192 - 195.

166. Zhu Genying, Ye Xiangming, Li Juebao, Wen Wanshun, Tian Liang, Yang Ting. Clinical Controlled Study on the Treatment of Post - stroke Depression with Electro - acupuncture at "Si Guan" and Fluoxetine Hydrochloride. Zhejiang Journal of Integrated Traditional Chinese and Western Medicine. 2012;22(11):865 - 867.

167. Zuo Yaochen, Lou Bidan, Liu Kong, Gao Yuyan. Efficacy of Electro - acupuncture at Pericardium Meridian Acupoints in the Treatment of Post - stroke Depression and Its Influence on Serum ACTH and Cort in Patients. Journal of Clinical Acupuncture and Moxibustion. 2023;39(04):53 - 57.

168. Zhuang Ziq, Wang Dunjian. Efficacy Evaluation of Electro - acupuncture at Zhi San Zhen in the Treatment of Post - stroke Depression and Its Influence on the Quality of Life. Liaoning Journal of Traditional Chinese Medicine. 2009;36(08):1400 - 1402.

169. Cui Hai. Clinical Observation on the Treatment of 30 Cases of Post - stroke

Depression with Electro - acupuncture. Journal of Zhejiang College of Traditional Chinese Medicine. 2005;(02):65 - 66.

170. Zhou Zhiming. Controlled Study on Electro - acupuncture Treatment and Drug Treatment for Post - stroke Depression. China Medical Herald. 2007;(16):23 + 128.

171. Zhou Yuan, Jin Jianhong, Zhou Guoying. Treatment of 145 Cases of Post - stroke Depression with Electro - acupuncture. Journal of Shaanxi University of Traditional Chinese Medicine. 2010;33(04):78 - 80.

172. Nie Bin, Nie Tao. Clinical Research on the Treatment of Post - stroke Depression with Electro - acupuncture. Shanghai Journal of Acupuncture and Moxibustion. 2006;(09):6 - 8.

173. Dai Wei. Efficacy Observation on the Treatment of Post - stroke Depression with Electro - acupuncture. Hubei Journal of Traditional Chinese Medicine. 2009;31(09):22 - 23.

174. Wang Zhonghua. Efficacy Observation on the Treatment of Post - stroke Depression with Electro - acupuncture. Chinese Journal of Rehabilitation Medicine. 2008;(11):1032 - 1034.

175. Tang Jixiang, Guan Nianhong, Li Lin, Liu Jufen. Efficacy of Electro - acupuncture in the Treatment of Post - stroke Depression and Its Influence on the Quality of Life of Patients. Shanghai Journal of Acupuncture and Moxibustion. 2003;(03):12 - 14.

176. Ji Shufeng. Treatment of 30 Cases of Post - stroke Depression with Electro - acupuncture. Chinese Journal of Integrative Medicine on Cardio - Cerebrovascular Disease. 2008;(07):868.

177. Zhu Jie. Influence of Electro - acupuncture Treatment on the Symptoms and Cognitive Function of Post - stroke Depression. Chinese Journal of Clinical Rehabilitation. 2005;(32):32 - 34.

178. Xu Guoqing, Miao Guangyu. Efficacy Observation on the Treatment of Post - stroke Depression in the Elderly with Electro - acupuncture. Chinese Journal of Convalescent Medicine. 2015;24(04):384 - 386.

179. Xian Yimin, Liang Yan'an, Zhu Lihua, Huang Kangbai. Efficacy Observation on the Treatment of Post - ischemic - stroke Depression of Liver - qi Stagnation and Qi - deficiency and Blood - stasis Type with Electro - acupuncture. Journal of New Chinese Medicine. 2018;50(09):185 - 187.

180. Rong Qi, Jin Ze, Jiang Xicheng, Sun Yuanzheng. Efficacy Observation on the Treatment of 60 Cases of Post - stroke Depression with Electro - acupuncture. Journal of Clinical Acupuncture and Moxibustion. 2005;(03):36 - 37.

182. Huang Liang, Sun Zhongren. Clinical Observation on the Treatment of Post - stroke Depression with Electro - acupuncture. Journal of Clinical Acupuncture and Moxibustion. 2005;(05):48 - 49.

183. Long Haowen, Tan Peizhen, Feng Jianhong, Li Meizhi. Clinical Observation on the Treatment of Post - stroke Depression with Electro - acupuncture. Journal of Clinical Psychiatry. 2004;(03):173 - 174.

184. Tang Rong'e. Analysis of the Efficacy of Electro - acupuncture in the Treatment of Post - stroke Depression and Its Influence on the Neurological Function of Patients. Chinese Community Doctors. 2021;37(20):81 - 82.

185. Remile·Rezak. Analysis of the Efficacy of Electro - acupuncture in the Treatment of Post - stroke Depression and Its Influence on the Neurological Function of Patients. World Latest Medicine Information Digest. 2022;22(3):57 - 59.

186. Kang Weige, Yang Baogang. Clinical Efficacy Observation of Electro - acupuncture Therapy on Post - stroke Depression. Journal of Clinical Acupuncture and Moxibustion. 2014;30(11):35 - 37.

187. Hou Dongfen, Luo Hechun. Clinical Efficacy Observation on the Treatment of 30 Post - stroke Depression Patients with Electro - acupuncture at Baihui and Yintang. Chinese Acupuncture & Moxibustion. 1996;(08):23 - 24.

188. Hou Dongfen, Luo Hechun. Clinical Efficacy Observation on the Treatment of 30 Post - stroke Depression Patients with Electro - acupuncture at Baihui and Yintang Acupoints. Acta Chinese Medicine and Pharmacology. 1996;(02):34.

189. Jia Junli. Clinical Efficacy Observation on the Treatment of Post - stroke Depression with Electro - acupuncture at Baihui and Renzhong. Medical Journal of Chinese People's Health. 2009;21(12).

190. Gao Min, Wang Xiaoyi. Efficacy Observation on the Treatment of Post - stroke Depression with Electro - acupuncture at Governor Vessel Acupoints Combined with Venlafaxine Sustained - Release Capsules. Massage & Rehabilitation Medicine. 2013;4(8).

191. Chen Dong, Sun Yuanzheng. Clinical Observation on the Treatment of Post - stroke Depression with Electro - acupuncture Combined with Auricular Point Application. Clinical Journal of Traditional Chinese Medicine. 2016;8(02):35 - 36.

192. Sun Haidong, Wang Fei. Treatment of 22 Cases of Post - stroke Depression with Electro - acupuncture at Auricular Points. Journal of External Therapy of Traditional Chinese Medicine. 2015;24(01):38 - 39.

193. Zhang Jinglan, Wang Fei. Treatment of 34 Cases of Post - stroke Depression with Electro - acupuncture at Auricular Points. Journal of External Therapy of Traditional Chinese Medicine. 2013;22(01):46 - 47.

194. Guo Aisong, Li Aihong, Chen Xin, Cai Junyan, Wu Qinfeng. Influence of Electro - acupuncture Combined with Fluoxetine on the Depressive State and Neurological Function of Post - stroke Depression Patients. Shandong Medical Journal. 2011;51(25):9 - 11.

195. Dong Zhiheng, Liu Yongli, Zhang Yintao, Bai Yunfei. Influence of Electro - acupuncture Combined with Escitalopram on the Depressive State and Neurological Function of Post - stroke Depression Patients. China Health Care & Nutrition. 2016;26(12):92.

196. Zheng Hua, Sun Baomin, Lü Yanhua, Yuan Xiaojing. Efficacy Observation on the Treatment of Post - stroke Depression with Electro - acupuncture Combined with Citalopram. Chinese Journal of Physical Medicine and Rehabilitation. 2010;32(7).

197. Liu Yuhong. Treatment of 43 Cases of Post - stroke Depression with Electro - acupuncture Combined with Deanxit. Chinese Medicine Modern Distance Education

of China. 2016;14(04):107 - 108.

198. You Yang. Efficacy Observation on the Treatment of Post - stroke Depression with Electro - acupuncture at Back - shu Points Combined with a Low Dose of Seroxat. Chinese Journal of Convalescent Medicine. 2013;22(11):974 - 976.

199. Zhang Rui, Yan Ping. Efficacy Observation on the Treatment of 70 Cases of Post - stroke Depression with Electro - acupuncture at the "Jieyu Prescription" Acupoints. Journal of Shanxi University of Chinese Medicine. 2017;18(05):39 - 41.

200. Li Li, Jiang Jiali. Clinical Observation on the Treatment of Post - stroke Depression with Electro - acupuncture Combined with Paroxetine. Journal of Gansu College of Traditional Chinese Medicine. 2011;28(01):53 - 54.

201. Dong Fachang. Effect Observation on the Treatment of Post - stroke Depression with Electro - acupuncture Combined with Fluoxetine. China Medical Herald. 2007;(06):31 + 112.

202. Gu Ruohuai. Effect Observation on the Treatment of Post - stroke Depression with Electro - acupuncture Combined with Fluoxetine. Psychologist (Second Half Month Edition). 2012;(3):155 - 155.

203. Peng Huiyuan, Tan Jilin, Wang Benguo, Zhao Minghua. Clinical Research on the Treatment of Post - stroke Depression with Electro - acupuncture mainly at the Temporal Three - needle. Journal of New Chinese Medicine. 2009;41(10):89 - 90 + 8.

204. Bai Jing, Li Baodong. Influence of Early Intervention with Electro - nape - acupuncture on the Occurrence of Depressive Events in Acute Cerebral Infarction Patients. Chinese Journal of Rehabilitation Theory and Practice. 2007;13(11).

205. Yang Yingguo, Li Baodong, Bai Jing, Wang Zhiyong, Zhao Rongzhong, Wang Lichun. Efficacy Observation on the Treatment of Depression after Acute Cerebral Infarction with Electro - nape - acupuncture. Chinese Journal of Physical Medicine and Rehabilitation. 2012;34(2).

206. Wang Mingli, Wang Xiaoxia, Zhao Yingdan. Clinical Efficacy and Partial Mechanism of Electro - nape - acupuncture Combined with Xiaoyao San in the Treatment of Post - stroke Depression. Journal of Clinical and Experimental Medicine. 2018;17(2).

207. Ding Hao. Efficacy and Safety Analysis of Shugan Jieyu Decoction Combined with Acupuncture in the Treatment of Depression after Cerebral Infarction. Clinical Journal of Traditional Chinese Medicine. 2020;12(34):34 - 37.

208. Chen Xuanya, Wang Xiaoqiao, Zhao Can. Clinical Effect of Shugan Tiaoshen Acupuncture Method Combined with Progressive Muscle Relaxation Training in the Treatment of Depression after Cerebral Infarction. China Medicine. 2023;18(09):1391 - 1395.

209. Wu Jinlong, Yang Kun, Li Youfeng, You Yuanyuan, Ye Bihong. Clinical Efficacy Observation of Acupuncture with the Method of Soothing the Liver and Dredging the Collaterals Combined with Paroxetine in the Treatment of Post - stroke Depression and Its Influence on the Expression Levels of Neurotransmitters and Cytokines. Chinese Archives of Traditional Chinese Medicine. :1 - 10.

210. Zhang Fangxin, Li Wan, Wu Zhiming, Wang Jie. Clinical Research on the Prolonged Retention of Needles at Baihui Point in the Treatment of Post - stroke

Depression. Journal of External Therapy of Traditional Chinese Medicine. 2023;32(02):1 - 3.

211. Cui Youxiang, Wang Hongliang, Liu Guohua, Duan Hongbo, Yuan Chunxiao, Hu Fangmei. Influence of Strengthened Yang - Pricking at Baihui Point on Antioxidant Enzymes and the Nrf2/HO - 1 Pathway in Post - stroke Depression Patients. Acta Chinese Medicine and Pharmacology. 2021;49(2).

212. Wang Chengyin, Huang Dehong, Huang Jianhong, et al. Efficacy Observation on the Treatment of Depression after Cerebral Infarction with Acupuncture at Baihui Point plus Point Injection of Erigeron Breviscapus. Journal of Zhejiang Chinese Medical University. 2010;34(03):400 - 401 + 404.

213. Huang Dehong, Wang Chengyin, Huang Jianhong, Ye Yu, Chen Xiuhui. Treatment of Depression after Cerebral Infarction with Acupuncture at Baihui Point plus Point Injection of Scutellaria Baicalensis Injection. Chinese Journal of Clinical Rehabilitation. 2004;(28):6132 - 6133.

214. Fan Yuying. Clinical Observation on the Treatment of Depression after Cerebral Infarction with Acupuncture at Baihui Point plus Point Injection. Journal of Practical Traditional Chinese Internal Medicine. 2019;33(10):66 - 68.

215. Chen Xiuhui, Huang Dehong, Liu Yanrong. Influence of Acupuncture at Baihui Point plus Point Injection in the Treatment of Depression after Cerebral Infarction on Neurotransmitters. China Journal of Traditional Chinese Medicine and Pharmacy. 2010;25(07):1150 - 1152.

216. Wang Yali, Yao Li. Efficacy Observation on the Treatment of Post - stroke Depression with Baixiao Moxibustion. Shanghai Journal of Acupuncture and Moxibustion. 2015;34(06):595 - 596.

217. Liu Tao, Chen Xiaoou, Han Jingxian. Treatment of 30 Cases of Post - stroke Depression with the Acupuncture Method of Supplementing Qi, Regulating Blood and Strengthening the Root and Cultivating the Yuan. Shaanxi Journal of Traditional Chinese Medicine. 2013;34(01):68 - 70.

218. Ma Qiuping, Pang Yong, Ji Si. Efficacy Evaluation of the Kidney - tonifying and Governor - vessel - regulating Acupuncture Method on Post - stroke Depression Patients and Its Influence on the Quality of Life. Liaoning Journal of Traditional Chinese Medicine. 2015;42(06):1319 - 1322.

219. Miao Fugui. Clinical Observation on the Treatment of 32 Cases of Depression after Cerebral Infarction with Fluoxetine Hydrochloride Combined with Moxibustion at Acupoints. Psychologist. 2018;24(15):101.

220. Chen Juan, Dai Wenxin, Lin Zhou, Yan Fen. Clinical Observation on the Treatment of 126 Cases of Severe Depression after Cerebral Infarction with Fluoxetine Hydrochloride Combined with Acupuncture. Modern Preventive Medicine. 2008;(19):3837 - 3838 + 3840.

221. Sang Peng, Qu Yushan. Clinical Observation on the Treatment of Depressive State after Cerebral Infarction with Electro - acupuncture in the Eye Area. Chinese Journal of Traditional Chinese Medicine and Pharmacy. 2018;25(04):574 - 575.

222. Huang Chunyuan. Clinical Analysis of the Treatment of Depressive State after Cerebral Infarction with Eye - acupuncture. Journal of Liaoning University of Traditional Chinese Medicine. 2013;15(8).

223. Zhou Hongfei, Xu Jinping, Wang Jian. Efficacy Observation on the Treatment of Post - stroke Depression with Eye - acupuncture Therapy plus Fluoxetine. Liaoning Journal of Traditional Chinese Medicine. 2008;35(1).

224. Wang Pengqin, Wang Xiaoxi. Clinical Observation on the Treatment of Post - stroke Depression with Eye - acupuncture Combined with Jieyu Pills. Shanxi Journal of Traditional Chinese Medicine. 2021;37(8).

225. Jiang Meirong. Efficacy Observation on the Treatment of Post - stroke Depression of Liver - qi Stagnation Type with Eye - acupuncture Combined with Heat - sensitive Moxibustion. Henan Journal of Traditional Chinese Medicine. 2022;42(09):1406 - 1409.

226. Wang Pengqin, Wu Bin. Clinical Observation on the Treatment of Post - stroke Depression with Eye - acupuncture Combined with Xiaoyao San. Journal of Practical Traditional Chinese Medicine. 2021;37(2).

227. Zhao Xuan, Li Xiaopeng, Li Jian, Tian Bin, Wang Guangjun. Efficacy of Magnetic Bead Auricular Point Application Combined with Repetitive Transcranial Magnetic Stimulation on Post - stroke Depression. Acta Academiae Medicinae Shandong University. 2022;60(01):65 - 70.

228. Yan Yingxia, Shen Huiping. Influence of Community Psychological Intervention Combined with Acupoint Massage on First - onset Post - stroke Depression. Hebei Journal of Traditional Chinese Medicine. 2015;37(08):1244 - 1246.

229. Chen Yatian, Lin Xidan, Li Qiaozhen. Clinical Observation on the Effect of Point Embedding at "Five - Emotion Points" on Post - stroke Depression. Guide of China Medicine. 2023;21(21).

230. Zhang Xidong, Tan Jianxin. Treatment of Post - stroke Depression by Point Injection with Classification. Guangming Journal of Chinese Medicine. 2009;24(07):1335 - 1336.

231. Wang Wei, Zhang Zixuan, Li Kaige. Efficacy Observation on the Treatment of Post - stroke Depression of Liver - depression and Spleen - deficiency Type with Point Moxibustion Combined with Fluoxetine. Chinese Journal of Modern Drug Application. 2024;18(5).

232. Chang Feng. Efficacy Observation on the Treatment of Post - stroke Depression of Liver - depression and Spleen - deficiency Type with Point Moxibustion Combined with Fluoxetine. Journal of Practical Traditional Chinese Medicine. 2022;38(07):1140 - 1142.

233. Chen Jinyong, He Yuqin, Lin Xiaofeng, et al. Research on the Effect of Point Application Combined with Auricular Point Pressing in Post - stroke Depression Patients and Its Influence on Insomnia. Oriental Medicated Diet. 2020;(6):18.

234. Liu Jing. Clinical Effect of Point Application Combined with Auricular Point Pressing in the Treatment of Post - stroke Depression Patients and Its Influence on Insomnia. Reflexology and Rehabilitation Medicine. 2022;3(5).

235. Li Wenjing. Clinical Efficacy Observation on the Treatment of Post - stroke Depression with Point Injection of Medicine and Acupuncture Combined with Flupentixol and Melitracen Tablets. Chinese Journal of Integrative Medicine on Cardio - Cerebrovascular Disease. 2020;18(16).

236. Liao Xiaoying, Su Guang, Zhou Peng, Zhong Liling, Song Lan. Clinical Efficacy Observation of Fine Moxibustion Therapy in Improving Post - stroke Depression. Journal of Hubei University of Chinese Medicine. 2020;22(4).

237. Li Xiangqiong, Chen Rui. Clinical Observation on the Adjuvant Treatment of Post - stroke Depression with Fine Moxibustion Therapy. Chinese Folk Remedies. 2023;31(23):32 - 35.

238. Wang Yongjun, Chen Sulian, Wang Chunfang, et al. Observation on the Event - related Potentials of Post - stroke Depression with Transcutaneous Low - frequency Point Electrical Stimulation. Journal of Clinical Acupuncture and Moxibustion. 2015;31(09):43 - 45.

239. Wang Yongjun, He Jia, Sun Changcheng, Zhang Yimin, Du Jingang, Zhao Jianguo. Clinical Research on Transcutaneous Point Electrical Stimulation and Acupuncture in the Treatment of Post - stroke Depression. Chinese Journal of Rehabilitation Medicine. 2014;29(08):751 - 753.

240. Zhang Tian. Clinical Analysis of the Treatment of Depressive State after Ischemic Stroke with the Combination of Acupuncture and Medicine. Medical Aesthetics and Cosmetology. 2020;29(13):107.

241. Huang Qiong. Clinical Observation on the Treatment of Post - stroke Depression with Luo's Nourishing - Spirit and Regulating - Mind Acupuncture Method. Chinese Journal of Traditional Chinese Medicine and Pharmacy. 2019;26(02):314 + 317.

242. Jin Qi, Fang Yi, Li Jin, Tao Wenjing, Wu Xiaoliang. Influence of Auricular - point Pressure Intervention on the Clinical Efficacy of Comprehensive Treatment for Post - stroke Depression:附30 Clinical Cases. Jiangsu Journal of Traditional Chinese Medicine. 2018;50(3).

243. Chen Lingyan, Lin Peizheng, Lin Zhuoyin. Treatment of 31 Cases of Post - stroke Depression with Auricular - point Pressure Combined with Electro - acupuncture. Chinese Medicine Modern Distance Education of China. 2015;13(06):71 - 73.

244. Zhang Miao. Clinical Observation on the Treatment of Post - stroke Depression of Liver - kidney Yin - deficiency Type with Auricular - point Pressure Combined with Acupuncture. Shanghai Journal of Acupuncture and Moxibustion. 2011;30(02):90 - 92.

245. Tian Meixin, Yang Shuping, Yang Qing. Research on the Influence of Auricular - point Bean - pressing Method on the Depressive State of Stroke Patients with Zhong - jingluo Syndrome. Chinese Journal of Nursing Education. 2014;11(06):409 - 411.

246. Deng Liangbin, Shi Yijie. Clinical Observation on the Treatment of Post - stroke Depression with Auricular - point Bean - pressing Combined with Fluoxetine Hydrochloride. Chinese Folk Remedies. 2022;30(14).

247. Zheng Lihong. Influence of Auricular - point Bean - embedding on the Depressive State of First - onset Stroke Patients. Chinese General Practice Nursing. 2013;11(25).

248. Zhou Juan. Observation on the Effect of Nursing Intervention of Auricular - point Bean - embedding on the Depressive State of First - onset Stroke Patients. Inner Mongolia Journal of Traditional Chinese Medicine. 2017;36(17).

249. Li Leilei, Liu Zhixiu, Liu Jing, et al. Influence of Auricular - point Bean - embedding Therapy on Insomnia in Post - stroke Depression Patients. Journal of Clinical Traditional Chinese Medicine. 2013;(11).

250. Wang Fei, Sun Haidong. Treatment of 60 Cases of Post - stroke Depression with Auricular - point Therapy. Practical Clinical Journal of Integrated Traditional Chinese and Western Medicine. 2016;16(02):42 - 43.

251. Chen Xiyuan, Li Xiuyun, Hu Yufang. Research on the Treatment of Depression in Stroke Patients with Auricular - point Therapy. Henan Journal of Traditional Chinese Medicine. 2014;34(B06).

252. Jiao Yonggang, Xing Yilan. Research on the Improvement Effect of Auricular - point Electro - acupuncture on Post - stroke Depression Symptoms in Type 2 Diabetic Patients. Journal of New Medicine. 2018;49(12):889 - 893.

253. Sun Yongsheng, Qi Hui, Wang Fei. Clinical Observation on the Adjuvant Treatment of Post - stroke Depression with Auricular - point Electro - acupuncture Combined with Body Acupuncture. Shanxi Journal of Traditional Chinese Medicine. 2020;36(11):32 - 33.

254. Zhao Wenjing, Zhang Jinglan. Clinical Observation on the Treatment of Post - stroke Depression with Auricular - point Electro - acupuncture Combined with Acupuncture. Journal of External Therapy of Traditional Chinese Medicine. 2023;32(04):88 - 90.

255. Huang Hongmin, Zhao Guangfeng, Wang Jiayan, et al. Randomized Controlled Study on the Treatment of Post - stroke Depression (Convalescent Stage of Stroke) with Auricular - point Application. Hainan Medical Journal. 2012;23(24):29 - 30.

256. Huang Hongmin, Zhao Guangfeng, Chen Qi, et al. Randomized Controlled Study on the Treatment of Post - stroke Depression with Auricular - point Application. Journal of Liaoning University of Traditional Chinese Medicine. 2011;13(09):11 - 13.

257. Jin Chongmin, Zhu Xianfen, Wu Zhaoyang. Effect Observation of Auricular - point Application Combined with Acupuncture in the Treatment of Post - stroke Depression. Chinese Journal of Rural Medicine and Pharmacy. 2020;27(19):11 - 12.

258. Yang Qing, Li Xiaoqing, Yang Shuping, Tian Meixin. Application of Auricular - acupuncture in the Prevention of Depression in Post - stroke Patients. Chinese Nursing Research. 2016;30(05):620 - 621.

259. Liu Jinlan, Zhao Jingdong, Qiu Limin. Efficacy Observation on the Treatment of 36 Cases of Post - stroke Depression with Auricular - acupuncture. Journal of Practical Traditional Chinese Internal Medicine. 2006;(05):555.

260. Yang Qing, Yang Shuping, Li Xiaoqing. Influence of Auricular - acupuncture Method on Mildly Depressed Patients after Stroke. Guangming Journal of Chinese Medicine. 2015;30(03):550 - 552.

261. Pang Qingmin, Zhao Yuxiao, Shao Suju, Li Hongzhang, Wang Chenghui. Efficacy Observation on the Treatment of Post - stroke Depression with埋线 at Back - shu Points Combined with Shugan Jieyu Capsules. Shanghai Journal of Acupuncture and Moxibustion. 2022;41(2):128 - 133.

262. Liu Zhiliang, Lao Jinxiong, Pan Qingjie. Clinical Observation on the Intervention of Warm - needling Moxibustion at Back - shu Points in Post - stroke Depression. Shanghai Journal of Acupuncture and Moxibustion. 2013;32(04):255 - 257.

263. Wang Dandan. Clinical Research on the Treatment of Post - stroke Depression with Warm - needling Moxibustion at Back - shu Points Combined with Acanthopanax Senticosus Injection. Chinese Journal of Integrative Medicine on Cardio - Cerebrovascular Disease. 2023;21(21):4011 - 4015.

264. Huang Weiling, Chen Wei, Wu Xiaoyuan, Peng Xiaowen. Efficacy Observation on the Treatment of Post - stroke Depression with Acupuncture by the Method of Coordinating Five - Tones according to the Zang - Organ Time. World Journal of Integrated Traditional and Western Medicine. 2021;16(12):2334 - 2337.

265. Zhang Shuhong, Zhang Weiwei. Research on the Effectiveness of Acupuncture in the Treatment of Post - stroke Depression Patients. Health Must - Read. 2022;(9):145 - 146.

266. Bi Chongxia, Lin Shaohua, Jiang Ling. Controlled Study on the Effects of Different Therapies for Post - stroke Depressive State. China Practical Medicine. 2010;5(02):71 - 73.

267. Zhang Xuesong, Xu Erhe. Efficacy Observation on 94 Cases of Post - stroke Depression. Chinese Journal of Gerontology. 2007;27(24):2431 - 2432.

268. Liao Baidan. Efficacy Observation on the Treatment of Post - stroke Depression with Acupuncture. Chinese Journal of Modern Drug Application. 2017;11(10):122 - 124.

269. Kong Fanwu. Clinical Research on the Acupuncture Therapy for Post - stroke Depression. China Foreign Medical Treatment. 2009;28(26):95.

270. Li Meirong. Analysis of the Acupuncture Treatment Effect for Post - stroke Depression. Electronic Journal of Clinical Medical Literature. 2020;7(28):28 + 37.

271. Zhao Junhong. Acupuncture Treatment for Post - stroke Depression. Chinese Journal of Rehabilitation. 2002;(01):31.

272. Zhang Shumei, Chang Li, Qu Wen, Yuan Fang. Effect Observation of Emotional Nursing and Auricular - point Seed - embedding for Stroke Patients with Comorbid Depression in the Acute Stage. Chinese Nursing Research. 2012;26(16):1503 - 1504.

273. Gan Xuansheng. Clinical Effect Analysis of the Combined Treatment of Moxibustion at Baihui Point and Self - made Yujin Prescription for Patients with Depression after Cerebral Infarction. Clinical Journal of Traditional Chinese Medicine. 2021;13(08):74 - 76.

274. Jiang Yafen, Pan Jinhua, Wu Jiangxia, Wu Lixiao, Zhang Lianxin. Effect Analysis of Acupuncture with Points Selected Based on the Brain - gut Axis Theory in the Treatment of Post - stroke Depression. The World of Health. 2023;(20):67 - 68.

275. Gan Jian, Hu Yaping, Peng Mei, Li Ruizheng, Li Yuanshi. Clinical Research on the Treatment of Post - stroke Depression with the "Abdomen - back Bagua" Acupuncture - moxibustion Therapy. Journal of Sichuan of Traditional Chinese Medicine. 2010;28(04):118 - 120.

	276. Jia Yibo, Feng Xianxia, Luo Kai, Zhou Youlong. Treatment of 72 Cases of Post - stroke Depression of Liver - depression Transforming into Fire Type with Yin - yang Point Compatibility of the Abdomen and Back. Traditional Chinese Medicine Research. 2016;29(11):54 - 56.<br><br>277. Zuo Yun. Clinical Observation on the Treatment of Post - stroke Depression of Liver - depression Transforming into Fire Type with Yin - yang Point Compatibility of the Abdomen and Back. Diet and Health. 2018;5(25):98 - 99.<br><br>278. Chi Lei, Li Xiaoning, Wu Lei. Efficacy Observation on the Treatment of Post - stroke Depression with the Group of "Yinqi Guiyuan" Abdominal - acupuncture Points Combined with Medication. Journal of Clinical Acupuncture and Moxibustion. 2016;32(3).<br><br>279. Cheng Yuan, Tang Qisheng. Randomized Controlled Observation on the Treatment of Post - stroke Depression of Liver - depression and Spleen - deficiency Syndrome in the Elderly with Abdominal - acupuncture. Chinese Journal of Tissue Engineering Research and Clinical Rehabilitation. 2007;(39):7791 - 7794.<br><br>280. Liang Hongqun, Bi Xueqi, Xiong Qin, Wei Min. Application and Effect Analysis of Abdominal - acupuncture Therapy in the Treatment of Post - stroke Depression. Chinese Journal of Geriatric Care. 2017;15(5).<br><br>281. Zhang Ekeng, Wang Jing, Zhang Jin. Clinical Research on the Treatment of Post - stroke Depression with Abdominal - acupuncture Therapy. Acta Chinese Medicine. 2016;31(10):1623 - 1625.<br><br>282. Qin Yuanwen. Efficacy Observation and Mechanism Discussion of Self - made Yujin Prescription Combined with Moxibustion at Baihui Point in the Treatment of Depression after Cerebral Infarction. Journal of Sichuan of Traditional Chinese Medicine. 2016;34(07):97 - 99.<br><br>283. Liu Fang, Luo Yaohui, Luo Junyu. Clinical Observation on the Treatment of Post - stroke Depression with Tongue - acupuncture. China Health Care & Nutrition. 2017;27(3):308 - 309.<br><br>284. Luo Tong, Wang Wusheng. Clinical Research on the Treatment of Post - stroke Depression with Moxibustion. Chinese Medicine Modern Distance Education of China. 2011;9(08):140 - 141.<br><br>285. Ou Fengjuan. Clinical Observation on the Treatment of Post - stroke Depression with Moxibustion. Smart Healthcare. 2022;8(31):72 - 75.<br><br>286. Liu Songlin, Yao Jiangxia. Influence of Moxibustion Therapy on the Autonomic Nervous Function of Post - stroke Depression Patients. Clinical Research and Practice. 2019;4(05):131 - 133.<br><br>287. Sun Guochao, Chang Junhua. Clinical Observation on the Treatment of 60 Cases of Depression after Cerebral Infarction with Moxibustion at Baihui Point. Clinical Journal of Traditional Chinese Medicine. 2015;7(05):99.<br><br>288. Yang Huinan, Huang Yan, Chen Jialiang. Influence of Moxibustion Combined with Auricular - point Bean - embedding on the Emotions and Neurological Function of Post - stroke Depression Patients. Family Medicine. Medical Selection. 2020;(3):96.<br><br>289. Lü Xuexia, Zhang Ting, Wang Famning, Ding Xiaomei, Zheng Shuangshuang.

	Clinical Observation on the Treatment of Post - stroke Depression with Moxibustion Combined with Fluoxetine. Zhejiang Clinical Medical Journal. 2016;18(11).<br><br>290. Xing Kun. Efficacy Observation on the Treatment of 45 Cases of Post - stroke Depression with Moxibustion at Back - shu Points. Hebei Journal of Traditional Chinese Medicine. 2010;32(05):728 - 729.<br><br>291. Niu Hongyue, Li Zhen. Treatment of 36 Cases of Post - stroke Depression with the Method of Regulating Sanjiao by Long - needle. Inner Mongolia Journal of Traditional Chinese Medicine. 2018;37(3).<br><br>292. Chen Xingsheng, Zhou Ting, Zhang Wendong, et al. Efficacy Observation on the Treatment of Post - stroke Depression with Penetrating Long - needle. Journal of Clinical Traditional Chinese Medicine. 2010;22(11):996 - 997.<br><br>293. Yi Fei, Ma Yonghua. Efficacy Observation on the Treatment of Depressive State in the Convalescent Stage of Cerebral Infarction with Escitalopram Oxalate Combined with Acupuncture. Chinese Science and Technology Journal Database (Abstract Edition) of Medicine and Health. 2023;(2).<br><br>294. Du Guoying. Efficacy Observation on the Treatment of Depressive State in the Convalescent Stage of Cerebral Infarction with Escitalopram Oxalate Combined with Acupuncture. Inner Mongolia Journal of Traditional Chinese Medicine. 2020;39(04):136 - 137.<br><br>295. Qian Xiaolu, You Yanli, Shu Shi, Zhou Xuan, Zhou Shuang, Zhu Qin. Health Economics Evaluation of the Rongnao Xingshen Acupuncture Method in the Treatment of Post - stroke Depression. Journal of Zhejiang Chinese Medical University. 2015;39(09):700 - 703.<br><br>296. Huangfu Li, Yang Quyun, Liu Fang, Liu Tong. Efficacy Observation on the Effect of Dong's "Strange Three - needle" Acupoints on Post - stroke Depression. Chinese Archives of Traditional Chinese Medicine. 2018;36(10):2325 - 2328.<br><br>297. Feng Yong, Xiao Huiling, Lin Renyong, Huang Yong. Clinical Efficacy Observation on the Treatment of Post - stroke Depression with Bo's Abdominal - acupuncture. Journal of Clinical Acupuncture and Moxibustion. 2011;27(10):33 - 35.<br><br>298. Yuan Chen, Liu Na, Liu Shuqing. Clinical Effect of Acupuncture for Promoting Qi Circulation, Dredging the Governor Vessel and Regulating the Mind in the Treatment of Post - stroke Depression Patients and Its Influence on Neurological Function Analysis. Health Must - Read. 2022;(14):117 - 119.<br><br>299. Ma Yuanxin, Xu Weiguang, Wei Yanan. Efficacy Observation on the Treatment of Post - stroke Depression with Western Medicine Combined with Acupuncture Method of Awakening the Brain, Soothing the Mind and Relieving Depression. Journal of Guangzhou University of Chinese Medicine. 2022;39(01):93 - 98.<br><br>300. Feng Guifang, Ye Fuxian, Yi Zhaohao. Application of Western Medicine Combined with Acupuncture at Head Acupoints and Back - shu Points of the Five Zang - organs in the Treatment of Post - stroke Depression Patients. Journal of External Therapy of Traditional Chinese Medicine. 2020;29(03):18 - 19.<br><br>301. Zhang Shengli, Lou Yuanmin, Huang Yonghong. Observation on the Effect of Citalopram Combined with Electro - acupuncture in the Treatment of Post - stroke Depression. Medical Equipment. 2016;29(14):77 - 78.

302. Li Hongmei. Observation on the Clinical Efficacy of Acupuncture in the Treatment of Post - stroke Depression. Diet and Health. 2019;6(29):7.

303. Niu Huiyan, Zhang Zhanqiang, Kong Li. Treatment of Post - stroke Depression with the "Depression - Relieving and Mind - Soothing" Acupuncture Method Combined with Early Psychological Rehabilitation Therapy. Liaoning Journal of Traditional Chinese Medicine. 2022;49(11):179 - 182.

304. Li Dandan, Sun Zhao, Wu Zhu, Shen Wei. Clinical Efficacy Observation on the Treatment of Post - stroke Depression of Liver - qi Stagnation Type with the Depression - Relieving and Mind - Soothing Acupuncture Method Combined with Mirtazapine. China Journal of Traditional Chinese Medicine and Pharmacy. 2022;37(03):1826 - 1829.

305. Lin Yu, Wang Yanxu, Li Shiju, Ruan Su, Liang Hui. Treatment of 30 Cases of Post - stroke Depression with the "Depression - Relieving and Mind - Regulating" Acupuncture Method. Fujian Journal of Traditional Chinese Medicine. 2023;54(07):14 - 15 + 39.

306. Jiao Shouchun, Wang Zhengyu. Analysis of the Effect of Acupuncture on the Levels of Serum 5 - HT, NE and BDNF in Patients with Post - stroke Depression. World Latest Medicine Information. 2018;18(19):149.

307. Gou Yanhua, Pi Min, Kang Ping, Yang Zhuoxin. Influence of the Governor - Vessel - Regulating and Du - Vessel - Dredging Acupuncture Method on Event - related Potential P3 in Post - stroke Depression Patients. China Foreign Medical Treatment. 2014;33(07):18 - 19 + 21.

308. Liu Qun, Chen Jie, Wu Ting, Tian Qiang, Wang Ying. Clinical Research on the Treatment of Post - stroke Depression with the Heart - Regulating and Mind - Soothing Acupuncture Method. Journal of Clinical Acupuncture and Moxibustion. 2021;37(07):24 - 28.

309. Lin Yu, Wang Yanxu, Li Shiju, Liang Hui, Xu Lei. Treatment of 30 Cases of Post - stroke Depression with the Acupuncture Method of "Regulating the Marrow - Sea and Relieving Depression and Soothing the Mind". Fujian Journal of Traditional Chinese Medicine. 2017;48(03):14 - 15.

310. Chen Lizao, Zhang Hong. Efficacy Observation on the Treatment of Post - stroke Depression with the Acupuncture Method of "Regulating the Marrow - Sea" Combined with Auricular - point Application. Shanghai Journal of Acupuncture and Moxibustion. 2011;30(08):525 - 526.

311. Liu Zhishun, Liu Jun, Huang Man, et al. Clinical Observation on the Treatment of 30 Cases of Post - stroke Depression with the Method of Regulating the Marrow - Sea. Chinese Acupuncture & Moxibustion. 1997;(09):543 - 544.

312. Jiang Liyan, Kang Jian. Clinical Observation on the Treatment of Post - stroke Depression with the Acupuncture Method of Regulating the Marrow - Sea, Relieving Depression and Soothing the Mind. Liaoning Journal of Traditional Chinese Medicine. 2007;(03):346 - 347.

313. Yu Xueping, Sun Guiping. Efficacy Observation on the Treatment of Post - stroke Depression with the Governor - Vessel - Regulating and Conception - Vessel - Dredging Acupuncture Method. Shanghai Journal of Acupuncture and Moxibustion. 2016;35(03):273 - 275.

314. Wang Yanjun, Han Yixu, Hu Yutong, Zhang Lihua. Efficacy Evaluation of the Governor - Vessel - Regulating and Brain - Dredging Acupuncture Method in the Treatment of Post - stroke Depression Patients with Comorbid Anxiety (English). World Journal of Acupuncture - Moxibustion (English Edition). 2018;(1):4 - 9, 73 - 74.

315. Zhang Lihua, Wang Yanjun, Xue Weihua. Influence of the Governor - Vessel - Regulating and Brain - Dredging Acupuncture Method on the Anxiety State of Post - stroke Depression Patients. Journal of Traditional Chinese Medicine. 2016;57(18):1574 - 1578.

316. Wang Yanjun, Li Gefei, Wang Yebo, et al. Efficacy Observation on the Treatment of Post - stroke Depression with the Governor - Vessel - Regulating and Brain - Dredging Acupuncture Method. Hebei Journal of Traditional Chinese Medicine. 2019;41(06):927 - 930.

317. Guo Xiaofang, Lian Lingxia, Wang Yanjun. Clinical Efficacy of the Governor - Vessel - Regulating and Brain - Dredging Acupuncture Method in the Treatment of Depression after Cerebral Infarction. World Chinese Medicine. 2018;13(03):687 - 690.

318. Zhang Peng, Zou Wei. Efficacy Observation on the Treatment of Post - stroke Depression with the Governor - Vessel - Regulating Acupuncture Method. Heilongjiang Science and Technology Information. 2016;(29):161 - 162.

319. Gao Yan. Clinical Observation on the "Mind - Regulating" Acupuncture Method in the Treatment of Post - stroke Depression. World Latest Medicine Information (Continuous Electronic Journal). 2021;21(9):197 - 198.

320. Chen Shunxi, Liu Feifei. Efficacy and Scale Score Correlation Analysis of the "Mind - Regulating and Depression - Relieving Acupuncture Method" Combined with Psychological Intervention in the Treatment of Mild Post - stroke Depression. Acupuncture Research. 2018;43(01):38 - 42.

321. Li Qijiao, Sa Ren, Lin Zhipeng, Lin Yi. Efficacy of the Mind - Regulating and Depression - Relieving Acupuncture Method Combined with Thunder - Fire Moxibustion in the Treatment of Post - stroke Depression and Its Influence on the Levels of Serum IL - 2, IL - 6 and TNF - α. Journal of Clinical Acupuncture and Moxibustion. 2022;38(08):14 - 18.

322. Chen Shunxi, Liu Feifei. Clinical Observation on the Treatment of Mild Post - stroke Depression with the Mind - Regulating and Depression - Relieving Acupuncture Method Combined with Psychological Intervention. Shanghai Journal of Acupuncture and Moxibustion. 2017;36(08):914 - 917.

323. Li Yan, Liu Rui, Wang Ying, Xu Wenyuan. Treatment of 36 Cases of Post - stroke Depression with the Mind - Regulating and Qi - Regulating Acupuncture Method. Journal of Clinical Acupuncture and Moxibustion. 2009;25(01):22 - 23.

324. Ye Qingjing. Clinical Efficacy Analysis of the Mind - Regulating and Liver - Soothing Acupuncture Method Combined with Fluoxetine in the Treatment of 43 Cases of Post - stroke Depression. Chinese Journal of Ethnomedicine and Ethnopharmacy. 2013;22(23):62.

325. Tong Xin, Liu Dandan, Wei Yan, Kou Jiyou, Yang Tiansong, Qiao Lida. Intervention Effect of the Mind - Regulating and Liver - Soothing Acupuncture Method on Post - stroke Depression. China Medicine and Pharmacy. 2012;2(03):127 -

128.

326. Lin Qin, Jiang Jingjing, Huang Guirong, Su Yingying, Zou Yulong. Clinical Effect of the Mind - Regulating and Liver - Soothing Acupuncture Method in the Treatment of Post - stroke Depression. China Contemporary Medicine. 2022;29(12):125 - 128.

327. Wei Xifang, Zhang Jingruo, Hu Chen, Cai Wa, Shen Weidong. Clinical Research on the Treatment of Post - stroke Depression with the Mind - Regulating and Liver - Soothing Acupuncture Method. Chinese Journal of Information on Traditional Chinese Medicine. 2023;30(07):145 - 150.

328. Cai Wa, Wei Xifang, Zhang Jingruo, Zhang Kun, Liu Ran, Shen Weidong. Clinical Research on the Improvement of Depressive Symptoms and Neurological Function in Post - stroke Depression Patients with the Mind - Regulating and Liver - Soothing Acupuncture Method Combined with Fluoxetine. Journal of Clinical Acupuncture and Moxibustion. 2022;38(07):15 - 19.

329. Ni Siming, Jiang Xingzhuo, Peng Yongjun. Acupuncture with the Mind - Regulating and Depression - Relieving Method Combined with Sertraline Hydrochloride Tablets in the Treatment of Post - stroke Depression: A Randomized Controlled Trial. Chinese Acupuncture & Moxibustion. 2023;43(01):19 - 22 + 66.

330. Zhang Yincui, Wang Yujun, Lian Jianlun, et al. Analysis of the Clinical Value of the Mind - Regulating and Depression - Relieving Acupuncture Method in the Treatment of Depression after Cerebral Infarction of Liver - qi Stagnation Type. Healthy Women. 2021;(23):118.

331. Duan Xidong, Li Shouyan, Wan Haopeng. Clinical Observation on the Treatment of Depression after Cerebral Infarction of Liver - qi Stagnation Type with the Mind - Regulating and Depression - Relieving Acupuncture Method. Journal of Shandong University of Traditional Chinese Medicine. 2019;43(03):284 - 287.

332. Sun Yan, Gao Yang, Wang Runze, Sun Yingzhe. Influence of the Mind - Regulating and Pivot - Moving Method in the Treatment of Post - stroke Depression on Intestinal Flora, Their Metabolites and Related Neurotransmitters: A Randomized Controlled Trial. Journal of Clinical Acupuncture and Moxibustion. 2024;40(01):33 - 38.

333. Wu Juan, Zhou Zhiliang, Xu Jiaying, Pang De, Luo Yangfei. Treatment of 30 Cases of Post - stroke Depression with the Mind - Regulating and Collateral - Dredging Acupuncture Method. Henan Journal of Traditional Chinese Medicine. 2014;34(03):415 - 416.

334. Duan Dapeng. Efficacy Observation on the Treatment of Post - stroke Depression with the Mind - Regulating and Collateral - Dredging Acupuncture Method Combined with the Traditional Chinese Medicine Huoxue Tiaoshen Jieyu Decoction. Chinese Community Doctors. 2015;31(13):81 + 83.

335. Huang Jie, Li Baodong, Zhang Shiliang, et al. Efficacy Observation on the Treatment of Post - stroke Depression with the Mind - Regulating Acupuncture Method Combined with Deanxit. World Chinese Medicine. 2015;10(08):1238 - 1241.

336. Li Xia, Zhou Danfeng, Xu Lei, Zhong Binmo. Clinical Observation on the Treatment of Post - stroke Depression with the Mind - Regulating Acupuncture Combined with Neck Three - needle. Massage & Rehabilitation Medicine. 2021;12(4).

337. Zhang Ji, Wang Wei, Wu Huahui, Liu Sijia, Wang Yanming. Clinical Observation on the Treatment of 60 Cases of Post - stroke Depression with the Jieyu Tongluo Decoction Combined with the Electro - acupuncture Penetrating - point Therapy. World Latest Medicine Information. 2015;15(94):52 - 53 + 59.

338. Wu Fuzeng, Pang Shaojun. Clinical Research on the Treatment of Post - stroke Depression with Xiaoyao Pill Combined with Acupuncture. Guiding Journal of Traditional Chinese Medicine and Pharmacy. 2014;20(14):92 - 94.

339. Yao Zhenyu, Yang Jiruo, Yan Chunlu. Clinical Efficacy Observation on the Treatment of Post - stroke Depression with Xiaoyao San Combined with Acupuncture. Gansu Medical Journal. 2018;37(01):67 - 68.

340. Wu Yun, Sun Xinghua, Xing Yue. Intervention Effect of Penetrating Acupuncture on Post - stroke Depression and Its Influence on Plasma Cortisol Level. Shanghai Journal of Acupuncture and Moxibustion. 2011;30(03):153 - 154.

341. Ouyang Basi, Wu Siqi. Clinical Observation on the Treatment of Post - stroke Depression of Liver - kidney Deficiency Type with Penetrating Acupuncture. Shanxi Journal of Traditional Chinese Medicine. 2020;36(5).

342. Li Shuzhi, Song Manping, Hu Bingcheng. Efficacy Observation on the Treatment of Depression after Stroke with Penetrating - point Acupuncture. China Science and Technology Information. 2005;(09):136.

343. Pan Bo. Efficacy Study on the Treatment of Post - stroke Depression with Penetrating - point Acupuncture Combined with Minor Bupleurum Decoction. China Practical Medicine. 2018;13(06):102 - 103.

344. Wan Haopeng, Jin Ye, Yu Zhenyu. Efficacy Observation on the Treatment of Depression after Cerebral Infarction with the Zhuyu Xiaoyao Decoction plus Acupuncture at Baihui Point. The Frontier of Medicine. 2013;(35):119 - 120, 121.

345. Chen Yanfang, Liu Yan. Clinical Efficacy of the Zhuyu Xiaoyao Decoction Combined with Acupuncture at Siguanshu Points in the Treatment of Post - stroke Depression and Its Influence on Neurological Function. Hebei Journal of Traditional Chinese Medicine. 2022;44(12):2005 - 2007 + 2011.

346. Li Wenying, Zeng Xiudi. Application Effect of Tongyuan Acupuncture Method in Post - stroke Depression Patients. Reflexology and Rehabilitation Medicine. 2022;3(15):10 - 13.

347. Wu Jinjin, Cui Shaoyang, Wang Shuhui, Lai Xinsheng. Clinical Efficacy Observation on the Treatment of Post - stroke Depression of Liver - qi Stagnation Type with Tongyuan Acupuncture Method. Journal of Guangzhou University of Chinese Medicine. 2022;39(10):2303 - 2308.

348. Xiao Wei, Wang Zhen, Kong Hongbing, et al. Clinical Research on the Treatment of Post - stroke Depression with the "Governor - Vessel - Dredging and Depression - Treating" Acupuncture Method. Journal of Gansu College of Traditional Chinese Medicine. 2015;32(04):55 - 60.

349. Wang Baoguo, Xiao Wei, Wang Zhen, et al. Influence of the "Governor - Vessel - Dredging and Depression - Treating" Acupuncture Method in the Treatment of Post - stroke Depression on Clinical Symptoms and Serum 5 - HT, NE, DA. Chinese Archives of Traditional Chinese Medicine. 2023;42(03):190 - 193.

350. Wang Guanchao, Xiao Wei, Zhang Xianbao, et al. Research on the Influence of the Governor - Vessel - Dredging and Depression - Treating Acupuncture Method on the Levels of Serum 5 - HT, NE and BDNF in Post - stroke Depression Patients. Journal of Gansu College of Traditional Chinese Medicine. 2015;32(02):58 - 62.

351. Wang Zhen, Zheng Zhijun, Liang Fajun, Zhang Xianbao, Xiao Wei. Clinical Research on the Treatment of Post - stroke Depression with the Governor - Vessel - Dredging and Depression - Treating Acupuncture Method. Journal of New Chinese Medicine. 2019;51(04):227 - 230.

352. Wang Xian, Yuan Hongli, Zeng Jie, Xu Jingyang, Ma Junhu. Influence of the Governor - Vessel - Dredging and Depression - Treating Acupuncture Method in the Treatment of Post - stroke Depression on the Levels of Neurotransmitters. Journal of Clinical Acupuncture and Moxibustion. 2018;34(12):17 - 20.

353. Zheng Quancheng, Zhang Yu, Zeng Linghui, Huang Xinyi, Fu Yongjun. Efficacy of the Governor - Vessel - Dredging and Depression - Treating Acupuncture Method Combined with Head - acupuncture Therapy in the Treatment of Post - stroke Depression and Its Influence on the Levels of Serum 5 - HT and BDNF. Journal of Clinical Acupuncture and Moxibustion. 2021;37(09):25 - 28.

354. Qiao Li. Clinical Observation on the Treatment of Post - stroke Depression with the Governor - Vessel - Dredging and Depression - Relieving Acupuncture Method. Chinese Medicine Modern Distance Education of China. 2023;21(17):128 - 130.

355. Sun Peiyang, Chu Haoran, Li Peifang, et al. Influence of the Governor - Vessel - Dredging and Mind - Regulating Acupuncture Intervention on the Drug Treatment of Post - stroke Depression. Chinese Acupuncture & Moxibustion. 2015;35(08):753 - 757.

356. Wang Yougang, Gao Dahong, Mu Daozhou, et al. Efficacy Observation on the Treatment of Post - stroke Depression with the Governor - Vessel - Dredging and Mind - Regulating Acupuncture Method Combined with Auricular - point Pill - Pressing. Journal of Anhui University of Chinese Medicine. 2018;37(04):44 - 48.

357. Yan Xiaofei, Zhang Yonggen, Zhang Wendong. Influence of the Governor - Vessel - Dredging and Mind - Regulating Acupuncture Method on COR in PSD Patients and Clinical Research. Medical Information. 2018;31(15):137 - 139 + 142.

358. Chen Jiajia. Analysis of the Effect of the Governor - Vessel - Dredging and Mind - Regulating Acupuncture Method on Effectively Improving Depressive Symptoms, Neurological Deficit Symptoms and Quality of Life of Patients. Healthful Friend. 2021;(5):88.

359. Chang Xueli, Zhang Zhifang, Yu Dongge, Zhang Hongli. Influence of the Governor - Vessel - Dredging and Mind - Regulating Acupuncture Method on the Neurological Function and Serum Cor, IL - 2, IL - 6 Levels in Post - stroke Depression Patients. Information on Traditional Chinese Medicine. 2021;38(01):59 - 62.

360. Wang Lihong. Treatment of Post - ischemic - stroke Depression with the Governor - Vessel - Dredging and Mind - Regulating Acupuncture Method. Acta Chinese Medicine. 2019;34(07):1555 - 1558.

361. Sun Peiyang, Chu Haoran, Li Peifang, et al. Treatment of Post - stroke Depression with Governor - Vessel - Dredging and Mind - Regulating Acupuncture

Method: A Randomized Controlled Study. Chinese Acupuncture & Moxibustion. 2013;33(01):3 - 7.

362. Sun Peiyang, Wang Ying, Li Peifang. Treatment of 62 Cases of Post - stroke Depression with Governor - Vessel - Dredging and Mind - Regulating Acupuncture Method. Psychologist (Second Half Month Edition). 2012;(2):138.

363. Huang Wenxiong, Yu Xiumei, Zhi Qiming. Efficacy Observation on the Treatment of Post - stroke Depression with Governor - Vessel - Dredging and Mind - Regulating Acupuncture Method. Journal of Clinical Acupuncture and Moxibustion. 2017;33(04):13 - 16.

364. Liang Ziwei, Su Jiabao, He Yankun, Lan Junchao, Zhang Guangzheng. Influence of Governor - Vessel - Dredging and Mind - Regulating Acupuncture Method Combined with Danshen Chuanxiongqin Injection on Serum Neurotrophic Indexes and Monoamine Neurotransmitters in Post - stroke Depression Patients. Progress in Modern Biomedicine. 2022;22(18):3480 - 3484.

365. Ji Jie, Wang Hairong, Zhao Hong. Efficacy of Governor - Vessel - Dredging and Mind - Regulating Acupuncture Method Combined with Danshen Chuanxiongqin Injection in the Adjuvant Treatment of Post - stroke Depression and Its Influence on Neuroimmune Inflammatory Indexes. Liaoning Journal of Traditional Chinese Medicine. 2019;46(05):1055 - 1058.

366. He Xijun, Lai Xinsheng, Tan Jilin, Wang Benguo. Efficacy Observation on the Treatment of 118 Cases of Post - stroke Depression with Governor - Vessel - Dredging and Brain - Awakening Acupuncture Method. Journal of New Chinese Medicine. 2006;(08):60 - 61.

367. Qin Feng. Efficacy Observation on the Treatment of Post - stroke Depression with Brain - Dredging and Collateral - Activating Acupuncture Therapy. Electronic Journal of Clinical Medical Literature. 2016;3(45):8896 + 8898.

368. Zheng Meifeng, Zhang Yongshu, Ruan Chuanliang, Wang Xiangbin, He Furong. Influence of the Governor - Vessel and Conception - Vessel - Regulating Acupuncture Method on Initial - onset Post - stroke Depression. Journal of Fujian College of Traditional Chinese Medicine. 2010;20(01):16 - 18.

369. Wang Zhengyang, He Yaqi, Li Dandan, et al. Clinical Efficacy of the Acupuncture Method of Awakening the Mind and Opening the Orifices on Post - stroke Depression Patients and Its Influence on Serum C - reactive Protein and Interleukin 10. World Journal of Integrated Traditional and Western Medicine. 2020;15(01):165 - 169.

370. Wang Bo, Wang Tianlei, Tan Chunfeng, Huang Jianfu, Xu Qiong. Efficacy of the Acupuncture Method of Awakening the Mind and Opening the Orifices on Post - stroke Depression and Its Influence on 5 - HT, NGF and IL - 23. Chinese Archives of Traditional Chinese Medicine. 2020;38(12):84 - 86.

371. Luo Wenjun, He Yaqi, Li Dandan, Zhou Jianyu, Wang Zhengyang, Li Mengyao. Influence of the Acupuncture Method of Awakening the Mind and Opening the Orifices on Serum Tumor Necrosis Factor and Interleukin 6 in Post - stroke Depression. Journal of Clinical Traditional Chinese Medicine. 2020;32(02):295 - 299.

372. Chen Lujie, Sun Fangyuan, He Yaqi, Li Dandan. Efficacy of the Acupuncture Method of Awakening the Mind and Opening the Orifices in the Treatment of Post - stroke Depression and Its Influence on the Serum 5 - HT Content. Guiding Journal of

Traditional Chinese Medicine and Pharmacy. 2018;24(14):38 - 40 + 43.

373. Tian Ming, Zheng Dan. Research on the Influence of the Acupuncture Method of Awakening the Mind and Relieving Depression on the Clinical Efficacy, SAS and HAMD Scale Scores, and 5 - HT Level in Post - stroke Depression Patients. Journal of Apoplexy and Nervous Diseases. 2021;38(06):518 - 521.

374. Hong Zhenmei, Wang Zhanglian, Zhang Shuqing, Ma Ruijie. Efficacy Observation on the Treatment of Post - stroke Depression with the Acupuncture Method of Awakening the Mind and Relieving Depression. Journal of Zhejiang Chinese Medical University. 2015;39(08):621 - 624.

375. Li Yanan. Treatment of 240 Cases of Post - stroke Depression with the Acupuncture Method of Awakening the Mind and Relieving Depression. Chinese Journal of Gerontology. 2014;34(21):6177 - 6178.

376. Wang Yongtian, Song Shuchang, Wang Yueying, et al. Observation on the Timeliness of the "Brain - Awakening, Mind - Soothing, and Liver - Soothing and Depression - Relieving" Acupuncture Method in the Treatment of Post - stroke Depression. Electronic Journal of Integrative Medicine on Cardio - Cerebrovascular Disease. 2017;5(09):87 - 88.

377. Ma Yuanxin. Research on the Influence of the Brain - Awakening, Mind - Soothing, and Liver - Soothing and Depression - Relieving Acupuncture Method on the Neurological Function Rehabilitation of Post - stroke Depression Patients. Modern Journal of Integrated Traditional Chinese and Western Medicine. 2021;30(02):150 - 154.

378. Wang Hui. Efficacy Observation on the Treatment of Post - stroke Depression with the Brain - Awakening, Mind - Soothing, and Depression - Relieving Acupuncture Method. Shanghai Journal of Acupuncture and Moxibustion. 2016;35(04):416 - 418.

379. Ge Jieying, Song Shuchang, Lu Zhi, et al. Efficacy Observation on the Neurological Function Rehabilitation of Post - stroke Depression Patients with the Brain - Awakening, Mind - Soothing, and Collateral - Dredging Acupuncture Method. Journal of Emergency in Traditional Chinese Medicine. 2014;23(04):627 - 628.

380. Wang Qinghai, Song Shuchang, Feng Nana, Cui Jingjun. Efficacy Observation on the Treatment of 60 Cases of Post - stroke Depression with the Brain - Awakening, Mind - Soothing, and Collateral - Dredging Acupuncture Method. Chinese Journal of Traditional Chinese Medicine and Pharmacy. 2015;22(01):73 - 74.

381. Zhang Yiwei, Liang Hui, Ke Jinshi. Treatment of Post - stroke Depression with the "Brain - Awakening and Orifice - Opening, Depression - Relieving and Mind - Soothing" Acupuncture Method. China Modern Doctor. 2018;56(36):117 - 120.

382. Li Chunmei, Li Meng. Clinical Observation on 80 Cases of Post - stroke Depression Treated with the "Brain - Awakening and Orifice - Opening" Acupuncture Method. Chinese Journal of Integrative Medicine on Cardio - Cerebrovascular Disease. 2003;(09):549 - 550.

383. Yang Fan, Zhan Daowei, Jin Wenjie, Hu Wei, Xu Yingmei, Qian Lifeng. Effect of Early Intervention with the "Brain - Awakening and Orifice - Opening" Acupuncture Method on the Prevention of Post - stroke Depression and the Recovery of Neurological Function. Zhejiang Clinical Medical Journal. 2022;24(07):992 - 993 + 996.

	384. Zhang Xiaotong. Treatment of 60 Cases of Post - stroke Depression with the Brain - Awakening and Orifice - Opening Acupuncture Method. Shaanxi Journal of Traditional Chinese Medicine. 2008;(06):710 - 711.<br><br>385. Gu Chengguang, Chen Liping. Influence of the Brain - Awakening and Orifice - Opening Acupuncture Method on the Incidence of Post - stroke Depression. Journal of Practical Medicine. 2006;(03):315.<br><br>386. Zhang Caizhen. Efficacy Observation on the Treatment of 45 Cases of Post - stroke Depression with the Brain - Awakening and Orifice - Opening Acupuncture Method. Journal of New Chinese Medicine. 2004;(08):50 - 51.<br><br>387. Li Chunmei, Li Meng. Treatment of 80 Cases of Post - stroke Depression with the Brain - Awakening and Orifice - Opening Acupuncture Method. Shanghai Journal of Acupuncture and Moxibustion. 2005;(02):23.<br><br>388. He Xijun, Tan Jilin, Wang Benguo, Lai Yingying. Efficacy Observation on the Treatment of 86 Cases of Post - stroke Depression with the Brain - Awakening and Orifice - Opening Acupuncture Method. Chinese Journal of Rehabilitation Theory and Practice. 2005;(06):467 - 468.<br><br>389. Zhao Hong. Stratified Randomized Controlled Study on the Treatment of 100 Cases of Post - stroke Depression with the Brain - Awakening and Orifice - Opening Acupuncture Method. Chinese Journal of Clinical Rehabilitation. 2003;(31):4286.<br><br>390. Chen Wanmin, Feng Wanxia, Pan Hongshan. Treatment of 29 Cases of Post - stroke Depression with the Brain - Awakening and Orifice - Opening Acupuncture Method Combined with Sertraline. Journal of External Therapy of Traditional Chinese Medicine. 2018;27(02):20 - 21.<br><br>391. Jiang Pengjun, Hu Yanhong. Treatment of 41 Cases of Acute Cerebral Infarction with Comorbid Depression with the Brain - Awakening and Orifice - Opening Acupuncture Method Combined with Western Medicine. Traditional Chinese Medicine Research. 2018;31(05):53 - 55.<br><br>392. Fan Xiaoyan. Efficacy Observation on the Treatment of Post - stroke Depression with the Brain - Awakening and Orifice - Opening Acupuncture Method. Inner Mongolia Journal of Traditional Chinese Medicine. 2016;35(06):68 - 69.<br><br>393. Cui Lisheng. Efficacy Observation on the Treatment of 30 Cases of Post - stroke Depression with the Brain - Awakening and Orifice - Opening Acupuncture Method. Journal of Zhejiang Chinese Medical University. 2010;34(06):905 + 910.<br><br>394. Chen Xiangmei. Treatment of 180 Cases of Post - stroke Depression with the Brain - Awakening and Orifice - Opening Acupuncture Method. Chinese Medicine Modern Distance Education of China. 2013;11(22):71.<br><br>395. Kong Li, Shen Pengfei. Efficacy Observation on the Treatment of 180 Cases of Post - stroke Depression with the Brain - Awakening and Orifice - Opening Acupuncture Method. Journal of New Chinese Medicine. 2007;(10):27 - 28 + 8.<br><br>396. Jia Junli. Clinical Efficacy of the Brain - Awakening and Orifice - Opening Acupuncture Method in the Treatment of Post - stroke Depression. Clinical Journal of Traditional Chinese Medicine. 2015;7(21):90 - 91.<br><br>397. Shen Pengfei, Kong Li, Shi Xuemin. Clinical Research on the Treatment of Post

	- stroke Depression with the Brain - Awakening and Orifice - Opening Acupuncture Method. Chinese Acupuncture & Moxibustion. 2005;(01):15 - 17.<br><br>398. Gai Yingxia. Clinical Efficacy of the Brain - Awakening and Orifice - Opening Acupuncture Method in the Treatment of Post - stroke Depression. China Continuing Medical Education. 2019;11(10):158 - 160.<br><br>399. Wu Cong, Min Xirui. Clinical Research on the Treatment of Post - stroke Depression with the Brain - Awakening and Orifice - Opening Acupuncture Method. Medical Information. 2017;30(19):120 - 121.<br><br>400. Zhang Chuanwen, Min Xirui. Influence of the Brain - Awakening and Orifice - Opening Acupuncture Method Combined with Wuling Capsule on the Levels of Autophagy - related Proteins in Post - stroke Depression. Shanghai Journal of Acupuncture and Moxibustion. 2023;42(02):121 - 126.<br><br>401. Song Shuchang, Lu Zhi, Chen Hua, Wang Lichun. Treatment of 40 Cases of Post - stroke Depression with the Brain - Awakening and Mind - Regulating Acupuncture Method. Chinese Journal of Integrative Medicine on Cardio - Cerebrovascular Disease. 2013;11(07):824 - 825.<br><br>402. Wang Chongxin. Efficacy Observation on the Treatment of Post - stroke Depression with the Brain - Awakening and Governor - Vessel - Dredging Acupuncture Method. Liaoning Journal of Traditional Chinese Medicine. 2006;(05):601 - 602.<br><br>403. Ye Haimin, Zhang Peng, Lin Xuming, Yao Wen, Ren Zhen, Wu Qingming. Clinical Research on the Treatment of Post - stroke Depression by Acupuncture at "Siguanshu Points". Guiding Journal of Traditional Chinese Medicine and Pharmacy. 2016;22(07):60 - 62.<br><br>404. Wang Mingming, Wei Baoqiang, Cai Shengchao, Fu Yu, Tao Xiang. Clinical Observation on the Treatment of Post - stroke Depression by Acupuncture at "Baihui Five - needle" Combined with Blood - letting Therapy. Journal of Yunnan University of Traditional Chinese Medicine. 2017;40(05):73 - 76.<br><br>405. Cheng Junping, Zhang Guibo, Zhang Qian, et al. Clinical Research on the Treatment of Post - stroke Depression by Acupuncture at Different Groups of Acupoints. Shanghai Journal of Acupuncture and Moxibustion. 2016;35(12):1420 - 1422.<br><br>406. Sun Yuting, Zhu Peiqin, Sun Xingliang. Clinical Research on the Treatment of Post - stroke Depression by Acupuncture at Five - Heart Points Combined with Six - Mind - Soothing Points. Journal of Clinical Acupuncture and Moxibustion. 2022;38(11):21 - 24.<br><br>407. Jiang Feng, Li Changdu, Meng Xiaodan, Sun Yang, Zhao Xueqing. Clinical Efficacy Observation on the Treatment of Post - stroke Depression by Acupuncture at Five - Emotion Points. Journal of Clinical Acupuncture and Moxibustion. 2006;(12):25 - 26.<br><br>408. Feng Guifang, Li Jieyi, Wen Qingfen. Efficacy Observation on the Treatment of Post - stroke Depression by Acupuncture at the Back - shu Points of the Five Zang - organs. Shenzhen Journal of Integrated Traditional Chinese and Western Medicine. 2018;28(14):56 - 58.<br><br>409. Pan Yongqing. Treatment of 56 Cases of Post - stroke Depression by

Acupuncture at the Eight Confluent Points of the Eight Extraordinary Meridians. Shaanxi Journal of Traditional Chinese Medicine. 2007;(02):199 - 200.

410. Ge Qixue, Wu Yan. Treatment of 30 Cases of Depressive Disorder after Acute Cerebral Infarction with Acupuncture plus Oral Fluoxetine. Shaanxi Journal of Traditional Chinese Medicine. 2007;(06):720 - 723.

411. Wang Yiyan, Zhao Changfeng, Li Yan. Efficacy Observation on the Treatment of 76 Cases of Post - stroke Depression with Acupuncture plus Auricular - point Application. Journal of Changchun University of Chinese Medicine. 2009;25(04):549.

412. Ma Yanhong, Hao Jindong, Wang Meikang. Influence of Acupuncture at Sishencong and Siguanshu Points on P300 in Post - stroke Depression Patients. Chinese Journal of Rehabilitation Theory and Practice. 2007;(10):965 - 966.

413. Jiang Zhenya, He Lingna, Peng Liqun. Treatment of Post - stroke Depression by Acupuncture at Tiangu Eight - Formation. Chinese Acupuncture & Moxibustion. 2002;(01):31.

414. Wang Ming, Zhao Hui, Jia Guangyi, Zhang Lixia, Chai Yiting. Efficacy Analysis of Acupuncture at Head Acupoints in the Treatment of Post - stroke Depression. Doctor. 2019;4(15).

415. Zhang Xianbao, Wang Zhen, Zheng Zhijun, Liang Fajun. Influence of Acupuncture on tPA/BDNF Pathway - related Neuromodulatory Factors in Post - stroke Depression Patients. Science Technology and Engineering. 2021;21(15):6230 - 6235.

416. Li Zhenyu, Hua Qihai, Yan Xingzhou, Xu Lei. Influence of Acupuncture on the Incidence of Post - stroke Depression. Chinese Journal of General Practice. 2012;10(07):1111 - 1112.

417. Lin Ziling, Zhen Jun, Li Fanqiang. Influence of Acupuncture on the Rehabilitation Efficacy of Post - stroke Depression. China Practical Medicine. 2010;5(08):59 - 60.

418. Cai Zhaoyang. Influence of Acupuncture on the Neurological Function and Efficacy in Post - stroke Depression Patients. Fujian Journal of Traditional Chinese Medicine. 2018;49(05):26 - 27.

419. Zhai Tiejun, Luo Enli, Guo Shijie. Influence of Acupuncture - based Antidepressant Treatment on the Rehabilitation Efficacy of Post - stroke Depression. Journal of Clinical Acupuncture and Moxibustion. 2004;(05):6 - 8+1.

420. Zhang Jingsha, Geng Lianqi, Guo Yi. Study on the Efficacy of Acupuncture in Improving the Depressive State of Patients with Post - ischemic - stroke Depression. China Journal of Traditional Chinese Medicine and Pharmacy. 2021;36(03):1744 - 1747.

421. Zhao Yuanfei, Wang Xueyuan. Clinical Study on the Improvement of Heart Rate Variability in Post - stroke Depression Patients by Acupuncture. Journal of Shandong University of Traditional Chinese Medicine. 2013;37(05):408 - 409.

422. Jiao Daiyan, Deng Haipeng, Gu Heyan, Wang Lu, Zhang Ruochen. Research on the Prevention of Post - stroke Depression by Early Acupuncture Intervention in Stroke. Chinese Journal of Basic Medicine in Traditional Chinese Medicine. 2017;23(11):1605 - 1606 + 1621.

	423. Wei Guanwen. Clinical Observation on the Treatment of 30 Cases of Post - stroke Depression with Acupuncture. Liaoning Journal of Traditional Chinese Medicine. 2008;(10):1563 - 1564.<br><br>424. Ding Jianyu, Sun Xinghua, Zhao Jun. Treatment of 36 Cases of Post - stroke Depression with Acupuncture. Journal of Clinical Acupuncture and Moxibustion. 2004;(10):29.<br><br>425. Wei Xiaoyong. Treatment of 38 Cases of Post - stroke Depression with Acupuncture. Guangxi Journal of Traditional Chinese Medicine. 2006;(01):34 - 35.<br><br>426. Zhang Xiaofeng. Treatment of 56 Cases of Post - stroke Depression with Acupuncture. Henan Journal of Traditional Chinese Medicine. 2008;(08):75 - 76.<br><br>427. Li Jing. Treatment of 67 Cases of Post - stroke Depression with Acupuncture. Shanghai Journal of Acupuncture and Moxibustion. 2005;(06):27.<br><br>428. Zhang Jun, Ma Lingning. Treatment of 78 Cases of Post - stroke Depression with Acupuncture. Shanghai Journal of Acupuncture and Moxibustion. 2003;(11):33.<br><br>429. Wang Haijian. Clinical Observation on the Treatment of Post - stroke Depression with Acupuncture. Chinese Acupuncture & Moxibustion. 2003;(08):6 - 8.<br><br>430. Yang Chunming, Feng Caizhen. Clinical Observation on the Treatment of 43 Cases of Post - stroke Depression Patients with Acupuncture. Journal of Gansu College of Traditional Chinese Medicine. 2015;32(03):40 - 43.<br><br>431. Zhang Guibo, Yang Ling, Zhang Qian. Efficacy Observation on the Treatment of Post - stroke Depression with Acupuncture. Shanghai Journal of Acupuncture and Moxibustion. 2010;29(10):636 - 637.<br><br>432. Han Ping, Wang Zhenyu, Huang Yingshan, Luo Qinglu. Efficacy Observation on the Treatment of Post - stroke Depression with Acupuncture and Comparative Study on the Differences of ERP Component P300. Electronic Journal of Clinical Medical Literature. 2016;3(44):8753 - 8755.<br><br>433. Zhang Debin. Clinical Research on the Treatment of Post - stroke Depression with Acupuncture. Shaanxi Journal of Traditional Chinese Medicine. 2014;35(03):355.<br><br>434. Gu Wei. Clinical Observation on the Treatment of Post - stroke Depression with Acupuncture. Journal of Sichuan of Traditional Chinese Medicine. 2005;(10):102 - 103.<br><br>435. Xu Rui. Clinical Efficacy Observation on the Treatment of Post - stroke Depression with Acupuncture. Massage & Rehabilitation Medicine. 2018;9(21):21 - 23.<br><br>436. Ma Yunling, Kong Lingxin. Effect Observation and Nursing Experience of Acupuncture in the Treatment of Post - stroke Depression. Electronic Journal of Integrative Medicine on Cardio - Cerebrovascular Disease. 2019;7(33):127.<br><br>437. Du Huiping. Clinical Research on the Promotion of Neurological Function Rehabilitation in Post - stroke Depression by Acupuncture Treatment. Asian Pacific Traditional Medicine. 2017;13(04):103 - 104.

438. Liu Jianzhong, Huang Qianru, Yan Nianwen. Efficacy Observation on the Treatment of 30 Cases of Post - stroke Depression with Acupuncture and Its Influence on Serum 5 - HT. Hunan Journal of Traditional Chinese Medicine. 2017;33(03):79 - 81.

439. Zhang Yiwei, Liang Hui, Liu Jiajing, Yan Nianwen, Huang Yanping, Yang Yang. Clinical Observation on the Treatment of 41 Cases of Post - stroke Depression with Acupuncture. Fujian Journal of Traditional Chinese Medicine. 2021;52(05):14 - 15.

440. Yan Jiang, Dai Nan. Efficacy Observation on the Treatment of 85 Cases of Post - stroke Depression with Acupuncture. Journal of Sichuan of Traditional Chinese Medicine. 2016;34(06):201 - 203.

441. Zhao Haifeng, Zhou Zhengguo, Wang Baocheng. Clinical Controlled Study on the Treatment of Post - stroke Depression with Acupuncture. Journal of Practical Traditional Chinese Internal Medicine. 2012;26(03):77 - 78.

442. He Yuqin, Zheng Hongzhong, Chen Jinyong, Luo Lan. Clinical Research on the Treatment of Post - stroke Depression with Acupuncture. Journal of Practical Traditional Chinese Medicine. 2016;32(08):820 - 821.

443. Zhou Yafen, Chen Luni, Wang Chao, Chen Lifang, Fang Jianqiao. Single - center Randomized Controlled Trial of Acupuncture in the Treatment of Post - stroke Depression. Journal of Zhejiang Chinese Medical University. 2014;38(11):1322 - 1325.

444. Ruan Su, Liu Jianzhong, Huang Qianru, Zhang Yiwei. Research on the Treatment of Post - stroke Depression with Acupuncture and Its Correlation with Cytokines. Guangming Journal of Chinese Medicine. 2016;31(22):3366 - 3369.

445. Hai Ying, Zhang Hui. Treatment of 40 Cases of Post - stroke Depressive State with Acupuncture. Journal of Liaoning College of Traditional Chinese Medicine. 2003;(02):146.

446. Lu Ming, Sun Ming. Clinical Observation on the Treatment of 80 Cases of Post - stroke Depression with Acupuncture. Journal of Emergency in Traditional Chinese Medicine. 2005;(10):936 - 937.

447. Meng Fanhui, Meng Qingliang. Clinical Observation on the Treatment of 134 Cases of Post - stroke Depression with Acupuncture. The Frontier of Medicine. 2013;(32):353 - 353.

448. Kong Li, Shen Pengfei. Clinical Efficacy Analysis of Acupuncture in the Treatment of Post - stroke Depression. Liaoning Journal of Traditional Chinese Medicine. 2008;(01):117 - 118.

449. He Jun, Shen Pengfei. Clinical Efficacy Research on the Treatment of Post - stroke Depression with Acupuncture. Acupuncture Research. 2007;(01):58 - 61.

450. Yang Dingrong, Yan Wenqiang. Efficacy Observation on the Treatment of Post - stroke Depression with Acupuncture. Chinese Journal of Information on Traditional Chinese Medicine. 2007;(07):75 - 76.

451. Zhang Pei, Zhao Hong. Research on the Optimal Plan for the Treatment of Post - stroke Depression with Acupuncture. Hubei Journal of Traditional Chinese Medicine. 2010;32(04):26 - 27.

452. Buzaileihan·Remuti, Mailikaimu·Abudoukeliimu. Clinical Value of Acupuncture in the Treatment of Post - stroke Depression. Chinese and Foreign Women's Health Research. 2016;(04):171 + 188.

453. Yao Junpeng, Ma Xianjun. Clinical Efficacy Observation on the Treatment of Post - stroke Depression with Acupuncture. Continuing Medical Education. 2018;32(11):161 - 162.

454. Duan Xiaojing, Fan Junming. Clinical Observation on the Treatment of Post - stroke Depression with Acupuncture. Medical Innovation of China. 2012;9(03):107.

455. Liu Zhengfang, Zhang Jianquan, Shao Shujuan, Wang Shifeng. Efficacy Evaluation of Acupuncture in the Treatment of Post - stroke Depression. Journal of Sichuan of Traditional Chinese Medicine. 2013;31(02):111 - 113.

456. Dou Xintao, Wang Yulan, He Manman, Wang Dai, Wu Cunhu. Efficacy Evaluation of Acupuncture in the Treatment of Post - stroke Depression. Full - text Database of Medicine and Health in Chinese Science and Technology Journal Database. 2023;(1).

457. Liu Qingguo. Efficacy Observation on the Treatment of 65 Cases of Multi - infarct Depression with Acupuncture. Shanghai Journal of Acupuncture and Moxibustion. 2003;(02):10 - 11.

458. Guo Lian, Guo Ruiyou, Zhao Lixia, Su Li, Wang Li, Wang Caixia. Influence of Acupuncture Treatment on Plasma Leptin and Insulin - like Growth Factor - 1 in Elderly Post - stroke Depression Patients. International Journal of Traditional Chinese Medicine. 2012;34(1).

459. Wang Chaojin. Influence of Acupuncture Treatment on NIHSS Score and HAMD Score in Patients with Cerebral Hemorrhage Complicated with Depression. Psychologist. 2018;24(31):121 - 122.

460. Sha Tao, Gao Lili. Clinical Observation on the Treatment of Post - ischemic - stroke Depression with Acupuncture. Liaoning Journal of Traditional Chinese Medicine. 2022;49(05):150 - 153.

461. Sun Guochao. Research on the Clinical Effect of Acupuncture in the Treatment of Elderly Post - stroke Depression Patients. Chinese Journal of Geriatric Care. 2013;11(02):75 + 77.

462. Zeng Shuai. Clinical Efficacy Observation on the Treatment of Post - stroke Emotional Disorders with Acupuncture. The Frontier of Medicine. 2014;(2):365 - 366.

463. Zhu Wei, Guo Xiaobin. Acupuncture Treatment for Post - stroke Depression. Chinese Journal of Clinical Rehabilitation. 2003;(22):3145.

464. Li Hongjie, Zhong Baoliang, Fan Yinping, Hu Hongtao. Acupuncture in the Treatment of Post - stroke Depression: A Randomized Controlled Study. Chinese Acupuncture & Moxibustion. 2011;31(01):3 - 6.

465. Fu Lei, Liu Wei'ai, Li Xiangrong, et al. Efficacy Observation on the Treatment of 20 Cases of Post - stroke Depression with Acupuncture. Hunan Journal of Traditional Chinese Medicine. 2013;29(05):75 - 77.

466. Fu Lei, Liu Wei'ai, Li Xiangrong, et al. Efficacy Observation on the Treatment of 40 Cases of Post - stroke Depression with Acupuncture. Hunan Journal of Traditional Chinese Medicine. 2013;29(09):84 - 86.

467. Li Shaokang, Zhou Dehua, Wang Han, Liao Weidong. Intervention Effect of Acupuncture in the Treatment of Post - stroke Depression and on the Neurological Function Rehabilitation of Patients. China Medicine and Pharmacy. 2015;5(07):75 - 77.

468. Lü Xiaohua, Zhang Haijun. Acupuncture Treatment for Post - stroke Depression Insomnia. Medical Aesthetics and Cosmetology (Mid - month Edition). 2014;(4):442 - 442.

469. Wang Yin, Zhao Zhifu, Wu Yu, et al. Clinical Efficacy Evaluation of Acupuncture in the Treatment of Post - stroke Depression Insomnia. Chinese Acupuncture & Moxibustion. 2004;(09):13 - 16.

470. Jing Suqing, Chen Huan, Zhang Xiyu, Huang Zongju. Influence of Acupuncture in the Treatment of Post - stroke Depression on Neurological Function Rehabilitation. Modern Medicine & Health. 2018;34(16):2559 - 2561.

471. Sun Xiaodong, Yang Ning, Che Wensheng. Efficacy of Acupuncture in the Treatment of Post - stroke Depression Patients and Its Influence on the Neurotrophic State and Monoamine Neurotransmitters. World Chinese Medicine. 2018;13(09):2285 - 2287 + 2291.

472. Zhang Jingfeng. Effect Analysis of Acupuncture in the Treatment of Post - stroke Depressive State. Chinese Journal of Clinical Rehabilitation. 2004;(25):5372.

473. Li Dalei, Zhao Canwei. Research on the Efficacy of Acupuncture in the Treatment of Post - stroke Depression. Shaanxi Journal of Traditional Chinese Medicine. 2020;41(10):1489 - 1491.

474. Chu Yunjie, Wang Chengyuan, Zhang Hong. Clinical Observation on the Treatment of 72 Cases of Post - stroke Depression with Acupuncture. Chinese Journal of Gerontology. 2007;(17):1720 - 1721.

475. Ran Maodong, Guo Yunping. Clinical Observation on the Treatment of 80 cases of Post - stroke Depression with Acupuncture. Liaoning Journal of Traditional Chinese Medicine. 2006;(10):1332.

476. Wang Hairong. Treatment of 140 Cases of Post - stroke Depression with Acupuncture. Journal of Emergency in Traditional Chinese Medicine. 2002;(04):303 - 304.

477. Liu Yunxia. Clinical Effect Observation of Acupuncture in the Treatment of Post - stroke Depression. Inner Mongolia Journal of Traditional Chinese Medicine. 2021;40(01):118 - 119.

478. Yang Xiaohui, Ouyang Basi. Clinical Observation on the Treatment of Post - stroke Depression with Acupuncture. Journal of Liaoning University of Traditional Chinese Medicine. 2010;12(01):159 - 161.

479. Zhao Hui, Zhao Lijie, Guo Dan. Discussion on the Effect of Acupuncture in the Treatment of Post - stroke Depression. Health Care Guide. 2021;(30):110 - 111.

480. Zhao Lihong. Efficacy Analysis of Acupuncture in the Treatment of Post -

stroke Depression. Chinese Journal of Gerontology. 2004;(12):1207 - 1208.

481. Chen Zhikang, Yuan Song, Li Haifeng, Wang Junhua, Li Mingfen. Clinical Observation on the Treatment of Post - stroke Depression with Acupuncture. Massage & Rehabilitation Medicine. 2018;9(05):28 - 30.

482. Sun Wenli. Analysis of the Effect of Acupuncture in the Treatment of Post - stroke Depression. Contemporary Medicine Forum. 2021;19(1).

483. Zhang Lu. Efficacy Observation of Acupuncture in the Treatment of Post - stroke Depression. Chinese Journal of Traditional Chinese Medicine and Pharmacy. 2020;27(03):413 - 414.

484. Song Shuchang, Lu Zhi, Wang Runyun, Chen Hua. Intervention Effect of Acupuncture in the Treatment of Post - stroke Depression and on the Neurological Function Rehabilitation of Patients. Journal of Clinical Acupuncture and Moxibustion. 2014;30(10):7 - 9.

485. Zhu Qiyu. Intervention Effect of Acupuncture in the Treatment of Post - stroke Depression and on the Neurological Function Rehabilitation of Patients. China Foreign Medical Treatment. 2015;34(13):170 - 171.

486. Song Shuchang, Lu Zhi, Wang Runyun, Chen Hua, Wang Xiumei, Kang Weige. Intervention Effect and Safety Evaluation of the Thought of Treating the Mind by Acupuncture on Post - stroke Depression. Journal of Sichuan of Traditional Chinese Medicine. 2015;33(05):157 - 159.

487. Tan Qiurong, Wang Yang, Yao Mingsong. Efficacy Observation of Acupuncture in the Treatment of Post - stroke Depression during the Rehabilitation Period. Chinese Folk Remedies. 2017;25(02):23.

488. Gong Rui. Clinical Effect Analysis of Acupuncture Combined with Fluoxetine in the Treatment of Post - stroke Depression. Contemporary Medicine Forum. 2021;19(17).

489. Yang Libai, Liu Yongtao, Liu Meizhang, Li Zhenan, Liu Qun. Influence of Acupuncture Therapy on the Improvement of Depression in Stroke Patients during the Rehabilitation Period. Journal of Hebei University of Chinese Medicine. 2021;36(01):46 - 49.

490. Gong Zehui, Xia Yingdan, Yuan Jianrong, et al. Efficacy Observation of Acupuncture Therapy Combined with Electrical Stimulation in the Treatment of Elderly Post - stroke Depression Patients. Chinese Journal of Cardiovascular Rehabilitation Medicine. 2019;28(04):498 - 501.

491. Shang Lei. Clinical Observation on the Treatment of Post - stroke Depression with Acupuncture Combined with Auricular - point Application. Chinese Folk Remedies. 2023;31(03):51 - 54.

492. Wu Jiayong, Liu Chengyan, Huang Guirong, Wang Linlin. Clinical Observation on the Treatment of Post - stroke Depression by Acupuncture at Baihui and Siguanshu Points. Chinese Medicine Modern Distance Education of China. 2019;17(18):90 - 92.

493. Zhao Hong, Zhao Wenli. Clinical Research on the Treatment of Post - stroke Depression with Acupuncture at Baihui Point. Chinese Archives of Traditional Chinese Medicine. 2007;(02):275 - 277.

	494. Jiao Haifeng, Li Junfeng, Zhang Liang. Efficacy Observation of Acupuncture at Baiyin Point in the Treatment of Post - stroke Depression. World Latest Medicine Information. 2016;16(19):151.<br><br>495. Ding Zhou, Yu Xiaogang. Clinical Analysis of Treating Post - stroke Depression mainly by Acupuncture at Governor Vessel Acupoints. Journal of Beijing University of Chinese Medicine (Clinical Medicine Edition). 2003;(03):31 - 33.<br><br>496. Qin Na, Hu Guoqiang, Cao Rong, He Xuexin. Clinical Effect of Acupuncture at Shentang and Naohu Points in the Treatment of Post - stroke Depression. China Medical Herald. 2022;19(11):160 - 163.<br><br>497. Ye Jian. Clinical Effect Analysis of Acupuncture at Shentang and Naohu Points in the Treatment of Post - stroke Depression. Diet Science. 2022;(10):73 - 75.<br><br>498. Wang Yanjun, Li Hongkun, Wang Yebo, et al. Clinical Research on the Treatment of Post - stroke Depression with Acupuncture Combined with Rehabilitation Training. Hebei Journal of Traditional Chinese Medicine. 2019;41(11):1725 - 1728.<br><br>499. Li Yanli, Yang Zufu, Xu Jimin, et al. Treatment of Moderate Post - stroke Depression with Acupuncture Combined with Flupentixol and Melitracen: A Randomized Controlled Study. Chinese Journal of Rehabilitation Medicine. 2021;36(07):810 - 815.<br><br>500. Wu Ming, Chen Zelin, Zhao Mingliang. Efficacy Observation on the Treatment of 40 Cases of Post - stroke Depression with Acupuncture Combined with Fluoxetine. Shanghai Journal of Traditional Chinese Medicine. 2010;44(05):49 - 50.<br><br>501. Wang Feng, Lou Xiaomin, Xia Luomin, Qian Chen, Jiang Xinping. Clinical Observation on the Treatment of Post - ischemic - stroke Depression with Acupuncture Combined with Point Injection. Shanghai Journal of Acupuncture and Moxibustion. 2016;35(08):942 - 944.<br><br>502. Pu Fang, Li Peifang, Sun Peiyang. Influence of Acupuncture Combined with Medication on the Symptoms of Post - stroke Depression Patients and Serum Brain - derived Neurotrophic Factor. Liaoning Journal of Traditional Chinese Medicine. 2023;50(04):168 - 171.<br><br>503. Cui Youxiang, Hu Fangmei, Wang Hongliang, Liu Guohua, Duan Hongbo, Yuan Chunxiao. Clinical Effect Observation of Acupuncture Combined with Medication in the Treatment of Post - stroke Depression. Medical Innovation of China. 2019;16(14):79 - 82.<br><br>504. Li Xiaoli. Efficacy Observation of Acupuncture Combined with Medication in the Treatment of Depression after Cerebral Infarction. Chinese Medicine Modern Distance Education of China. 2015;13(24):19 - 20.<br><br>505. Zhang Ruochen, Jiao Daiyan, Deng Haipeng, Gu Heyan, Wang Lu. Clinical Research on the Treatment of Post - stroke Depression with Acupuncture Combined with Routine Western Medicine Therapy. International Journal of Traditional Chinese Medicine. 2017;39(12).<br><br>506. Sun Zhe, Ai Zongyao, Li Zhengrong, Li Xue, Li Danping. Efficacy Observation of Acupuncture Combined with Compound Traditional Chinese Medicine in the Treatment of Post - stroke Depression and Its Influence on the Levels of Serum 5 - HT, NE and BDNF in Patients. Shanghai Journal of Acupuncture and Moxibustion.

	2022;41(04):342 - 347.<br><br>507. Wang Ran, Yang Hualin. Influence of Acupuncture Combined with the Traditional Chinese Medicine Compound Yuleshu on the Depressive Symptoms and Neurological Function Rehabilitation of Post - stroke Depression Patients. Journal of Liaoning University of Traditional Chinese Medicine. 2018;20(09):174 - 177.<br><br>508. Wang Junyi. Clinical Research on the Treatment of Post - stroke Depression with Acupuncture Combined with Occupational Therapy. Journal of New Chinese Medicine. 2022;54(24):162 - 165.<br><br>509. Zhang Jianzhi, Wang Meiqin, Zhao Weiguo. Treatment of 89 Cases of Early Post - stroke Depression with Acupuncture Combined with Routine Medication. Hebei Journal of Traditional Chinese Medicine. 2009;31(11):1688 - 1689.<br><br>510. Li Jinming. Influence of Acupuncture Combined with Rehabilitation Training on the Cognitive Function of Post - stroke Depression Patients. Chinese Journal of Practical Nervous Diseases. 2018;21(16):1820 - 1826.<br><br>511. Xia Junhui, Xu Cuie, Xia Wenguang, Zheng Chanjuan. Clinical Observation on the Treatment of Post - stroke Depression with Acupuncture Combined with Rehabilitation Training. Shanghai Journal of Acupuncture and Moxibustion. 2015;34(08):724 - 727.<br><br>512. Guo Lijuan, Gao Hanning, Zhang Qiufeng, Deng Shuqin, Li Jun. Influence of Acupuncture Combined with Antidepressants on the Activities of Daily Living and Cognitive Function of Patients with Depression after Cerebral Infarction. International Journal of Psychiatry. 2018;45(02):337 - 339 + 356.<br><br>513. Bi Haiyang, Yu Nannan, Han Li. Influence of Acupuncture Combined with Press - needles at the Back - shu Points of the Five Zang - organs on the Efficacy and Cognitive Function of Patients with Post - ischemic - stroke Depression. Journal of Guangzhou University of Chinese Medicine. 2022;39(11):2558 - 2563.<br><br>514. Liu Yongkuo, Qin Lina, Cai Yuanbin, Wang Ziwei. Efficacy of Acupuncture Combined with Flupentixol and Melitracen Tablets in the Treatment of Post - stroke Depression and Its Influence on the Levels of Monoamine Neurotransmitters and Neurotrophic Factors in the Blood of Patients. Hebei Journal of Traditional Chinese Medicine. 2022;44(12):2059 - 2062 + 2086.<br><br>515. Guo Aisong, Li Aihong, Gu Yihuang, Jin Hongzhu. Influence of Acupuncture Combined with Fluoxetine on the Efficacy of Post - stroke Depression and the Function of the HPA Axis. Medical Journal of Communications. 2007;(05):496 - 497 + 500.<br><br>516. Xu Gang. Randomized Parallel - controlled Study on the Treatment of Post - stroke Depression with Acupuncture Combined with Fluoxetine. Journal of Practical Traditional Chinese Internal Medicine. 2014;28(11):158 - 160.<br><br>517. Gu Yongsheng, Song Jinggui, Gu Renjun, Zhang Zhaohui, Mu Junlin. Influence of Acupuncture Combined with Fluoxetine on the Depressive Mood and Cognitive Function of Post - stroke Depression Patients. Medical Journal of Chinese People's Health. 2013;25(15):1 - 3.<br><br>518. Liu Jinzhao, An Quanyu. Treatment of 52 Cases of Post - stroke Depression in the Elderly with Acupuncture Combined with Fluoxetine. Henan Journal of Traditional Chinese Medicine. 2014;34(09):1700 - 1701.

	519. Qiao Shiguang, Hu Nan. Clinical Observation on the Treatment of Post - stroke Depression of Liver - depression and Spleen - deficiency Type with Acupuncture Combined with Heat - sensitive Moxibustion. Chinese Medicine Modern Distance Education of China. 2023;21(15):125 - 127.<br><br>520. Wang Weiwei, Huo Jinlian, Tian Yongqing, Liu Yong. Efficacy Analysis of Acupuncture Combined with Fluoxetine in the Treatment of Cerebral Infarction with Comorbid Depression. International Journal of Psychiatry. 2016;43(01):113 - 115.<br><br>521. Yu Wenya. Treatment of 87 Cases of Post - stroke Depression with Acupuncture Combined with Fluoxetine Hydrochloride Capsules. Hebei Journal of Traditional Chinese Medicine. 2010;32(04):567 - 568.<br><br>522. Guo Yang, Xu Meng. Effect of Acupuncture Combined with Mirtazapine on Post - stroke Depression Patients. Medical Journal of Chinese People's Health. 2021;33(21):74 - 76.<br><br>523. Su Yue, Xiao Hongbo, Li Mengyuan, Han Chengcheng, Ge Miaomiao. Clinical Research on the Treatment of Mild - to - Moderate Post - stroke Depression with Acupuncture Combined with Transcranial Direct - current Stimulation. Journal of Clinical Traditional Chinese Medicine. 2023;35(11):2222 - 2226.<br><br>524. Zhang Lin, Zhong Yan, Quan Shulin, et al. Acupuncture Combined with Auricular - point Application in the Treatment of Post - stroke Depression: A Randomized Controlled Study. Chinese Acupuncture & Moxibustion. 2017;37(06):581 - 585.<br><br>525. Zhang Li, Wu Haiyan, Ma Li, Cao Meng, Zhang Shouyu. Clinical Research on the Treatment of 43 Cases of Post - stroke Depression with Acupuncture Combined with Escitalopram Oxalate Tablets. Jiangsu Journal of Traditional Chinese Medicine. 2019;51(01):60 - 62.<br><br>526. Zhao Yingying, Song Qi, Wu Longhai, Dong Meichen. Clinical Research on the Effect of Acupuncture Combined with Medication on the Emotional Disorders of Post - stroke Depression Patients. Heilongjiang Medicine. 2021;34(01):206 - 207.<br><br>527. Tang Yongxiang, Liao Muxi, Wang Xiaoling, He Jun. Clinical Observation on the Treatment of Post - stroke Depression mainly by Acupuncture at Back - shu Points. Journal of Sichuan of Traditional Chinese Medicine. 2011;29(08):113 - 114.<br><br>528. Liu Juntao, Huang Yan, Liu Shanshan, Liu Shuang, Mu Yan, Du Ruosang. Efficacy Observation on the Treatment of Post - stroke Depression with Acupuncture at the Five Back - heart Points. World Journal of Integrated Traditional and Western Medicine. 2021;16(06):990 - 994.<br><br>529. Qin Na, Hu Guoqiang, 曹荣, Hu Yonghong, He Xuexin, Li Lichun. Efficacy Evaluation of Acupuncture at Naohu and Shentang Points in the Treatment of Post - stroke Depression. Modern Journal of Integrated Traditional Chinese and Western Medicine. 2022;31(07):918 - 921.<br><br>530. Wang Guihua, Wang Jinding. Clinical Observation on the Treatment of 56 Cases of Post - stroke Depression with the Combination of Abdominal Moxibustion and Acupuncture. Health Way. 2018;0(7).<br><br>531. Liu Lanlan, Huang Bing, 耿翊宁, Liu Jiaci, Song Manping. Influence of Acupuncture at Tanzhong Point on the Psychological State and Neurological Function

of Post - stroke Depression Patients. Western Journal of Traditional Chinese Medicine. 2021;34(04):131 - 134.

532. Yu Xueping, Yang Caijia, Zhang Jieyu. Clinical Efficacy Observation of Acupuncture at Tanzhong Point in the Treatment of Post - stroke Depression. Journal of Clinical Acupuncture and Moxibustion. 2016;32(08):59 - 62.

533. Song Shuchang, Lu Zhi, Wang Runyun. Clinical Research on the Treatment of Post - stroke Depression with the Method of Acupuncture at Differentially - selected Points. Journal of Emergency in Traditional Chinese Medicine. 2014;24(08):1453 - 1454 + 1478.

534. Wu Jiaping. Clinical Observation on the Treatment of 150 Cases of Post - stroke Depression with Syndrome - differentiated Acupuncture. Acupuncture Research. 2010;35(04):303 - 306.

535. He Yuqin, Wan Saiying, Zheng Hongzhong, Chen Jinyong. Clinical Research on the Treatment of Post - stroke Depression with Syndrome - differentiated Acupuncture. Journal of New Chinese Medicine. 2015;47(08):237 - 238.

536. Cao Renjun. Efficacy Research on the Treatment of Post - stroke Depression with Acupuncture Combined with Traditional Chinese Medicine Music Therapy. Family Medicine. Medical Selection. 2020;(2):340 - 341.

537. Zhan Daowei, Luo Kaitao, Mao Liyu, et al. Clinical Research on the Treatment of Post - stroke Depression with Acupuncture Combined with Rehabilitation Training. Shanghai Journal of Acupuncture and Moxibustion. 2016;35(07):792 - 795.

538. Ma Yanhui, Bi Haiyang, Ma Lin, et al. Efficacy Observation of Acupuncture Combined with Press - needles in the Treatment of Mild - to - Moderate Post - stroke Depression. Shanghai Journal of Acupuncture and Moxibustion. 2019;38(02):174 - 177.

539. Wu Xuemei, Zhang Bo. Efficacy Observation on the Treatment of 40 Cases of Post - stroke Depressive Disorder with Acupuncture Combined with Oral Fluoxetine. Journal of Difficult and Complicated Cases. 2008;(06):357 - 358.

540. Wang Fei, Pan Wei, Li Yunfang. Efficacy Observation of Acupuncture combined with auricular electroacupuncture on post-stroke depression and its influence on quality of life. Shanghai Journal of Acupuncture and Moxibustion. 2016; 35 (9) : 1033-1035.

541. Duan Bo, Guo Bei. Efficacy Observation of Acupuncture Combined with Electro - acupuncture at Auricular Points in the Treatment of Post - stroke Depression and Its Influence on the Quality of Life. Chinese Folk Remedies. 2018;26(10):39 - 40.

542. Tian Weiwei, Hu Yonghe, Chen Xian, Chang Hongsheng, Ma Yinyan, Liao Qi. Efficacy Observation of Acupuncture Combined with Sertraline on the Depression and Neurological Function Recovery of Post - stroke Depression Patients. Medical Journal of National Defending Forces in Southwest China. 2011;21(05):525 - 526.

543. Tang Jun, Yang Qin, Zhang Guoyong. Clinical Effect Analysis of Acupuncture at Ghost Points Combined with Modified Wendan Decoction in the Treatment of Post - stroke Depression. The World of Health. 2023;(23):131 - 132.

544. Xue Aiguo. Treatment of 31 Cases of Depressive Disorder in the Early Recovery Stage of Cerebral Infarction with Acupuncture. Shaanxi Journal of Traditional

Chinese Medicine. 2002;23(8):731 - 732.

545. Chen Jing, Zeng Chao, Li Hui, Tang Qiang, Zhu Luwen. Influence of Acupuncture - rehabilitation Therapy on the Cognitive and Neurological Function Recovery of Post - stroke Depression Patients. Acta Chinese Medicine and Pharmacology. 2021;49(6).

546. Dong Zhuoyuan, Dai Qibin, Wang Fenglei, et al. Comparison of the Efficacy of Acupuncture and Medication in the Treatment of Post - stroke Depression. Chinese Journal of Clinical Rehabilitation. 2003;(25):3516.

547. Nie Rongrong, Mo Biwen, Zeng Weixing, et al. Influence of Acupuncture on the Levels of Serum 5 - HT, NE and BDNF in Post - stroke Depression Patients. Medical Journal of West China. 2017;29(06):808 - 812 + 816.

548. Ni Jinglin. Influence of Acupuncture on the Effect and Neurological Function of Post - stroke Depression Patients. Health Today. 2021;(17):111 - 112.

549. Niu Yinglin, Liu Chengmei, Wang Xiaodan, Wang Lina, Liu Yan, Feng Xiaodong. Influence of Combined Acupuncture and Moxibustion on the Heart Rate Variability of Post - stroke Depression Patients. Chinese Journal of Rehabilitation Theory and Practice. 2015;21(02):196 - 198.

550. Yao Yonggang. Clinical Observation on the Treatment of 64 Cases of Post - stroke Depression with Acupuncture. Clinical Journal of Traditional Chinese Medicine. 2017;9(26):86 - 87.

551. Zhang Jie, Zheng Wei. Clinical Efficacy of Acupuncture in the Treatment of Post - stroke Depression. Inner Mongolia Journal of Traditional Chinese Medicine. 2020;39(11):105 - 106.

552. Zhou Minya, Wu Liuxiu. Clinical Efficacy and Safety Evaluation of Acupuncture in the Treatment of Post - stroke Depression. Chinese Archives of Traditional Chinese Medicine. 2016;34(02):502 - 504.

553. Wang Jing, Jin Dong, Wang Ying. Clinical Efficacy and Safety Analysis of Acupuncture in the Treatment of Post - stroke Depression. Inner Mongolia Journal of Traditional Chinese Medicine. 2018;37(03):57 - 58.

554. Cui Huaen. Clinical Observation on the Treatment of Post - stroke Depression with Acupuncture. Journal of Practical Traditional Chinese Medicine. 2017;33(06):719 - 720.

555. Xiao Shunqiong, Li Guojun. Clinical Observation on the Treatment of Post - stroke Depression with Acupuncture. Journal of Practical Traditional Chinese Medicine. 2014;30(02):134 - 136.

556. Gao Jia. Efficacy Evaluation of Acupuncture in the Treatment of Post - stroke Depression. Asian Pacific Traditional Medicine. 2013;9(11):74 - 75.

557. Zhai Chunmei. Clinical Effect Observation of Acupuncture in the Treatment of Post - stroke Depression. Diabetes World. 2021;18(3):101 - 102.

558. Liu Jianfeng. Clinical Efficacy and Safety of Acupuncture in the Treatment of Post - stroke Depression. Inner Mongolia Journal of Traditional Chinese Medicine. 2017;36(18):101 - 102.

	559. Qin Yanyan. Research on the Effect of Acupuncture in the Treatment of Post - stroke Depression. Family Life Guide (Second Half Month Edition). 2020;(11).<br><br>560. Zhao Yuanchen, Li Hongyan. Clinical Observation on the Treatment of 23 Cases of Post - stroke Depression with Acupuncture. Journal of Clinical Acupuncture and Moxibustion. 2006;(07):19 - 20.<br><br>561. Nie Rongrong, Mo Biwen, Wen Hui, Peng Hongxia, Zhang Qiaoyun, Qi Li. Influence of Acupuncture Treatment on the Quality of Life of Post - stroke Depression Patients. Clinical Misdiagnosis & Mistherapy. 2017;30(11):93 - 96.<br><br>562. Dai Junmei, Zhang Bo, Dong Wei. Research on the Influence of Acupuncture Treatment on the Neurological Function of Post - stroke Depression Patients. Journal of Hunan University of Chinese Medicine. 2020;(S01).<br><br>563. Duan Yanli. Influence of Acupuncture Treatment on the Neurological Function of Post - stroke Depression Patients. Health Must - Read. 2020;(18):152.<br><br>564. Sui Ruhua, Zhang Lei, Sui Rubo. Clinical Observation on the Treatment of 72 Cases of Post - acute - stroke Depression with Acupuncture. Chinese Journal of Ethnomedicine and Ethnopharmacy. 2009;18(11):94 - 95.<br><br>565. Wang Yajing. Clinical Value Analysis of Acupuncture in the Treatment of Post - acute - stroke Depression. Health Must - Read. 2020;(27):71.<br><br>566. Chu Liuci, Cheng Weiping. Clinical Analysis of Acupuncture in the Treatment of Post - acute - stroke Depression. Heilongjiang Science. 2017;8(06):164 - 165.<br><br>567. Zhou Jisong, Liu Baicheng. Clinical Effect Observation of Acupuncture in the Treatment of Post - acute - stroke Depression. Health Care Guide. 2021;(50):106 - 108.<br><br>568. Jiang Xinling, Ma Xiaohua. Efficacy of Acupuncture in the Treatment of Stroke with Comorbid Depression and Its Value for the Neurological Function Rehabilitation of Patients. Health Must - Read. 2020;(31):261 - 262.<br><br>569. Li Juntao, Yang Yuhua. Efficacy Observation of Acupuncture in the Treatment of Stroke with Comorbid Depression and Its Intervention Effect on the Neurological Function Rehabilitation of Patients. Hunan Journal of Traditional Chinese Medicine. 2019;35(06):67 - 68.<br><br>570. Gao Li, Sun Xiaolian, Fu Lina, Fan Wentao. Acupuncture Treatment for Post - stroke Depression. Jilin Journal of Traditional Chinese Medicine. 2016;36(07):743 - 745.<br><br>571. Yan Changchang, Su Shaoyong, Tang Genka, Yu Zhanli. Research on the Efficacy of Acupuncture in the Treatment of Post - stroke Depression. Shaanxi Journal of Traditional Chinese Medicine. 2018;39(01):122 - 124.<br><br>572. Sun Ting. Efficacy and Safety of Acupuncture in the Treatment of Post - stroke Depression. Dajia Health (Middle Edition). 2017;11(5):17 - 18.<br><br>573. Nie Rongrong, Huang Chunhua. Evaluation of the Efficacy and Safety of Acupuncture in the Treatment of Post - stroke Depression. Chinese Acupuncture & Moxibustion. 2013;33(06):490 - 494.<br><br>574. Li Peng, He Liqing. Efficacy Analysis of Acupuncture in the Treatment of Post -

stroke Depression. Scientific Regimen. 2021;24(3):175.

575. Zhao Xiaobing. Efficacy Observation of Acupuncture in the Treatment of Post - stroke Depression. China Healthcare Innovation. 2012;7(06):38 - 39.

576. Shen Lili. Research on the Efficacy of Acupuncture in the Treatment of Post - stroke Depression. Famous Doctor. 2018;(10):35.

577. Xuan Hongwei. Clinical Analysis of Acupuncture in the Treatment of Post - stroke Depression. Shenzhen Journal of Integrated Traditional Chinese and Western Medicine. 2017;27(18):54 - 55.

578. Bao Xingang. Clinical Efficacy of Acupuncture in the Treatment of Post - stroke Depression Patients and Observation on Its Influence on the Neurological Function of Patients. Chinese and Foreign Women's Health Research. 2022;(7).

579. Zhao Qisheng. Effect of Acupuncture in the Treatment of Post - stroke Depression Patients and Its Influence on the Neurological Function of Patients. Chinese and Foreign Medical Research. 2019;17(14):164 - 165.

580. Yin Shirong, Chen Ruijiao. Efficacy of Acupuncture in the Treatment of Post - stroke Depression Patients and Analysis of Its Influence on the Neurological Function of Patients. Psychologist. 2018;24(10):96 - 97.

581. Sun Shan. Efficacy of Acupuncture in the Treatment of Post - stroke Depression Patients and Its Influence on the Neurological Function of Patients. Diabetes World. 2020;17(4):53.

582. Meng Jianbo. Efficacy of Acupuncture in the Treatment of Post - stroke Depression Patients and Its Influence on the Neurological Function of Patients. Smart Healthcare. 2023;9(10):140 - 143.

583. Li Shaohong. Efficacy of Acupuncture in the Treatment of Post - stroke Depression Patients and Its Influence on the Neurological Function of Patients. Northern Pharmacology. 2018;15(11):134 - 135.

584. Yong Hong. Efficacy of Acupuncture in the Treatment of Post - stroke Depression Patients and Its Influence on the Neurological Function of Patients. Full - text Database of Medicine and Health in Chinese Science and Technology Journal Database. 2021;(5).

585. Qi Linjing, Su Tongsheng, Song Rui, Xie Yu. Efficacy of Acupuncture in the Treatment of Post - stroke Depression Patients and Its Influence on the Neurological Function of Patients. Guizhou Medical Journal. 2019;43(10):1576 - 1578.

586. Liu Laiming. Efficacy of Acupuncture in the Treatment of Post - stroke Depression Patients and Analysis of Its Influence on the Neurological Function of Patients. Famous Doctor. 2021;(08):52 - 53.

587. Xia Shenghua. Research on the Efficacy of Acupuncture in the Treatment of Post - stroke Depression Patients and Its Influence on the Neurological Function of Patients. Healthful Friend. 2021;(16):43 - 44.

588. Yao Jin. Effectiveness of Acupuncture in the Treatment of Post - stroke Depression and Evaluation of Neurological Function Indexes of Patients. Health Must - Read. 2020;(25):240.

589. Zhao Jie. Efficacy Observation of Acupuncture in the Treatment of Post - stroke Depression. Inner Mongolia Journal of Traditional Chinese Medicine. 2019;38(07):97 - 98.

590. Shuai Yi. Efficacy of Acupuncture in the Treatment of Depression after Cerebrovascular Accident and Its Influence on the Neurological Function of Patients. Chinese Journal of Convalescent Medicine. 2019;28(2).

591. Zhu Fengying. Research on the Influence of Acupuncture - warming - and - dredging Method on the Neurological Function of Post - stroke Depression Patients. International Journal of Traditional Chinese Medicine Research. 2022;2(2).

592. Maidanjiang·Maimaiti. Observation on the Application Effect of Acupuncture Therapy in the Treatment of Post - stroke Depression. Citation Database of Medicine and Health in Chinese Science and Technology Journal Database. 2021;(7).

593. Tang Yongchun, Zhou Xiyan, Gao Lili. Research on the Clinical Efficacy of Acupuncture Therapy on Post - acute - cerebral - infarction Depression Patients and Its Influence on the Serum VEGF Level. Contemporary Medicine. 2017;23(12):25 - 27.

594. Guo Dan, Zhao Lijie, Zhao Hui. Research on the Improvement of the Condition of Post - stroke Depression Patients by Acupuncture Therapy. Health Care Guide. 2021;(30):113 - 114.

595. Xing Laili. Efficacy Observation of Acupuncture Combined with Blood - letting in the Treatment of Post - stroke Depression. Modern Journal of Integrated Traditional Chinese and Western Medicine. 2014;23(22):2485 - 2487.

596. Wang Weihua, Zhang Qian, Wei Yan. Clinical Efficacy Observation of Acupuncture Combined with Medication in the Treatment of Post - stroke Depression. Chinese Folk Remedies. 2011;19(07):50 - 51.

597. Jiang Lei, Zhao Jun. Clinical Research on Acupuncture Combined with Medication in the Treatment of Post - stroke Depression. Journal of Clinical Acupuncture and Moxibustion. 2011;27(11):15 - 17.

598. Wang Cheng, Xiang Yunxia, Jiang Qiao. Efficacy of Acupuncture Combined with Modified Buyang Huanwu Decoction in the Treatment of PSD and Its Influence on the Results of HAMD, SDS and BECK Scales. Hebei Medicine. 2018;24(04):701 - 704.

599. Liu Guihua, Chen Guilin, Xia Jiangyuan. Effect of Acupuncture Combined with Feedback - type Health Education in the Treatment of Post - stroke Depression and Its Influence on the Depressive State. Journal of Cardiovascular Surgery (Electronic Edition). 2020;9(1).

600. Xu Changmin, Zhang Xiaojian, Liu Hongwei, Liu Shuqing, Yuan Chen. Effectiveness of Acupuncture Combined with Paroxetine in the Treatment of Post - stroke Depression Patients and Its Influence on the Cognitive Function of Patients. Journal of Preventive Medicine of Chinese People's Liberation Army. 2019;37(10):46 - 47.

601. Tian Huijun, Li Huijun, Zhao Zili, Liu Xiaoyang, Wang Yanfang. Influence of Acupuncture - moxibustion Combined with Flupentixol and Melitracen on Negative Emotions and Serum 5 - Hydroxytryptamine Level in Post - stroke Depression Patients. Shanxi Medical Journal. 2019;48(22):2731 - 2733.

	602. Zhang Ying. Randomized Parallel - controlled Study on the Treatment of Post - stroke Depression with Acupuncture - moxibustion Combined with Fluoxetine Dispersible Tablets. Journal of Practical Traditional Chinese Internal Medicine. 2014;28(02):123 - 124.<br><br>603. Li Chuanyou, Li Yeyu, Ma Hui, Wei Chunling, Sheng Lei, Gu Zhaojun. Clinical Study of 90 Cases on the Influence of Acupuncture - moxibustion Combined with Citalopram Hydrobromide Tablets on the Depressive State and Sleep of Post - stroke Depression Patients. Clinical Journal of Traditional Chinese Medicine. 2021;13(19):53 - 56.<br><br>604. Yu Na, Fang Sheng, Hu Piaopiao. Clinical Research on the Treatment of Post - stroke Depression with Acupuncture - moxibustion Combined with Escitalopram Oxalate. Journal of New Chinese Medicine. 2023;55(06):145 - 149.<br><br>605. Zhu Jian. Efficacy Analysis of Acupuncture - moxibustion Combined with Medication in the Treatment of Post - stroke Depression. Massage & Rehabilitation Medicine. 2012;3(33).<br><br>606. Chen Kejian. Clinical Influence of Acupuncture - moxibustion Combined with Medication on Emotional Disorders of Post - stroke Depression Patients. Family Medicine. 2019;(9):93.<br><br>607. Jian Rui. Clinical Effect Analysis of Acupuncture - moxibustion Combined with Western Medicine in the Treatment of Post - stroke Depression. Primary Medical Forum. 2020;24(04):560 - 562.<br><br>608. Liu Wei. Clinical Observation on the Treatment of Post - stroke Depression with Acupuncture - moxibustion Combined with Xiaoyao San. China Health Standard Management. 2016;7(17):138 - 140.<br><br>609. Jiang Lan, Tian Huijun, Huang Wei. Efficacy of Acupuncture - moxibustion Combined with Deanxit in the Treatment of Post - stroke Depression and Its Influence on the Serum Neurotransmitter Levels of Patients. Shaanxi Journal of Traditional Chinese Medicine. 2019;40(08):1134 - 1137.<br><br>610. Feng Wan, Yang Yaojun. Influence of Acupuncture - moxibustion Adjuvant Therapy on Serum Cytokines and Neurotransmitters in Post - stroke Depression Patients. Journal of Hainan Medical University. 2017;23(14):2010 - 2013.<br><br>611. Li Hongqi. Influence of Acupuncture - moxibustion Adjuvant Therapy on the Sequelae of Post - stroke Depression Patients, and on Their Neurological Function and Activities of Daily Living. Chinese Science and Technology Journal Database (Abstract Edition) of Medicine and Health. 2023;(2).<br><br>612. Wang Xuan, Shi Fuping, Lu Baohui, Lu Wenxiao, Meng Xiangfei, Liang Xiaofeng. Influence of Acupuncture - moxibustion Adjuvant Therapy on the Sequelae of Post - stroke Depression Patients, and on Their Neurological Function and Activities of Daily Living. Hebei Medical Journal. 2022;44(12):1865 - 1868.<br><br>613. Li Futian, Li Sen, Shi Weilin. Clinical Efficacy of Acupuncture - moxibustion Adjuvant Therapy for Post - stroke Depression Patients. Contemporary Medicine. 2022;28(21):14 - 17.<br><br>614. Deng Lanping, Li Anhong. Discussion on the Mechanism of Treating Post - stroke Depression with the Acupuncture - moxibustion Method of Dredging the

Governor Vessel and Regulating the Mind. Asian Pacific Traditional Medicine. 2016;12(09):118 - 119.

615. Zhang Yanjun, Li Lingfeng, Deng Pengbin. Effect of the Acupuncture - moxibustion Method of Dredging the Governor Vessel and Regulating the Mind in the Treatment of Post - stroke Depression and Its Influence on Symptom Improvement and Serological Indicators. Health Care Literature. 2024;25(4):189 - 192.

616. Zhang Xiaodong, Yao Biqin, Tian Haijuan. Influence of Acupuncture - moxibustion Combined with Paroxetine on Post - stroke Depression Patients. Gansu Science and Technology. 2019;35(05):136 - 137+142.

617. Chang Wei. Treatment of 75 Cases of Post - stroke Depression with Acupuncture - moxibustion Combined with Paroxetine. Shaanxi Journal of Traditional Chinese Medicine. 2009;30(06):712 - 713.

618. Zhang Zhongfa. Treatment of 75 Cases of Post - stroke Depression with Acupuncture - moxibustion Combined with Sertraline. Chinese and Foreign Health Digest. 2012;9(24):152 - 153.

619. Hu Yang, Gao Kun. Evaluation of the Efficacy and Safety of Acupuncture - moxibustion Combined with Western Medicine in the Treatment of Post - stroke Depression. Journal of Yunnan Traditional Chinese Medicine and Materia Medica. 2016;37(02):54 - 55.

620. Peng Jin, Peng Wenda, Zhu Mao, Chen Ling, Gu Xing. Efficacy Observation of Acupuncture - moxibustion in Preventing Post - stroke Depression. Guiding Journal of Traditional Chinese Medicine and Pharmacy. 2016;22(24):58 - 59.

621. Lin Jiewen, Liu Yue, Zeng Kexue. Clinical Observation on the Treatment of 30 Cases of Post - stroke Depression with the Combined Therapy of Acupuncture and Medicine. Jiangsu Journal of Traditional Chinese Medicine. 2013;45(03):60 - 61.

622. Zhou Cuixia, Cui Xiao, Hu Yongshan, et al. Influence of the Combined Therapy of Acupuncture and Medicine on the Comprehensive Function of Post - stroke Depression Patients. Journal of Neurology and Neurorehabilitation. 2009;6(04):255 - 258.

623. Yang Jing, Wang Fei, Xiao Wenhua. Clinical Observation on the Treatment of 33 Cases of Post - stroke Depression with the Combined Therapy of Acupuncture and Medicine. Journal of Emergency in Traditional Chinese Medicine. 2015;24(07):1227 - 1228.

624. Zhou Cuixia, Cui Xiao, Li Xiaoqi, et al. Clinical Research on the Treatment of Post - stroke Depression with the Combined Therapy of Acupuncture and Medicine. Shanghai Journal of Traditional Chinese Medicine. 2010;44(05):46 - 48.

625. Chang Xuehui, Zhang Liangzhi. Clinical Observation on the Treatment of 50 Cases of Post - stroke Depression with the Combined Therapy of Acupuncture and Medicine. Journal of New Chinese Medicine. 2011;43(02):111 - 112.

626. Zhang Wei, Cheng Shaolu, Lin Anji, et al. Influence of the Simultaneous Use of Acupuncture and Medicine on the Depressive State and Activities of Daily Living of Post - stroke Depression Patients. Shanghai Journal of Acupuncture and Moxibustion. 2011;30(12):823 - 825.

	627. Fu Wei. Influence of the Simultaneous Use of Acupuncture and Medicine on the Electroencephalogram and the Levels of Serum NE, NSE, IL - 6, and TNF - a in Post - stroke Depression Patients. Shanghai Journal of Acupuncture and Moxibustion. 2019;38(11):1214 - 1218.<br><br>628. Mai Lanfang, Zhang Xinkai, Zhang Xueyong, Liao Xiaoming, Shen Fei. Evaluation of the Effect of the Simultaneous Use of Acupuncture and Medicine on the Emotional Disorders, Rehabilitation Training Effect, and Quality of Life of Post - stroke Depression Patients. Guangming Journal of Chinese Medicine. 2020;35(03):387 - 389.<br><br>629. Wang Qiaoling. Influence of the Simultaneous Use of Acupuncture and Medicine on the Emotional Disorders, Rehabilitation Training Effect, and Quality of Life of Post - stroke Depression Patients. Research of Integrated Traditional Chinese and Western Medicine. 2017;9(02):73 - 75.<br><br>630. Zhou Cuixia, Cui Xiao, Hu Yongshan, et al. Influence of the Simultaneous Use of Acupuncture and Medicine in the Intervention of Post - stroke Depression on the Self - care Ability in Daily Life. Shanghai Journal of Acupuncture and Moxibustion. 2012;31(04):228 - 230.<br><br>631. Liu Dan, Shan Fangyu. Clinical Efficacy Observation on the Treatment of 20 Cases of Post - stroke Depression with the Combined Therapy of Acupuncture and Medicine. Journal of Clinical Acupuncture and Moxibustion. 2013;29(11):9 - 10.<br><br>632. Guo Aisong, Li Aihong, Gu Yihuang, Jin Hongzhu. Treatment of 30 Cases of Post - stroke Depression with the Combined Therapy of Acupuncture and Medicine. Shaanxi Journal of Traditional Chinese Medicine. 2008;(02):210 - 211.<br><br>633. Zhu Fengkui, Zhao Jun, Ding Yong. Clinical Efficacy Observation on the Treatment of Post - stroke Depression with the Combined Therapy of Acupuncture and Medicine. Journal of Clinical Acupuncture and Moxibustion. 2010;26(05):10 - 11.<br><br>634. Zhang Wei, Lin Anji, Xie Junjie, et al. Clinical Research on the Treatment of Post - stroke Depression with the Combined Therapy of Acupuncture and Medicine. Clinical Journal of Traditional Chinese Medicine. 2014;6(01):14 - 17.<br><br>635. Yang Qingsong. Clinical Observation on the Treatment of 45 Cases of Post - ischemic - stroke Depression with the Combined Therapy of Acupuncture and Medicine. Guiding Journal of Traditional Chinese Medicine and Pharmacy. 2007;(09):51 - 52.<br><br>636. Ye Haimin, Liu Ling. Clinical Observation on the Treatment of Depressive State after Ischemic Stroke with the Combined Therapy of Acupuncture and Medicine. Hubei Journal of Traditional Chinese Medicine. 2008;(01):27 - 28.<br><br>637. Zhang Xin'an. Discussion on the Effect of the Combined Therapy of Acupuncture and Medicine in the Treatment of Post - ischemic - stroke Depression. Contemporary Medicine Forum. 2019;17(23):44 - 45.<br><br>638. Xiao Wei, Wang Ying, Wang Zhen, et al. Treatment of 30 Cases of Post - stroke Depression with the Combined Therapy of Acupuncture and Medicine. Journal of Anhui University of Chinese Medicine. 2011;30(04):44 - 47.<br><br>639. Zhang Zhenwei, Lu Yuexia, Jia Jinhui. Treatment of 36 Cases of Depression after Cerebral Infarction with the Combined Therapy of Acupuncture and Medicine.

	Liaoning Journal of Traditional Chinese Medicine. 2007;(10):1462 - 1463.<br><br>640. Lu Yuexia, Jia Jinhui, Zhang Zhenwei. Treatment of 68 Cases of Depression after Cerebral Infarction with the Combined Therapy of Acupuncture and Medicine. Shaanxi Journal of Traditional Chinese Medicine. 2007;(10):1382 - 1383.<br><br>641. Zhang Zhenwei, Jia Jinhui, Lu Yuexia. Clinical Research on the Treatment of Depression after Cerebral Infarction with the Combined Therapy of Acupuncture and Medicine. Journal of Sichuan of Traditional Chinese Medicine. 2007;(09):108 - 110.<br><br>642. Zhang Zhenwei, Ding Min, Li Mei. Controlled Observation on the Treatment of Depression after Cerebral Infarction with the Combined Therapy of Acupuncture and Medicine. Modern Journal of Integrated Traditional Chinese and Western Medicine. 2005;(08):1003 - 1004.<br><br>643. Xie Xia, Li Wenjuan, Pan Yanjun, Huang Wenqi, Wang Lin. Clinical Efficacy Observation on the Treatment of Post - stroke Depression with the Combined Therapy of Acupuncture and Medicine. Information on Traditional Chinese Medicine. 2020;37(01):92 - 95.<br><br>644. Li Qiang. Research on the Academic Thoughts of Teacher Chen Jutang on the Efficacy of Acupuncture - moxibustion in the Treatment of Post - stroke Depression Patients and Its Influence on the Neurological Function of Patients. Special Health. 2020;(21):22.<br><br>645. Wu Jiali, Pan Weidong, Yu Hailong, He Yufeng. Efficacy Analysis of the Mind - regulating Acupuncture Method of "Jin's Three - needle" on Sleep Disorders in Elderly Post - stroke Depressive State. Clinical Journal of Traditional Chinese Medicine. 2021;13(21):95 - 97.<br><br>646. Wang Yun. Clinical Observation on the Treatment of 30 Cases of Post - stroke Depression with Music Electro - acupuncture Therapy. Jiangsu Journal of Traditional Chinese Medicine. 2018;50(04):62 - 64.<br><br>647. Xiao Wei, Kong Hongbing, Wang Zhen, et al. Clinical Research on the Treatment of Post - stroke Depression with Xiangcongci Combined with Fluoxetine. Journal of Clinical Traditional Chinese Medicine. 2009;21(04):330 - 331.<br><br>648. Cheng Jianming, Jin Shuang, Peng Li. Clinical Observation on the Treatment of Post - stroke Depression with Cranial Suture - piercing Acupuncture Method. Journal of Practical Traditional Chinese Medicine. 2014;30(4).<br><br>649. Wang Yanwu. Treatment of 40 Cases of Post - stroke Depression with High - frequency Electro - acupuncture. Zhejiang Journal of Traditional Chinese Medicine. 2010;45(01):62.<br><br>650. Zhou Han, Peng Jiping, Hu Xiaohong, Yang Feng, Yu Gang. Clinical Efficacy Observation on the Treatment of Post - stroke Depression with the "Thirteen Ghost - gate Needles" Combined with the Method of Soothing the Liver and Regulating the Mind. Chinese Journal of Health Preservation. 2023;41(01):32 - 35.<br><br>651. Xu Jie, Li Lian. Treatment of 75 Cases of Post - stroke Depression with Deanxit Combined with Acupuncture - moxibustion. Journal of Traditional Chinese Medicine. 2008;(10):910 - 911.
SRs/MAs in Chinese	1. Wang Qian, Zheng Shumei, Tang Meixia, Cui Hai. Meta - analysis of Body Acupuncture in the Treatment of Post - stroke Depression. Acta Chinese Medicine. 2016;31(08):1227 - 1231.

	2. Liu Qiang, Li Bo, Wang Xiujuan. Efficacy Evaluation Research on the Treatment of Post - stroke Depression with Acupuncture Based on Cumulative Meta - analysis. Liaoning Journal of Traditional Chinese Medicine. 2018;45(05):897 - 899.<br><br>3. Zhang Huajun, Xu Linling, Xu Tianshu. Systematic Evaluation of the Efficacy of Head Acupuncture in the Treatment of Post - stroke Depression. Journal of Liaoning University of Traditional Chinese Medicine. 2017;19(03):163 - 166.<br><br>4. Li Xiaohui, Chen Junqi, Wang Huiting, Chen Qiuyuan, Wang Shengxu. Systematic Review of the Comparison between Electro - acupuncture and Antidepressants in the Treatment of Post - stroke Depression. Chinese General Practice. 2012;15(07):802 - 806.<br><br>5. Zhan Jie, Tan Feng, Cheng Nanfang, Tan Jiuqing. Systematic Evaluation of the Efficacy of Electro - acupuncture and Western Antidepressants in the Treatment of Post - stroke Depression. Chinese Archives of Traditional Chinese Medicine. 2016;34(10):2379 - 2383.<br><br>6. Yang Yi, Cheng Ziliang, Xu Weiyan, Ye Wenguo, Xiong Jun, Hong Ens. Meta - analysis of the Efficacy and Safety of Electro - acupuncture in the Treatment of Post - stroke Depression. Chinese Medicine Modern Distance Education of China. 2024;22(08):86 - 88 + 117.<br><br>7. Wang Pengqin, Cheng Xiuping. Meta - analysis of the Efficacy of Eye - acupuncture Combined Therapy in the Treatment of Post - stroke Depression. Journal of Liaoning University of Traditional Chinese Medicine. 2019;21(6).<br><br>8. Wang Qiang, Guo Yang, Liu Yao, Wang Yuan. Meta - analysis of Auricular - point Application in the Treatment of Post - stroke Depression. China Medical Herald. 2018;15(04):103 - 107.<br><br>9. Lü Xiaolin, Sun Zhongren, Zhang Qinhong, Du Wei. Clinical Systematic Review of Moxibustion in the Treatment of Post - stroke Depression. Shanghai Journal of Acupuncture and Moxibustion. 2013;32(11):954 - 956.<br><br>10. Wu Yulong, Li Yanjiao, An Junming, Fu Linhui, Sun Hui. Meta - analysis of Head Acupuncture in the Treatment of Post - stroke Depression in the Past Decade. World Chinese Medicine. 2021;16(20):3040 - 3046.<br><br>11. Wu Yuqiu, Yang Nanzhu. Efficacy Analysis of the Brain - Awakening and Orifice - Opening Acupuncture Method in the Treatment of Post - stroke Depression. Journal of Qiqihar Medical University. 2015;36(07):1017 - 1019.<br><br>12. Lin Shaoxia, Lu Chunjian, Zhuo Yuanyuan, Yuan Jinjun, Zhu Yanxian, Pi Min. Meta - analysis of the Efficacy and Safety of the Brain - Awakening and Orifice - Opening Acupuncture Method in the Treatment of Post - stroke Depression. Journal of Guangzhou University of Chinese Medicine. 2022;39(06):1453 - 1460.<br><br>13. Huang Yingjie, Li Lixia, Zhou Yalan, et al. Meta - analysis of the Efficacy of Acupuncture and Electro - acupuncture in the Treatment of Post - stroke Depression. Modern Hospital. 2018;18(01):120 - 124.<br><br>14. Zhang Jianbo, Ren Lu, Sun Yan. Meta - analysis of Acupuncture in the Treatment of Post - stroke Depression. Chinese Acupuncture & Moxibustion. 2009;29(07):599 - 602.

	15. Wang Yiqian, Yuan Chunyu, Jiao Yang, Li Jixin, Huang Hailiang. Systematic Evaluation of the Efficacy and Safety of Acupuncture in the Treatment of Post - stroke Depression. Shandong Journal of Traditional Chinese Medicine. 2021;40(05):464 - 470.<br><br>16. Yin Miaomiao, Xiao Wei, Liang Fajun. Meta - analysis of Acupuncture in the Treatment of Post - stroke Depression. Henan Journal of Traditional Chinese Medicine. 2020;40(11):1729 - 1732.<br><br>17. Yin Miaomiao, Xiao Wei. Meta - analysis of the Efficacy of Acupuncture in the Treatment of Post - stroke Depression. Asian Pacific Traditional Medicine. 2016;12(02):71 - 72.<br><br>18. Xu Ruoxi, Chen Jun. Meta - analysis and Trial Sequential Analysis of Acupuncture in Improving the Depressive State of Post - stroke Depression Patients. Chinese Journal of Gerontology. 2019;39(23):5722 - 5726.<br><br>19. Que Fangxu, Wen Yizhou, Wang Wenchun. Systematic Evaluation of the Efficacy of Acupuncture Compared with Fluoxetine in the Treatment of Post - stroke Depression. Hunan Journal of Traditional Chinese Medicine. 2018;34(09):141 - 145.<br><br>20. Xiong Jun, Du Yuanhao, Liu Jialin, et al. Systematic Review of the Comparison between Acupuncture and Western Medicine in the Treatment of Post - stroke Depression. Evidence - based Medicine. 2010;10(03):179 - 185 + 192.<br><br>21. Nie Rongrong, Fu Wenbin. Systematic Review of the Comparison between Acupuncture and Western Medicine in the Treatment of Post - stroke Depression. World Chinese Medicine. 2012;7(02):147 - 151.<br><br>22. Zhang Mengting, Tan Kang, Hu Sha, Liao Liangying, Shi Wenying, Zhou Wenjuan. Meta - analysis of Acupuncture in Improving the Depressive State and Activities of Daily Living of Post - stroke Depression Patients. Shanghai Journal of Traditional Chinese Medicine. 2021;55(01):13 - 19.<br><br>23. Zhang Guangcai, Huang Yefei, Zhu Xiaoping, Fu Wenbin. Meta - analysis of the Efficacy of Acupuncture in the Treatment of Post - stroke Depression. Journal of New Chinese Medicine. 2011;43(02):127 - 129.<br><br>24. Chen Yamin, Ye Dongzi, Zhang Wei. Meta - analysis of the Clinical Efficacy of the Combined Therapy of Acupuncture and Medicine in the Treatment of Post - stroke Depression. Popular Science & Technology. 2022;24(06):149 - 153 + 106.<br><br>25. Wei Yanying, Tang Chunzhi, Gu Yumei, Wei Sufen. Systematic Review and Meta - analysis of Jin's Three - needle Therapy in the Treatment of Post - stroke Depression. Journal of Liaoning University of Traditional Chinese Medicine. 2018;20(12).
Clinical trials in English	1. Zhao J, Li F. Effect of acupuncture on post-stroke depression. International Journal of Clinical and Experimental Medicine. 2019;12(7):9097-9103.<br>2. Yang T, Cheng R, Zhang J, Tian L, Sun P, Ye X. Effect of Acupuncture Treatment on Post-stroke Depression by Using Diamond-Like Ultra-Thin Nano-Coating Technology. CELLULAR AND MOLECULAR BIOLOGY. 2022;68(3):122-130.<br>3. Lin F, Huang D, He N, Gu Y, Wu Y. Effect of music therapy derived from the five elements in Traditional Chinese Medicine on post-stroke depression. Journal of traditional Chinese medicine / Chung i tsa chih ying wen pan. 2017;37(5):675-680.<br>4. Li M, Zhang B, Meng Z, et al. Effect of Tiaoshen Kaiqiao acupuncture in the treatment of ischemic post-stroke depression: a randomized controlled trial. Journal of

	Traditional Chinese Medicine. 2017;37(2):171-178.<br>5. Youn JI, Sung KK, Song BK, Kim M, Lee S. Effects of electro-acupuncture therapy on post-stroke depression in patients with different degrees of motor function impairments: a pilot study. J Phys Ther Sci. 2013;25(6):725-728.<br>6. Cai W, Ma W, Li YJ, Wang GT, Yang H, Shen WD. Efficacy and safety of electroacupuncture for post-stroke depression: a randomized controlled trial. Acupuncture in Medicine. 2022;40(5):434-442.<br>7. Zhi Y, Huo XH, Li P. Efficacy observation of Zhi Shen Tiao Sui acupuncture method for depression after ischemic stroke. Journal of Acupuncture and Tuina Science. 2021;19(3):180-186.<br>8. Ma F, Cao G, Lu L, Zhu Y, Li W, Chen L. Electroacupuncture versus Escitalopram for mild to moderate Post-Stroke Depression: a randomized non-inferiority trial. Front Psychiatry. 2024;15.<br>9. Sang P, Zhao J, Wang S, Yang H, Shi H. Influence of acupuncture on patients with post-stroke depression and through p11/tPA/BDNF pathway genes. International Journal of Clinical and Experimental Medicine. 2020;13(3):1995-2001.<br>10. Wang H, Li Y. A pilot controlled trial of a combination of electroacupuncture and psychological intervention for post-stroke depression. COMPLEMENTARY THERAPIES IN MEDICINE. 2022;71:102899.<br>11. Tseng SP, Hsu YC, Chiu CJ, Wu ST. A Population-Based Cohort Study on the Ability of Acupuncture to Reduce Post-Stroke Depression. Medicines (Basel). 2017;4(1).<br>12. Zhang DR, Peng X, Zhi Y. Therapeutic effect observation on acupuncture plus medication for post-stroke depression. Journal of Acupuncture and Tuina Science. 2019;17(1):31-36.<br>13. Qian X, Zhou X, You Y, et al. Traditional Chinese Acupuncture for Poststroke Depression: a Single-Blind Double-Simulated Randomized Controlled Trial. Journal of alternative and complementary medicine (New York, N.Y.). 2015;21(12):748-753.<br>14. You Y, Zhang T, Shu S, Qian X, Zhou S, Yao F. Wrist-ankle acupuncture and Fluoxetine in the treatment of post-stroke depression: a randomized controlled clinical trial. Journal of Traditional Chinese Medicine. 2020;40(3):455-460.<br>15. Xie J, Li J, Sun Q, Cai J. Effect of traditional Chinese medicine-based rehabilitation nursing combined with scalp acupuncture on negative emotions and quality of life of patients with stroke: a randomized controlled trial. MEDICINE. 2022;101(43):e31330.
SRs/MA in English	1. Zhang GC, Fu WB, Xu NG, et al. Meta analysis of the curative effect of acupuncture on post-stroke depression. Journal of Traditional Chinese Medicine. 2012;32(1):6-11.<br>2. Liu R, Zhang K, Tong QY, Cui GW, Ma W, Shen WD. Acupuncture for post-stroke depression: a systematic review and meta-analysis. BMC Complementary Medicine and Therapies. 2021;21(1).<br>3. Li XB, Wang J, Xu AD, et al. Clinical effects and safety of electroacupuncture for the treatment of post-stroke depression: a systematic review and meta-analysis of randomised controlled trials. Acupuncture in Medicine. 2018;36(5):284-293.<br>4. Zhang L, Chen B, Yao Q, et al. Comparison between acupuncture and antidepressant therapy for the treatment of poststroke depression: Systematic review and meta-analysis. Medicine (United States). 2021;100(22):E25950.<br>5. Zhang K, Cui G, Gao Y, Shen W. Does acupuncture combined with antidepressants have a better therapeutic effect on post-stroke depression? A systematic review and meta-analysis. Acupuncture in Medicine. 2021;39(5):432-440.<br>6. Zhang J, Chen J, Chen J, et al. Early filiform needle acupuncture for poststroke depression: a meta-analysis of 17 randomized controlled clinical trials. Neural Regeneration Research. 2014;9(7):773�\784.<br>7. Jiang W, Jiang X, Yu T, Gao Y, Sun Y. Efficacy and safety of scalp acupuncture for poststroke depression: A meta-analysis and systematic review. Medicine (United States). 2023;102(31):E34561.<br>8. Zhong D, Cheng H, Pan Z, et al. Efficacy of scalp acupuncture combined with

	conventional therapy in the intervention of post-stroke depression: A systematic review and meta-analysis. COMPLEMENTARY THERAPIES IN MEDICINE. 2023;77.<br>9. Guo SQ, Zhao GZ, Li ST, et al. Moxibustion for treating patients with post-stroke depression: a systematic review and meta-analysis. Ann Palliat Med. 2022;11(1):85-97.

Appendix 3.Distribution and situation of countries and regions

Country	Count	Proportion
South Korea	1	0.14%
United States	1	0.14%
Singapore	1	0.14%
China	697	99.57%
**Province of origin in China**	**Count**	**Proportion**
Anhui	31	4.45%
Beijing	34	4.88%
Chongqing	7	1.00%
Fujian	23	3.30%
Gansu	10	1.43%
Guangdong	75	10.76%
Guangxi	11	1.58%
Guizhou	5	0.72%
Hainan	11	1.58%
Hebei	54	7.75%
Henan	40	5.74%
Heilongjiang	55	7.89%
Hubei	27	3.87%
Hunan	23	3.30%
Jilin	13	1.87%
Jiangsu	43	6.17%
Jiangxi	6	0.86%
Liaoning	28	4.02%
Neimenggu	4	0.57%
Ningxia	1	0.14%
Shandong	33	4.73%
Shanxi	10	1.43%
Shanxi	17	2.44%
Shanghai	22	3.16%
Sichuan	23	3.30%
Taiwan	1	0.14%
Tianjin	44	6.31%
Xinjiang	6	0.86%
Yunnan	5	0.72%
Zhejiang	35	5.02%
**Multi-agency collaboration**	**Count**	**Proportion**
NO	71.14%	71.14%
YES	28.86%	28.86%
**Multi-national cooperation**	**Count**	**Proportion**
NO	99.71%	99.71%
YES	0.29%	0.29%
**Inter-provincial cooperation**	**Count**	**Proportion**
NO	96.14%	96.14%
YES	3.86%	3.86%

Appendix 4 Overview of other original study interventions

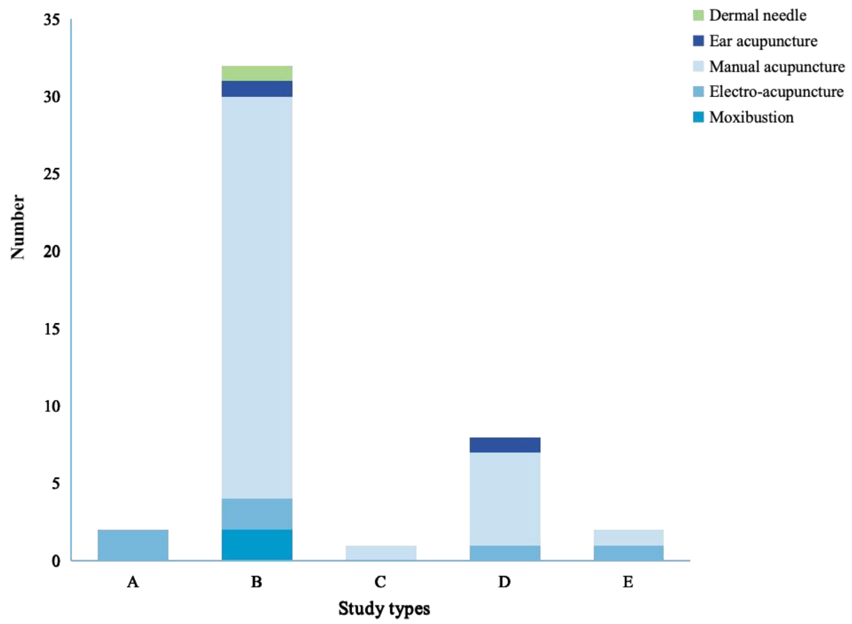

NOTE：A：Case Control Study；B：Single arm study；C：Cohort Study；
D：Non-randomized Controlled Study；E：retrospective case series study；

Appendix 5. Frequency statistics of acupoint related information

Content		Count	Content		Count
Acupoint	GV 20	438	Body parts	Head	1650
	PC6	305		Forethigh	624
	LR3	298		Foot	491
	EX-HN1	252		Lower leg	439
	HT7	227		Back	348
	GV 24	208		Hand	276
	EX-HN3	201		Ear	190
	SP-6	183		Abdomen	145
	LI4	171		Chest	131
	ST36	148		Neck	118
Meridian	DU	945	Specific points	Back-shu point	242
	The Extra Ordinary Points	505		Eight confluence points	374
	PC	354		Eight influential points	165
	BL	348		Front-mu point	172
	LR	345		He-sea point	234
	LI	287		Jing-river point	41
	ST	282		Jing-well point	154
	HT	260		Lower-he-sea point	148
	SP	257		Luo-connecting point	402
	GB	251		Shu-stream point	574

NOTE:
Acupuncture points:GV20:Baihui,PC6:Neiguan , LR3:Taichong , EX-HN1:Sishencong , HT7:Shenmen, GV24Shenting, EX-HN3:Yingtang, SP6:Sanyinjiao , LI4:Hegu , ST36:Zusanli.

Meridians:CV: Conception Vessel; BL: Bladder meridian of foot-taiyang; ST:Stomach meridian of foot-yangming; SP: Spleen meridian of foot-taiyin; kl: Kidneymeridian of foot-shaoyin; GV: Governor vessel; LR: Livermeridian of foot-jueyin; GB: Gallbladder meridian of foot-shaoyang; Ll: Large intestinemeridian of and-yangming; PC:Pericardium meridian of hand-jueyin;HT: Heart meridianof hand-shaoyin; LU: Lung meriof hand-taiyin;

Appendix 6. Intervention duration of clinical studies

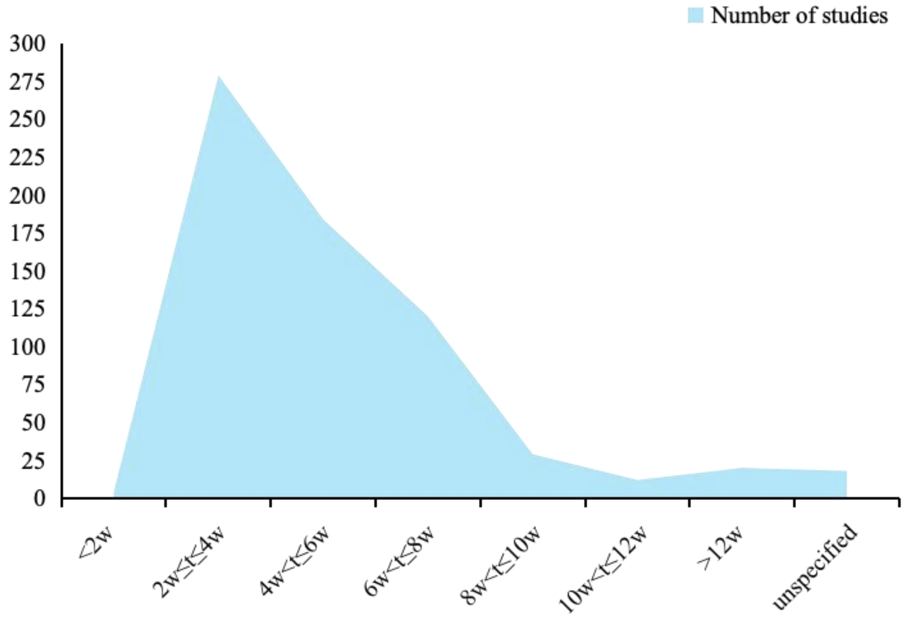

Appendix 7 Assessment of risk of bias in clinical randomized controlled trials

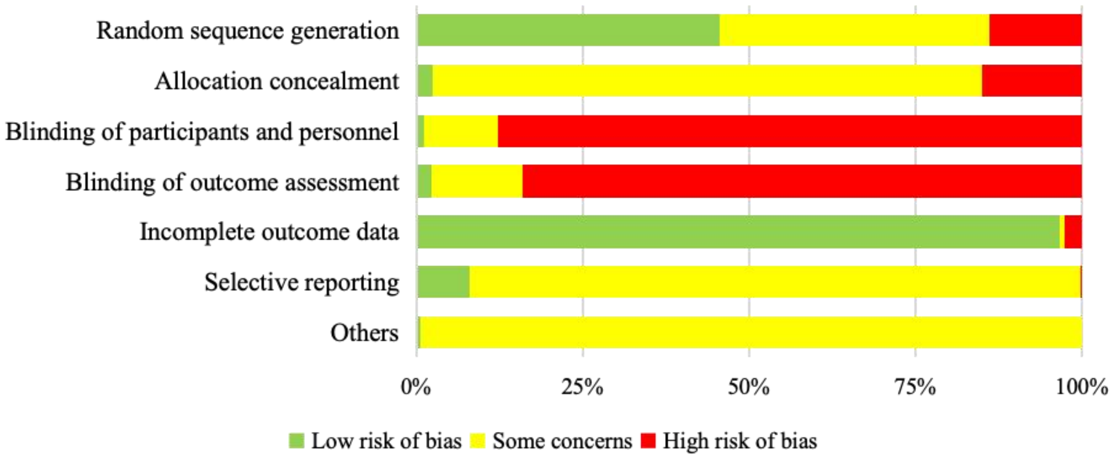

Appendix 8. Quality assessment of SRs/MAs

References \ Item	Item1	Item2	Item3	Item4	Item5	Item6	Item7	Item8	Item9	Item10	Item11	Item12	Item13	Item14	Item15	Item16
Wang2016	Yes	No	Yes	Partial Yes	Yes	Yes	Yes	Partial Yes	Yes	No	Yes	Yes	Yes	No	Yes	No
Que2018	Yes	No	Yes	Partial Yes	Yes	Yes	Partial Yes	Partial Yes	Yes	No	Yes	No	Yes	No	No	No
Yin2020	Yes	No	Yes	Partial Yes	Yes	Yes	Partial Yes	Partial Yes	Yes	No	Yes	No	Yes	No	No	No
Yin2016	Yes	No	Yes	Partial Yes	No	No	Partial Yes	Partial Yes	No	No	No	No	Yes	No	No	No
Xu2019	Yes	No	Yes	Partial Yes	Yes	Yes	Partial Yes	Partial Yes	Yes	No	Yes	No	Yes	No	Yes	No
Wang2021	Yes	No	Yes	Partial Yes	Yes	No	Partial Yes	Partial Yes	Yes	No	Yes	Yes	Yes	No	Yes	No
Zhang2021	Yes	No	Yes	Partial Yes	Yes	Yes	Partial Yes	Yes	Yes	No	Yes	Yes	Yes	No	Yes	No
Wang2018	Yes	No	Yes	Partial Yes	Yes	Yes	Partial Yes	Partial Yes	Yes	No	Yes	No	Yes	No	Yes	No
Liu2018	Yes	No	Yes	Partial Yes	Yes	Yes	Partial Yes	Partial Yes	No	No	No	No	No	No	No	No
Lin2021	Yes	No	Yes	Partial Yes	Yes	Yes	Partial Yes	Partial Yes	Yes	No	Yes	No	No	Yes	Yes	No
Wang2019	Yes	No	No	Partial Yes	Yes	Yes	Partial Yes	Partial Yes	Yes	No	Yes	No	Yes	No	Yes	No
Huang2018	Yes	No	Yes	Partial Yes	Yes	Yes	Partial Yes	Partial Yes	Yes	No	Yes	Yes	Yes	Yes	No	No
Wei2018	Yes	No	Yes	Partial Yes	Yes	Yes	Partial Yes	Partial Yes	Yes	No	Yes	No	Yes	No	Yes	No
Wu2021	Yes	No	Yes	Partial Yes	Yes	Yes	Partial Yes	Partial Yes	Yes	No	Yes	No	Yes	No	Yes	No
Zhang2017	Yes	No	Yes	Partial Yes	Yes	Yes	Partial Yes	Partial Yes	Yes	No	Yes	Yes	Yes	Yes	Yes	No
Li2012	Yes	No	Yes	Partial Yes	Yes	Yes	Partial Yes	Partial Yes	Yes	No	Yes	Yes	Yes	No	Yes	No
Zhan2016	Yes	No	Yes	Partial Yes	Yes	Yes	Partial Yes	Partial Yes	Yes	No	Yes	Yes	Yes	Yes	Yes	No
Yang2024	Yes	No	Yes	Partial Yes	Yes	Yes	Partial Yes	Partial Yes	Yes	No	Yes	Yes	Yes	Yes	Yes	No
Lu2013	Yes	No	Yes	Partial Yes	Yes	Yes	Partial Yes	Partial Yes	Yes	No	Yes	Yes	Yes	Yes	Yes	No
Wu205	Yes	No	Yes	Partial Yes	Yes	Yes	Partial Yes	Partial Yes	Yes	No	Yes	Yes	Yes	Yes	Yes	No
Zhang2009	Yes	No	Yes	Partial Yes	Yes	Yes	Partial Yes	Partial Yes	Yes	No	Yes	Yes	Yes	Yes	Yes	No
Xiong2010	Yes	No	Yes	Partial Yes	Yes	Yes	Partial Yes	Partial Yes	Yes	No	Yes	No	Yes	Yes	Yes	No
Zhang2011	Yes	No	Yes	Partial Yes	Yes	Yes	Partial Yes	Partial Yes	Yes	No	Yes	No	Yes	No	Yes	No
Chen2022	Yes	No	Yes	Partial Yes	Yes	Yes	Partial Yes	Partial Yes	Yes	No	Yes	Yes	Yes	Yes	Yes	No
Nie2012	Yes	No	Yes	Partial Yes	Yes	Yes	Partial Yes	Partial Yes	Yes	No	Yes	Yes	Yes	No	Yes	No
Jiang2023	Yes	Yes	Yes	Partial Yes	Yes	Yes	Yes	Partial Yes	Yes	No	Yes	Yes	Yes	Yes	Yes	Yes
Zhong2023	Yes	Yes	Yes	Partial Yes	Yes	Yes	Yes	Partial Yes	Yes	No	Yes	Yes	Yes	Yes	Yes	Yes
Guo2022	Yes	Yes	No	Partial Yes	Yes	Yes	Yes	Partial Yes	Yes	No	Yes	No	Yes	Yes	Yes	Yes
Liu2021	Yes	Yes	Yes	Partial Yes	Yes	Yes	Yes	Partial Yes	Yes	No	Yes	Yes	Yes	Yes	Yes	Yes
Zhang2021	Yes	No	Yes	Partial Yes	Yes	Yes	Yes	Partial Yes	Yes	No	Yes	Yes	Yes	Yes	Yes	Yes
Zhang2021	Yes	Yes	Yes	Partial Yes	Yes	Yes	Yes	Partial Yes	Yes	No	Yes	Yes	Yes	Yes	Yes	Yes
Li2018	Yes	Yes	Yes	Partial Yes	Yes	Yes	Yes	Partial Yes	Yes	No	Yes	Yes	Yes	Yes	Yes	Yes
Zhang2014	Yes	No	Yes	Partial Yes	Yes	Yes	Yes	Partial Yes	Yes	No	Yes	Yes	Yes	Yes	Yes	Yes
Zhang2012	Yes	No	Yes	Partial Yes	Yes	Yes	Yes	Partial Yes	Yes	No	Yes	Yes	Yes	Yes	Yes	Yes

Legend: Yes / Partial Yes / No

NOTE: The methodological quality of the included systematic reviews was assessed using the AMSTAR-2 tool. The above 16 items were evaluated from 16 items of the AMSTAR-2 scale, of which 2, 4, 7, 9, 11, 13, and 15 were the key parts.
